# Relevant Metal Oxidation States of MAO-Activated Chromium Catalysts for Ethylene Oligomerization

**DOI:** 10.1021/acs.inorgchem.6c00208

**Published:** 2026-03-25

**Authors:** Alexander Allgaier, Felix R. Fischer, Somnath Bhattacharya, Kevin Balliet, Michael R. Buchmeiser, Matthias Bauer, Joris van Slageren

**Affiliations:** † Institute of Physical Chemistry, 9149 University of Stuttgart, Pfaffenwaldring 55, Stuttgart D-70569, Germany; ‡ Chemistry Department and Center for Sustainable Systems Design (CSSD), 26578 Paderborn University, Warburger Str. 100, Paderborn D-33098, Germany; § Institute of Polymer Chemistry, University of Stuttgart, Pfaffenwaldring 55, Stuttgart D-70569, Germany

## Abstract

Chromium-catalyzed ethylene oligomerization is an industrially important reaction, but improving the product specificity remains essential. In-depth spectroscopic and theoretical investigations of this catalytic reaction have allowed a great deal of insight into it. However, fundamental issues, such as the oxidation states relevant to the catalysis, are still unclear. This study makes the case for high-frequency electron paramagnetic resonance spectroscopy as a powerful method for studying this catalytic reaction, profiting from high *g*-value resolution and access to large energy splittings. The results confirm the occurrence of chromium­(I) species but also show that such species are not necessarily dead ends in the catalytic cycle. Second, no unambiguous evidence for the relevance of chromium­(II) was found, in spite of the unequivocal ability of HFEPR to detect such species. X-ray Absorption Spectroscopy (XAS) and TD-DFT calculations enabled the structural and electronic ground-state characterization of the dimeric, chloride-bridged Cr­(III) precatalyst **Cr-NHC-N** as a precondition for its following HFEPR investigation.

## Graphical abstract

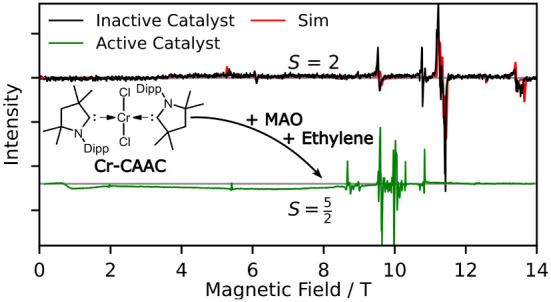

## Introduction

The oligomerization of ethylene to linear α-olefins (LAOs) is a process of great industrial importance because these chemicals are widely used in the production of polymers, plasticizers, detergents, and lubricants. In particular, 1-hexene and 1-octene are valuable comonomers in the manufacturing of linear low-density polyethylene (LLDPE).^1,2^ Chromium-based catalysts are the most active and selective for the specific production of 1-hexene and 1-octene and outperform catalytic systems based on Ni, Fe, and Co.^1,3^ Homogeneous catalysts for ethylene oligomerization are industrially relevant because their selectivities are typically higher than for heterogeneous processes, as demonstrated in the Chevron-Phillips process that achieves reported selectivities for 1-hexene of 93–99%.^1,4–6^ A wide variety of ligand systems have been developed to tune ethylene oligomerization catalysts, with PNP-type (diphosphinoamine) ligands being particularly prominent for achieving high productivities and selectivities toward 1-hexene and 1-octene.^1,7^ The high selectivity of these chromium systems is generally explained by invoking a metallacyclic mechanism, which contrasts with the Cossee–Arlman mechanism that produces a statistical Schulz-Flory distribution of olefins.^3,8^ The metallacyclic mechanism involves the oxidative coupling of two ethylene molecules to form a chromacyclopentane intermediate, causing the chromium oxidation state to increase by 2. This intermediate can then incorporate subsequent ethylene molecules to form larger metallacycles, such as a chromacycloheptane or chromacyclononane, from which 1-hexene and 1-octene are selectively released, respectively.^1,9^

Reported precatalysts feature chromium ions in the +2 or +3 oxidation states.^8,10^ Precatalysts are typically activated with an aluminum-based cocatalyst such as methylalumoxane (MAO), leading to methylation and/or reduction of the precatalyst. However, detailed information on the nature of activated catalysts has not been reported. In fact, the oxidation state of the catalytically active chromium center remains a subject of intense debate,^1,11^ and it is uncertain whether the catalytic cycle proceeds through Cr^+^/Cr^3+^ or Cr^2+^/Cr^4+^ intermediate states.^1,7,12^ The majority of experimental and computational studies appear to support a Cr^+^/Cr^3+^ cycle, but no definitive proof has been published.^1,7,13,14^ To resolve this fundamental question, numerous spectroscopic and theoretical studies have been carried out in recent years,^1,15^ where Electron Paramagnetic Resonance (EPR)^15–18^ and X-ray Absorption Spectroscopy (XAS)^19–22^ have particularly delivered helpful insight. However, direct observation and unambiguous characterization of the catalytically active chromium species are challenging because of the reaction conditions (elevated temperatures and high pressures) that are challenging to combine with many spectrometers and the occurrence of many paramagnetic chromium species, not all of which can be detected by conventional EPR.^8,13^ In fact, potentially key species feature integer spin states, namely, chromium­(II) (*S* = 2) and chromium­(IV) (*S* = 1). Such integer-spin ions are often considered “EPR silent” in conventional X-band (9.5 GHz) EPR due to sizable zero-field splitting, leading to the absence of EPR-allowed transitions within the available magnetic field range. However, high-frequency EPR (HFEPR) can make these spin states spectroscopically accessible, thus offering a key advantage for these investigations.^23–28^ HFEPR measurements on chromium-based ethylene oligomerization catalysts have not yet been reported, to the best of our knowledge. The closest study is an HFEPR study of the active site of the (heterogeneous) Phillips ethylene *polymerization* catalyst (not to be confused with the Chevron-Phillips ethylene *oligomerization* catalyst).^28,29^ Furthermore, (frozen) solution HFEPR spectra of chromium complexes are very rare.^30^ XAS delivers element specific information on oxidation states, coordination numbers, and coordination geometries. In contrast to other spectroscopic approaches, XAS is not affected by the aggregation state of the analyzed matter and therefore applicable to a variety of experimental conditions.^31^ XAS is also the method of choice to obtain both electronic and structural information about solid compounds that are not crystalline or reaction intermediates that cannot be isolated. XAS investigations of Cr-acac^17,32^ and Cr-PNP^17,19–21^ have already been reported.

In this work, we investigate different Cr­(II)- and Cr­(III)-based ethylene oligomerization catalysts and study them with advanced X-ray Absorption Spectroscopy (XAS) techniques and HFEPR. Second, we employ HFEPR to probe the species resulting from MAO activation and interaction with ethylene. These frozen-solution HFEPR measurements provide detailed insights into the spin states and electronic structures of the resulting chromium complexes and highlight the impact of MAO and ethylene on speciation, offering new evidence in the ongoing debate over the active oxidation states in ethylene oligomerization. A preliminary account of this work has appeared on the ChemRxiv preprint server.^33^

## Results and Discussion

For our investigations, we selected a range of chromium-based precatalysts (Figure 1), ranging from established to more recent examples, featuring different chromium oxidation states, to ensure general validity of the obtained results. **Cr-acac** ([Cr^3+^(acac)_3_], acac^–^ = acetyl acetonate) was originally used as a catalyst precursor for ethylene tetramerization^9^ and has been employed as a reference system in detailed spectroscopic studies of ethylene oligomerization catalysts.^16,17^ **Cr-PNP** ([{Cr^3+^(PNP)­(Cl)_2_}_2_(μ-Cl)_2_], PNP = Ph_2_PN­(^i^Pr)­PPh_2_) is a well-established dimeric precatalyst that is selective toward 1-octene over 1-hexene^9^ and has been studied in detail spectroscopically.^16,17,19–22^ **Cr-CAAC** ([Cr^2+^(CAAC)_2_(Cl)_2_], CAAC = 1-(2,6-diisopropylphenyl-1-yl)-3,3,5,5-tetramethyltetrahydropyrrol-2-ylidene) is a chromium­(II) species whose reduced species were studied by EPR and magnetometry.^34^ Furthermore, **Cr-CAAC** was previously shown to be active in ethylene oligomerization, with selectivity toward higher alkenes (C_10_–C_14_).^35^ Recently, novel N-heterocyclic carbene (NHC) complexes of chromium have been proposed as ethylene oligomerization catalysts.^35,36^ Here, we study an NHC-chromium complex with a chelating N-donor, **Cr-NHC-N** ([{Cr^3+^(NHC-N)­(Cl)}_2_(μ-Cl)_2_}] NHC-N^–^ = 1-methyl-3-(2-amido-*N*-(2,6-diisopropylphenyl-1-yl)­phen-1-ylimidazol-2-ylidene),^35^ as well as an NHC-chromium complex with a chelating O-donor, **Cr-NHC-O** ([{Cr^+^(MeCN)}­(μ-NHC-O)_2_(μ-Cl)­{Cr^3+^(Cl)}, NHC-O^–^ = 1-(mesityl)-3-(2-*O*-phenyl)-4,5-dihydroimidazol-2-ylidene).^36^ In the following, we first investigate the geometric and electronic structures of the catalysts by means of advanced XAS techniques and then proceed to investigate by HFEPR the influence of MAO activation and interaction with ethylene on the spin state and electronic structure of the studied complexes.

**Figure 1:**
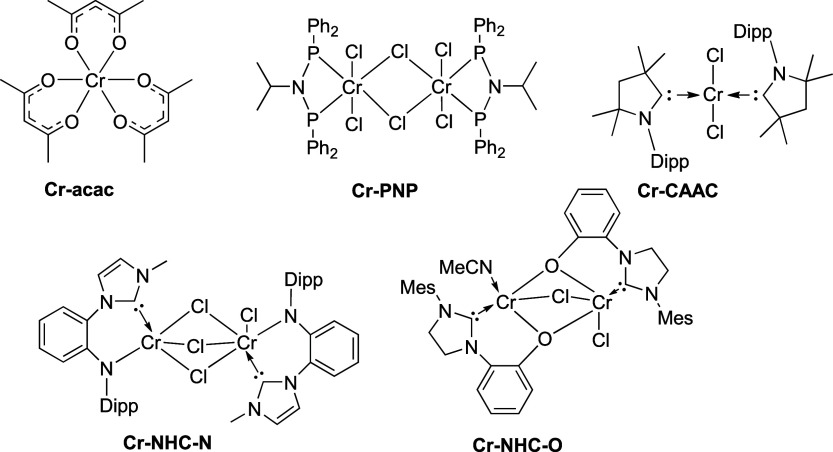
Chemical structures of the chromium complexes studied in this work.

### XAS Investigations

In this work, we carried out XAS studies of both **Cr-CAAC** and **Cr-NHC-N**. Figure 2 displays the normalized and background-subtracted X-ray absorption near edge structure (XANES) spectra of both compounds. Both spectra display pronounced pre-edge features and well-resolved post-edge structures.

**Figure 2:**
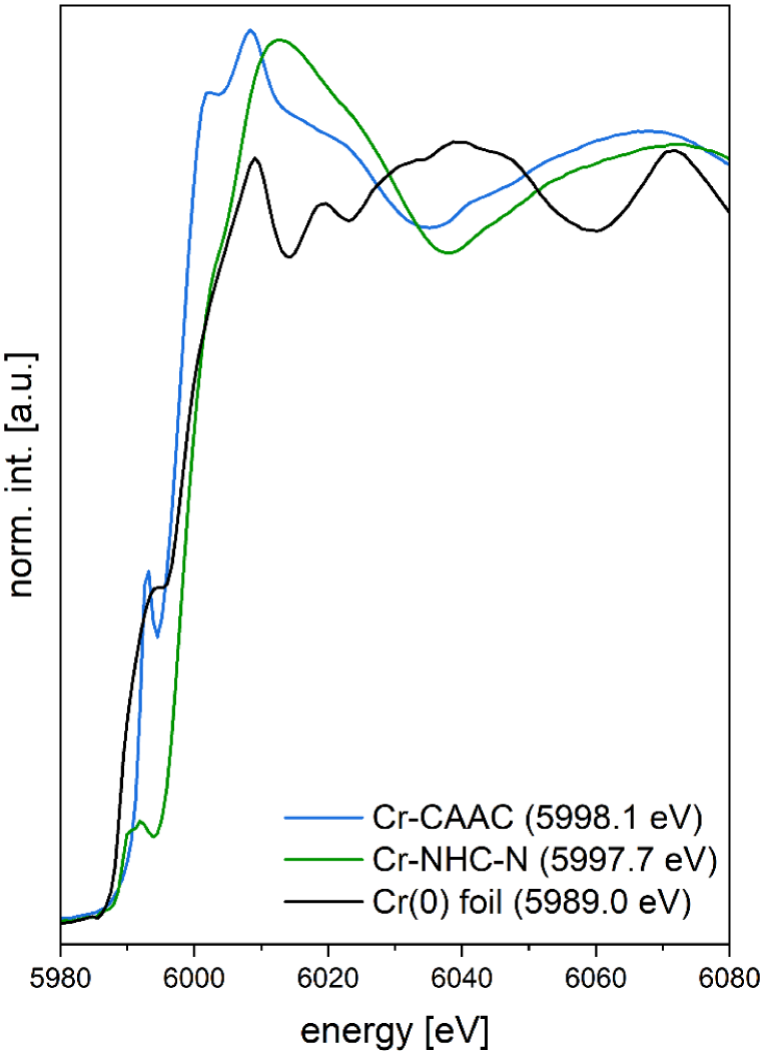
Experimental Cr-K-edge XANES spectra of **Cr-CAAC** (blue), **Cr-NHC-N** (green), and chromium(0) foil (black) in the solid state with absorption edge energies *E*_0_ in brackets. *E*_0_ values correspond to absorption edge energies of other Cr^2+^/Cr^3+^ references reported in literature.^43^

The pre-edge region in XANES spectra originates from electronic transitions from the 1s-core orbital to the lowest unoccupied molecular orbitals (LUMOs), which typically feature substantial d-orbital contributions for transition metals. Although 1s → (*n*–1)­d transitions are dipole-forbidden, their intensity is enhanced by hybridization with metal-centered *n*p-orbitals in noncentrosymmetric symmetries. Additional intensity enhancement might be caused by ligand-induced hybridization with p-orbitals belonging to the donor atoms in the first ligand sphere, thereby providing structural insight into the coordination geometry of the complex.^31,37,38^ Furthermore, the energies of the pre-peaks and of the absorption edge in a XANES spectrum correlate with the electron density of the excited atom and thus with its oxidation state.^31^ Especially, the Cr-K-edge position is strongly sensitive toward modifications in the ligand sphere.^39,40^ For this reason, the order of *E*_0_-values observed for Cr complexes with different coordination geometries and ligand environments does not necessarily reflect their respective formal oxidation states (see comparison of *E*_0_-values for **Cr-CAAC** and **Cr-NHC-N** in Figure 2).^39,40^ Detailed structural information about the type, number, and bond lengths of the coordinating atoms can be obtained from the analysis of extended X-ray absorption fine structure (EXAFS). For the elucidation of the bonding motif between the metal centers and its coordinating donor in an unknown compound, a reasonable starting structure is needed to calculate the required scattering paths.^41,42^ Such a starting geometry is either provided by crystal data (**Cr-CAAC**) or, if unavailable (**Cr-NHC-N**), obtained through geometry optimization by means of DFT.

### Cr-CAAC

The crystal structure of **Cr-CAAC** is known, and, therefore, the calculation of an optimized geometry is not essential, in contrast to the case of **Cr-NHC-N**. However, to validate the procedure, we treated both complexes in the same manner. First of all, the geometry of **Cr-CAAC** was optimized by using the PBEh-3c composite method, where the overall multiplicity was set to *M* = 5, in accordance with a high-spin *S* = 2 state for the chromium­(II) ion (see magnetic measurements below). The relevant geometric parameters were determined (Figure S2); in particular, Cr–C and Cr–Cl bond lengths agree well between experiment and theory (Table 1). In addition, the intense pre-peak feature at *E* ≈ 5993 eV observed in the XANES spectrum of **Cr-CAAC** is reproduced in a TPSSH-based TD-DFT calculation using the obtained structural model (Figure 3). Two electronic transitions at 5992.7 eV and 5993.4 eV were identified as the main contributors to this feature. Their corresponding final states exhibit predominant Cr 4p character, mixed with a significant contribution from the p- and s-orbitals of the C and H atoms in the CAAC ligands (Figures 3, S11 and Table S2). In both transitions, the chromium 1s electrons are strongly delocalized over both carbene ligands, indicating a pronounced orbital interaction between the donor atoms and the chromium center. This is consistent with the well-known strong σ-donor properties of CAAC ligands.^44,45^ The TD-DFT results strongly support the interpretation that the chromium carbene bonding motif has a decisive influence on the energy of the K-edge absorption in Cr-CAAC, in agreement with the fact that CAAC ligands have even been shown to surpass Arduengo-type NHCs in σ-donor strength.^44,45^ Similar results have been reported for a cationic molybdenum alkylidyne complex immobilized inside regular meso- and macroporous silica-60.^46^ Based on the resulting optimized model, the subsequent EXAFS analysis yields coordination numbers and bond lengths for the donor atoms in the first coordination sphere that are in good agreement with the corresponding crystallographic data (Table 1, Figure S13, Table S3 and Table S4).^35^

**Table 1:** First Shell Coordination Numbers (*N*), Bond Lengths (*R* + Δ*R*, Where *R* Is the Distance from the Crystal Structure and Δ*R* Is the Difference between the Bond Length from the EXAFS Fit and That Crystal Structure Bond Length), and Debye–Waller Factors (σ^2^) of **Cr-CAAC** and **Cr-NHC-N**, as Well as Bond Lengths from Crystallographic Data (*R*_exp_) and from DFT-Optimized Structural Models (*R*_th_)­^a^

**Scattering path**	***N***	***R*** **+ Δ** ***R*** **/Å**	**σ** ^**2**^ **/Å** ^**2**^	***R***_**exp**_/Å^b^	***R***_**th**_/Å^c^
**Cr-CAAC**					
Cr–C	2.2(1)	2.064(11)	0.0069(7)	2.173, 2.174	2.177, 2.173
Cr–Cl	2.1(1)	2.333(5)	0.0042(2)	2.330, 2.338	2.344, 2.346
**Cr-NHC-N**					
Cr–N	1.9(1)	1.957(4)	0.0036(4)	–	
Cr–Cl	0.8(1)	2.120(7)	0.0028(4)	–	
Cr–Cl	2.7(1)	2.357(6)	0.0051(7)	–	
Cr–Cr	1.0(1)	3.268(11)	0.0075(10)	–	

a Several DFT-optimized structural models for **Cr-NHC-N** were calculated and are collected in Table S1.

b From the single-crystal structure.

c Several DFT-optimized structural models for **Cr-NHC-N** were calculated (Table S1).

**Figure 3:**
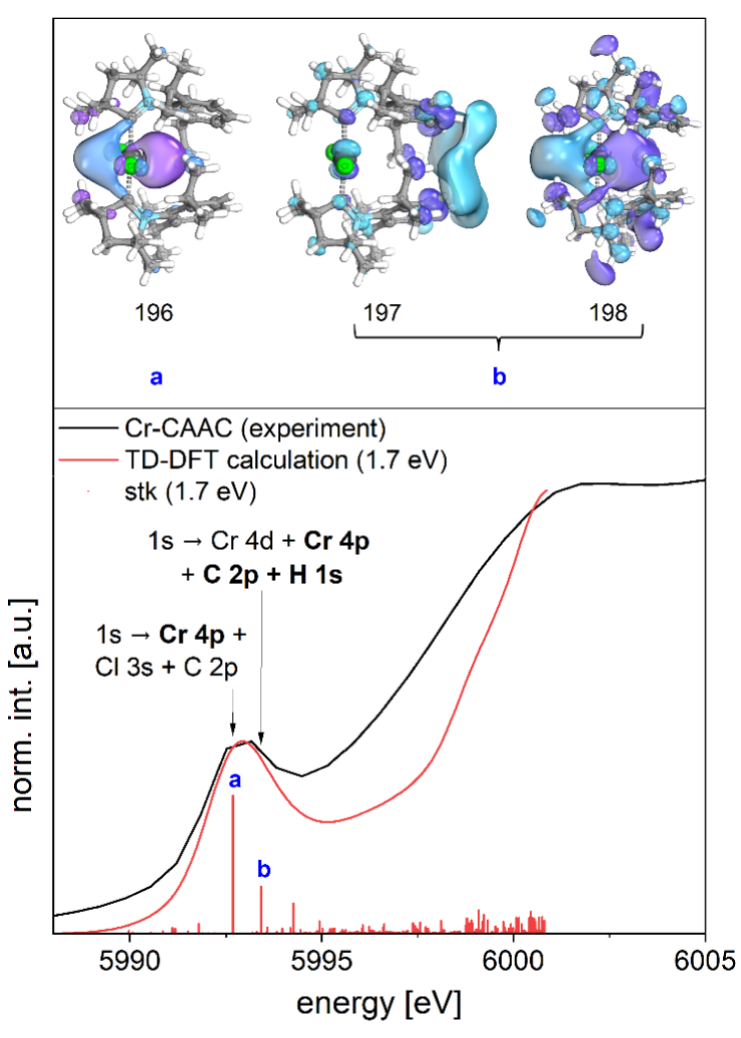
Top: Visualization of the three TD-DFT-based molecular acceptor orbitals (196, 197, 198; see also Figure S11) mainly involved in the pre-edge transitions “a” and “b”. Bottom: Comparison of the pre-edge part of the experimental XANES spectrum of **Cr-CAAC** (black) with TD-DFT calculation (red, 1.7 eV broadening) and main transition assignment (a, b). The calculated transitions are depicted as stick spectra.

### Cr-NHC-N

Having demonstrated that PBEh-3c-based geometry optimizations provide reliable starting points for the EXAFS- and TD-DFT-XANES analyses of **Cr-CAAC**, the same approach was applied to the dimeric complex **Cr-NHC-N**. Geometry optimizations with the PbEH-3c composite method were performed for **Cr-NHC-N** with low- (*M* = 1) and high-spin (*M* = 7) configurations. Furthermore, broken symmetry (BS) geometry optimizations with two antiferromagnetically coupled chromium­(III) centers, each with *S* = 3/2, were carried out using the B3LYP and B3LYP/G functionals (for further details, see Supporting Information). For all spin configurations, both doubly and triply Cl-bridged Cr-dimer were identified as possible structural motifs, consistent with the elemental analysis of **Cr-NHC-N** (Figure 4, Table S1, Figure S1, Figures S3–S10). In addition to distinct changes in the coordination geometry around both chromium centers, doubly and triply bridged structures exhibit significant differences in the Cr–Cr distances, whose exact value depends on the chosen overall multiplicity of the dimer. While the Cr–Cr distance in the doubly bridged species ranges from 3.417 to 3.650 Å, depending on the overall spin multiplicity, the corresponding distance in the triply bridged isomer is significantly shorter, varying between 2.801 and 3.309 Å (Table S1). The experimental EXAFS data are best fitted by combining the N- and C-donor atoms of the bidentate NHC ligand into a single Cr–N scattering path with a degeneracy (*i.e*., number of backscattering atoms) of *N* = 1.9­(1) and an average Cr–N bond length of 1.957(4) Å (Table 1, Table S1, Figure S13). This simplification is justified by the fact that nitrogen and carbon exhibit similar backscattering amplitudes, making it difficult to distinguish them in EXAFS analysis. The four chloride ligands are reasonably well described using two distinct scattering paths, one corresponding to a single chloride ligand with a degeneracy of *N* = 0.8­(1) and a Cr–Cl bond length of 2.120(7) Å, and a second path for the remaining chloride ligands with a degeneracy of *N* = 2.7­(1) and an average bond length of 2.357(6) Å. The Cr–Cr distance derived from the EXAFS fit is determined to be 3.268(11) Å (Tables 1 and S4). The total number of chromium-coordinated chloride backscatterers is therefore 2.7 + 0.8 = 3.5, which supports the triply bridged structure as the most consistent model for the experimental data since the coordination numbers of each ligand type binding to both metal centers in dimeric complexes are averaged in EXAFS analysis. In contrast, in the doubly bridged model, both chromium centers are bound to only three chloride ligands each. The Cr–Cr bond length, derived from the EXAFS modeling (3.268(11) Å), is very compatible with calculated values for an *M* = 7 triply bridged dimer (3.267 Å), or broken-symmetry triply bridged dimers (3.328 or 3.309 Å, respectively, depending on the functional). Cr–Cr bond lengths calculated for doubly bridged chromium­(III) (*S* = 3/2) dimers are longer by ca. 0.4 Å (regardless of the functional used) and are therefore not compatible with the experimental data, indicating that the doubly bridged isomer does not significantly contribute to the structure of **Cr-NHC-N**. The EXAFS-derived Cr–Cl scattering path (bond length 2.120(7) Å, Table 1) fits well to the corresponding calculated terminal Cr–Cl bond lengths of the octahedrally coordinated Cr-center (2.236–2.246 Å) as well as to the bridging chloride ions attached to the square-pyramidal Cr-center (Figure 4b). The longer Cr–Cl scattering path (average bond length of 2.357(6) Å, *N* = 2.7(1)) fits reasonably well to the longer calculated bridging Cr–Cl bond lengths (2.401–2.668, Table S1). This scattering path is characterized by an elevated Debye–Waller (DW) factor of σ^2^ = 0.0051(7) Å^2^, indicating increased static and/or dynamic disorder (Tables 1 and S4). This is consistent with the bridging chloride donor atoms being located at different distances from both excited chromium centers, yet described by a single average scattering path in the EXAFS analysis. In the triply bridged model, three bridging chlorides are assigned to the octahedrally coordinated chromium center and two to the remaining square-pyramidal one (Figure 4b), as one of the three chlorides is already accounted for by the first scattering path (bond length 2.120(7) Å, *N* = 0.8(1), Table 1). This matches the degeneracy of *N* = 2.7(1) derived for the second Cr–Cl path. Because the respective Cr–Cl distances differ significantly between Cr1 and Cr2, averaging them within a single EXAFS path naturally leads to a higher DW factor.

**Figure 4:**
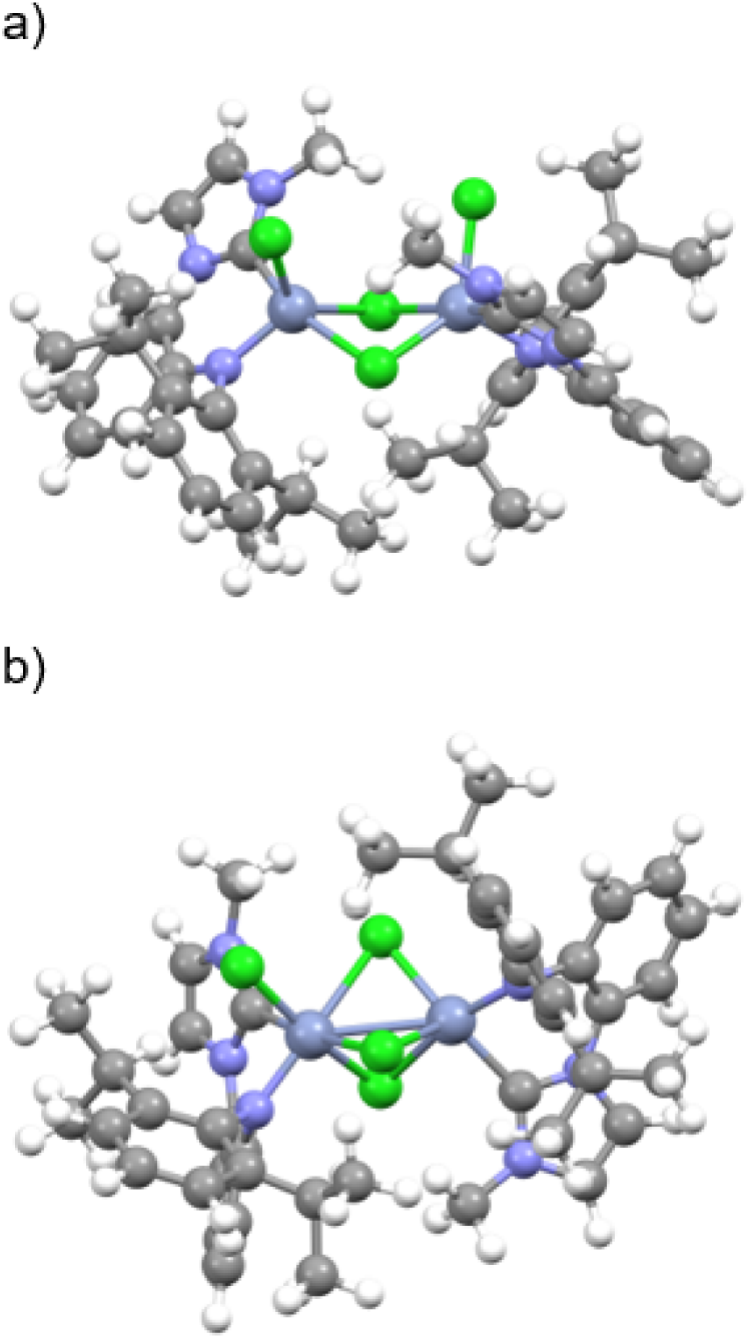
Optimized structural models for **Cr-NHC-N** assuming a) doubly and b) triply chloride-bridged chromium ions.

The XANES pre-edge features are further sensitive probes of the electronic structure and coordination geometry of the investigated compounds. To interpret these features, TD-DFT-based XANES spectra using the TPSSH functional were calculated for both (doubly and triply bridged) geometry-optimized isomers of **Cr-NHC-N** (Figure S12). Irrespective of the bridging geometry, these results unequivocally exclude high-spin (*M* = 7) species (Figure S12b, c). In contrast, the singlet-based TD-DFT-XANES transitions for the BS-optimized doubly and triply bridged isomers give the best agreement with the experimental pre-edge features (Figures 5 and S12d). However, precise quantification of the relative contributions of both isomers in Cr-NHC-N by either EXAFS or TD-DFT-based XANES analysis is not reliable. Based on the EXAFS and XANES data, we conclude that the most likely structure of **Cr-NHC-N** consists of a triply chloride-bridged chromium­(III) dimer with a broken symmetry spin configuration (*S* = 3/2 for each chromium center) as the predominant species, whereas the doubly bridged isomer plays only a minor structural role. Such a triply bridged geometry is highly plausible, given that similar structures have been reported in literature.^47,48^ To shed further light on the electronic structure of **Cr-NHC-N**, complementary EPR and SQUID measurements were performed (see below). Experimental limitations did unfortunately not allow EXAFS/XANES studies of the consequences of the addition of MAO and/or ethylene on the geometric and electronic structure of **Cr-CAAC** and **Cr-NHC-N**, due to the extraordinarily high radiation sensitivity of the formed intermediates.

**Figure 5:**
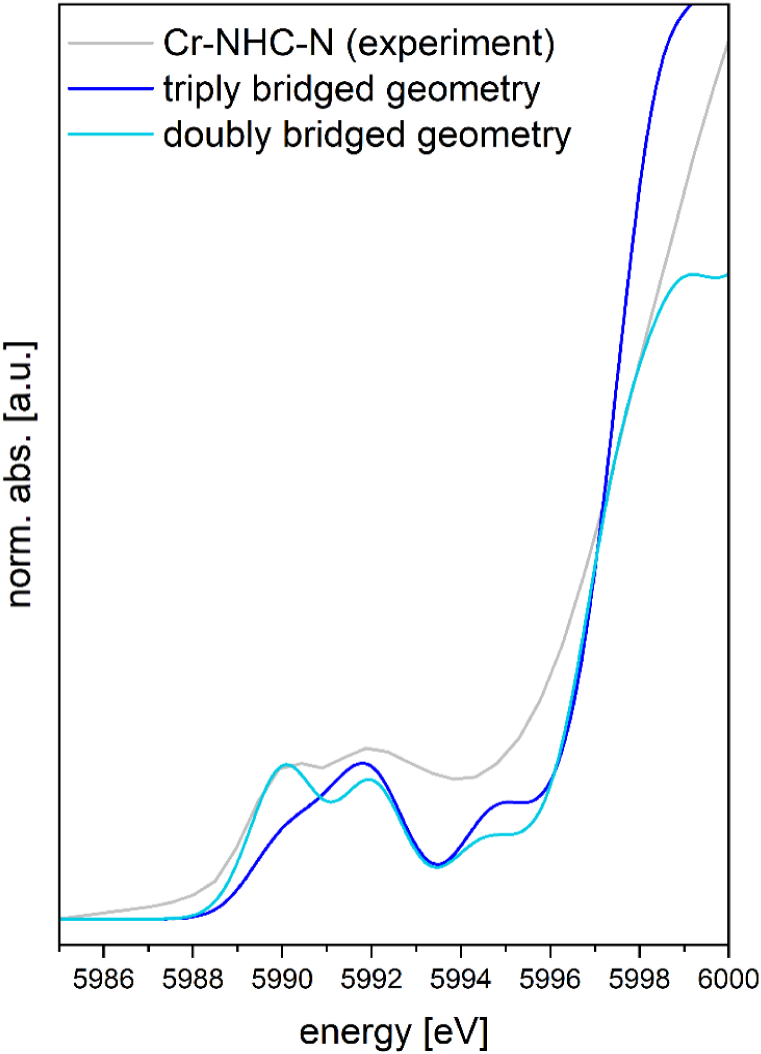
Comparison of the experimental XANES spectrum of **Cr-NHC-N** (gray) with TD-DFT calculated ones for the broken-symmetry DFT-optimized structures (spectra normalized on intensity minimum at 5993.3 eV, energy broadening 1.7 eV), of a triply bridged dimer (dark blue) or a doubly bridged dimer (pale blue).

### HFEPR Investigations

Chromium-based (pre)­catalysts have been studied rather extensively by EPR, especially those based on the Cr-acac + PNP system.^16,17,21,49–51^ The main conclusions of these studies were: (1) A change of oxidation state occurs upon activation with MAO, (2) At the same time, a catalytically inactive low-spin chromium­(I) state is formed, and (3) The intensity of signals due to chromium­(I) is lower in catalytically active mixtures (more ethylene pressure). Therefore, it has been proposed that an unobserved chromium­(II) species is catalytically active. However, these studies could not assess the relevance of chromium­(II) species because chromium­(II) and other integer spin systems have no EPR-allowed transitions at microwave frequencies of about 10 GHz (X-band) at typically available fields of up to 1.5 T.^51^ Measurements at higher microwave frequencies above 100 GHz (HFEPR) allow such measurements. This was impressively shown for the Phillips ethylene polymerization catalyst. In this heterogeneous catalyst consisting of chromium­(VI) ions in porous silica, the latter are reduced by CO gas, giving clean conversion first to chromium­(IV) and then to chromium­(II).^28^ The last species was unambiguously observed by HFEPR. Therefore, we have studied a number of precatalysts by means of EPR at high frequencies (on the order of 300 GHz). To this end, we developed a frozen-solution sample holder (see SI, Section 4). We first determined the electronic structure of the precatalyst in frozen solution, then studied the influence of catalyst activation by reaction with MAO on the EPR spectra, and finally investigated spectra of ethylene-saturated solutions of the precatalysts after activation with MAO. All spectra were simulated by means of a conventional spin Hamiltonian featuring Zeeman and zero-field splitting (ZFS) terms:

$$Ĥ=\mu_{B}B\cdot g\cdot Ŝ+D{Ŝ}_{z}^{2}+E\left({Ŝ}_{x}^{2}-{Ŝ}_{y}^{2}\right).$$

### Cr-acac

This molecule has been studied previously by means of X-band EPR in frozen solution and doped into diamagnetic analogs. There is some variation in the reported zero-field splitting values (*D* ≈ −0.413 to −0.6 cm^–1^, *E* ≈ 0.18 cm^–1^),^52,53^ which is not surprising because *D* ≫ *h*ν, and indeed, usually the sign of *D* cannot be determined in measurements at X-band frequencies. No HFEPR measurements of **Cr-acac** are known to us. HFEPR measurements of **Cr-acac** in frozen toluene solution at 180–360 GHz and 4 K (Figures 6, S16, and S17) show several sharp resonance lines around *g* = 1.99 surrounded by two much broader peaks. At 10 K, the intensity of the sharp central is higher and decreases slightly on heating to 30 K. The intensity of the broad lines at the highest and lowest resonance fields decreases monotonically with increasing temperature. This behavior is consistent with an *S* = 3/2 chromium­(III) species, where the central line is assigned to the *m*_S_ = −1/2 → *m*_S_ = +1/2 transition, the low-field one to the *m*_S_ = −3/2 → *m*_S_ = −1/2 transition (*B*_0_ ∥ *z*), and the high-field one to the *m*_S_ = −3/2 → *m*_S_ = −1/2 transition (*B*_0_ ⊥ *z*). Note that at the fields employed, the *m*_S_ = −1/2 state is an excited state (Figures S14 and S15). Spectral simulation yielded spin Hamiltonian parameters (Table 2) close to those reported for **Cr-acac** from X-band measurements.^52,53^ Note that the HFEPR reported here unequivocally fixes the negative sign of *D*, in contrast to X-band measurements.

**Figure 6:**
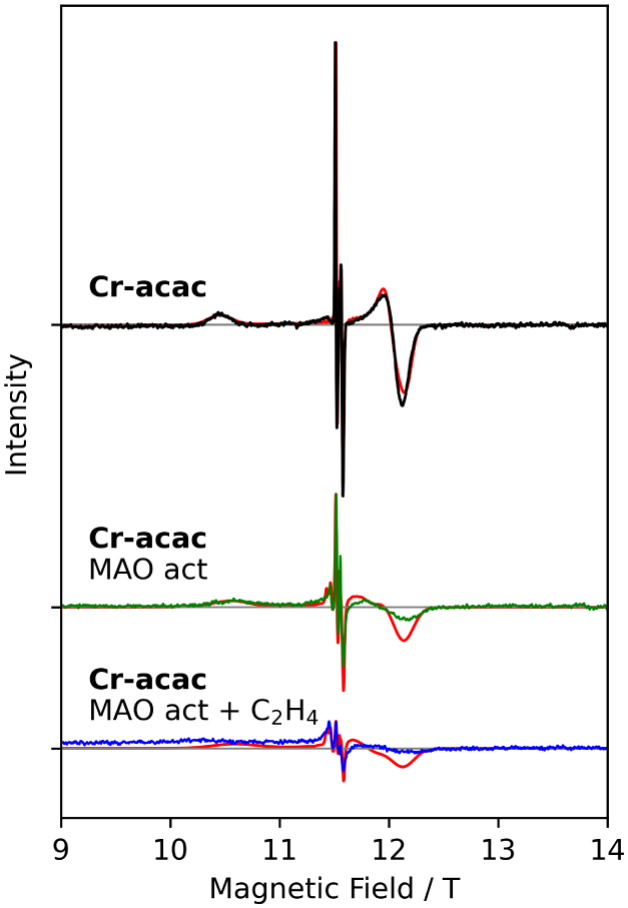
HFEPR measurements of (top) **Cr-acac** in a frozen solution in toluene (black). (Middle) Frozen solution spectrum of **Cr-acac** after activation with MAO (green). (Bottom) Ethylene-saturated frozen solution spectrum of **Cr-acac** after MAO activation (blue). All measurements were taken at 320 GHz and 5 K, and red lines are fits. All spectra have been normalized to the maximum intensity of the **Cr-acac** spectrum.

**Table 2:** Simulation Parameters for the Frozen Solution HFEPR Spectra of **Cr-acac**, **Cr-PNP**, and **Cr-CAAC**

**Parameter**	**Cr-acac (expt.)**	Cr-acac + MAO (expt.)^a^	**Cr-PNP**	**Cr-CAAC (expt.)**	**Cr-CAAC (calc.)**
*S*	3/2	3/2	3/2	2	2
*g*_x_, *g*_y_, *g*_z_	1.9816(2)	1.972(1)	1.990(2)	1.99(1)	1.980
	1.9816(2)	1.998(1)	1.989(2)	2.00(1)	1.997
	1.9800(2)	1.994(1)	1.984(2)	1.998(4)	1.998
*D* (cm^–1^)	–0.52(1)	–0.42(2)	+0.26(1)	–1.695(3)	–2.096
*E* (cm^–1^)	0.022(4)	0.03(1)	0.08(1)	0.032(1)	0.080
*D*-strain (cm^–1^)	0.20(2)	0.14(2)	0.15(2)		
*E*-strain (cm^–1^)	0.00(1)	0.02(1)	0.03(1)		
Linewidth Lorentz (mT)	3.6(4)	10(1)	1.5(3)	0(1)	
Linewidth Gauss (mT)	0(1)	0(1)	15(1)	30(1)	

a This species accounts for 45% of the spectrum, with 55% unreacted **Cr-acac**. After the addition of ethylene, the relative amounts are 90% and 10%.

In chromium-catalyzed ethylene oligomerization, the active species is generated from **Cr-acac** by the addition of methylalumoxane (MAO), typically in the presence of suitable ligands such as PNP (see below). The reaction of **Cr-acac** itself with MAO was previously studied.^16^ Figures 1, S18, and S19 display HFEPR spectra recorded on frozen toluene solutions of **Cr-acac** after the addition of ca. 100 eq. of MAO and flash freezing after ca. 10 min. Interestingly, the spectra are little affected by the MAO addition; the central lines are essentially unchanged, while the peripheral lines broaden. This broadening cannot be reproduced in spectral simulations by incorporating additional *D*-strain, and therefore we conclude that a second species with virtually the same, but slightly smaller, ZFS is formed. Spectral simulation confirmed this hypothesis (Table 1). The average *g*-value increases slightly but significantly upon MAO activation from 1.981 to 1.988, which we tentatively assign to increased covalency around chromium as a result of methylation. Interestingly, no indication of a species with principal *g*-values of 2.0127 and 1.9868, assigned to low-spin [Cr^I^(toluene)_2_]^+^ in literature,^16,53^ or of (high-spin) chromium­(II) species as hypothesized in literature was found. Such species would result in completely different spectra (Figures S14 and S15). The clearly worse signal-to-noise ratio and absolute intensity for the spectrum recorded after MAO activation, in spite of similar chromium concentrations, could indicate that reduction of the chromium ion to chromium­(II) occurs, as previously reported.^16,17^ Although such species can undoubtedly be detected by means of HFEPR,^24,54^ excessive line broadening may preclude resolving the spectra in this particular case.

To further approach catalytically relevant conditions, **Cr-acac** was dissolved in a solution of MAO in toluene that was saturated with ethylene and flash-frozen after ca. 10 min. We note that in the HFEPR spectra recorded on this sample (Figures 6, S19, and S21) the overall shape of the spectra remains similar, but the species formed in the activation step without ethylene appears to be more dominant at this stage. Simulations yielded the spin Hamiltonian parameters reported in Table 2.

### Cr-PNP

This compound has also been studied previously in detail.^16^ **Cr-PNP** is the archetypal catalyst for ethylene oligomerization, selectively yielding 1-octene.^9^ The chemical nature and oxidation state of the central chromium ion of the active species generated have been investigated in literature.^16,17,21^ In particular, X-band EPR measurements showed the formation of a catalytically inactive low-spin chromium­(I) species upon activation in aromatic solvents. Figures 7 and S22 show HFEPR spectra recorded on a frozen toluene/DCM solution of **Cr-PNP** at 275–360 GHz and 4 K. Qualitatively, these spectra are similar to those obtained for **Cr-acac**, and correspondingly, we assign them to a mononuclear chromium­(III) species. The dissociation of the **Cr-PNP** dimer into its monomeric constituents has been reported before.^55^ Spectral fitting yielded a *g*-tensor with principal values of 1.990(2), 1.989(2), and 1.984(2), as well as ZFS values of *D* = +0.26(1) cm^–1^ and *E* = 0.08(1) cm^–1^ (Table 2). Interestingly, no *D*-values have been reported for this catalytically important complex. The positive sign of *D* is surprising at first sight, but the almost complete rhombicity of the *D*-tensor means that the sign of *D* is rather meaningless.

**Figure 7:**
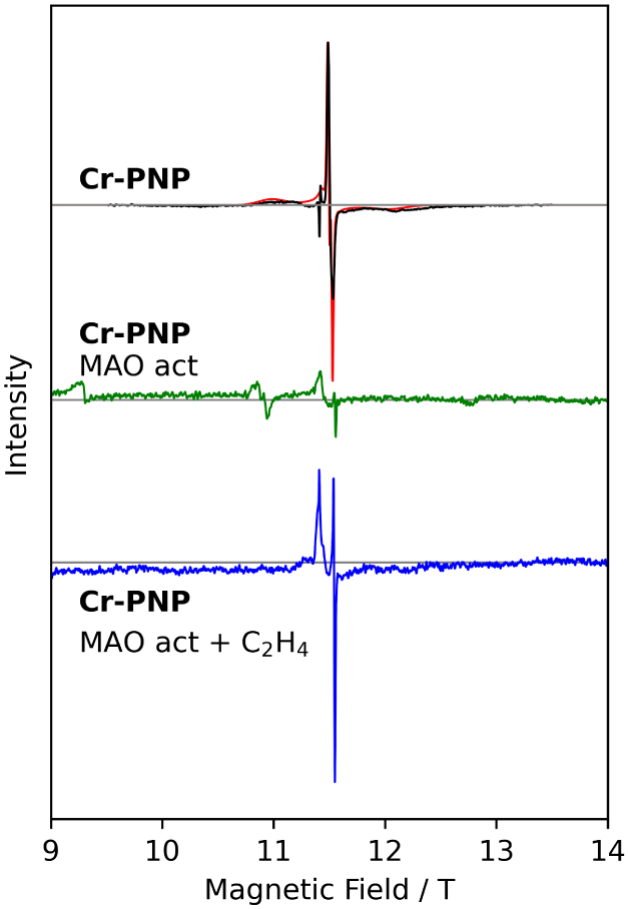
HFEPR measurements of (top) **Cr-PNP** in a frozen solution in toluene/DCM (black). (Middle) Frozen solution spectrum of **Cr-PNP** in toluene after activation with MAO (green). (Bottom) Ethylene-saturated frozen solution spectrum of **Cr-PNP** in toluene after MAO activation (blue). All measurements were taken at 320 GHz and 4 K; red lines are fits.

Second, we recorded HFEPR spectra of a toluene frozen solution of **Cr-PNP** after the addition of ca. 100 eq. of MAO (Figures 7, S23, and S24). At first sight, the spectra look similar to those without MAO, but significant differences can be observed. At *g* ≈ 1.99, there now appears a broad feature with a very narrow feature that is superimposed on it. Second, an additional line is observed at lower fields, i.e., higher effective *g*-values. The separation between this peak and the main line increases with frequency, which shows that it is due to a field-dependent interaction, i.e., a Zeeman splitting. Interestingly, temperature-dependent measurements revealed that the intensity of all features decreases with increasing temperature. This temperature dependence is consistent with a spin *S* = 1/2 species with an axial *g*-tensor, such as that found for low-spin chromium­(I).^16,17,21^ Spectral fitting yielded the following principal values of the *g*-tensor: *g*_∥_ = 2.004, *g*_⊥_ = 1.980. Note that the slight *g*-value anisotropy, which could not have been resolved at the X-band, precludes assignment of this species to an organic radical. Again, no sign of a high-spin chromium­(II) species. The fact that the baselines of the spectra are not straight may be an indication of extraordinarily broadened resonance lines that elude an unambiguous assignment. This would be consistent with the formation of a chromium­(II) species with very strong *D*-strain potentially due to the frozen solution nature of the sample, in contrast to the case for the Phillips ethylene polymerization, which is a heterogeneous solid.^28^ Finally, we have recorded HFEPR spectra on frozen solutions of **Cr-PNP** in ethylene-saturated toluene with 100 eq. of MAO added (Figures 7 and S25). The resulting spectra are very similar to those recorded after MAO addition without ethylene.

### Cr-CAAC

In the foregoing, no clear signatures of the high-spin chromium­(II) species, generally thought to play a decisive role in chromium-catalyzed ethylene oligomerization, were observed. Therefore, we opted to investigate **Cr-CAAC** as a catalytically relevant chromium­(II) (d^4^) complex. In both high- and low-spin states, d^4^ complexes are EPR silent at conventional X-band frequencies,^24,25^ but high-quality spectra have been reported for measurements at higher frequencies. DC magnetometric measurements (Figures S26 and S27) revealed a molar paramagnetic susceptibility-temperature product of *χT* = 2.99 cm^3^ K mol^–1^ at room temperature, consistent with the value expected for a high-spin d^4^ species (*S* = 2) with *g* = 2.0. Below 40 K, the *χT* product decreases slightly, likely due to moderate ZFS (see below), and reaches a value of *χT* = 2.18 cm^3^ K mol^–1^ at the lowest measurement temperature of *T* = 1.8 K. Magnetization measurements (Figure S27) revealed a molecular magnetic moment of 3.7 μ_B_, which is slightly lower than the saturation magnetization of 4 μ_B_ expected for *S* = 2. Interestingly, HFEPR measurements of **Cr-CAAC** (Figures 8, 9, S28, and S29), for both solid-state and toluene-frozen solutions, yielded very rich spectra with an abundance of resonance lines. Extensive spectral simulations gave a consistent set of spin Hamiltonian parameters, including a rhombic *g*-tensor with principal values of 2.00(1), 1.998(4), and 1.99(1) as well as ZFS values of *D* = −1.695(3) cm^–1^ and *E* = 0.032(1) cm^–1^ (Table 2). These values are in line with those reported for other high-spin chromium­(II) complexes^24,54^ and with the chromium­(II) species that is the end point of CO reduction of the Phillips catalyst.^28^ Ab initio CASSCF calculations were carried out to confirm the spin Hamiltonian parameters. Starting from the crystal structure geometry, the calculation yielded a rhombic *g*-tensor with principal values of 1.998, 1.997, and 1.992, as well as ZFS values of *D* = −2.096 cm^–1^, and *E* = 0.080 cm^–1^ (Table 2), in good agreement with the experimental values.

**Figure 8:**
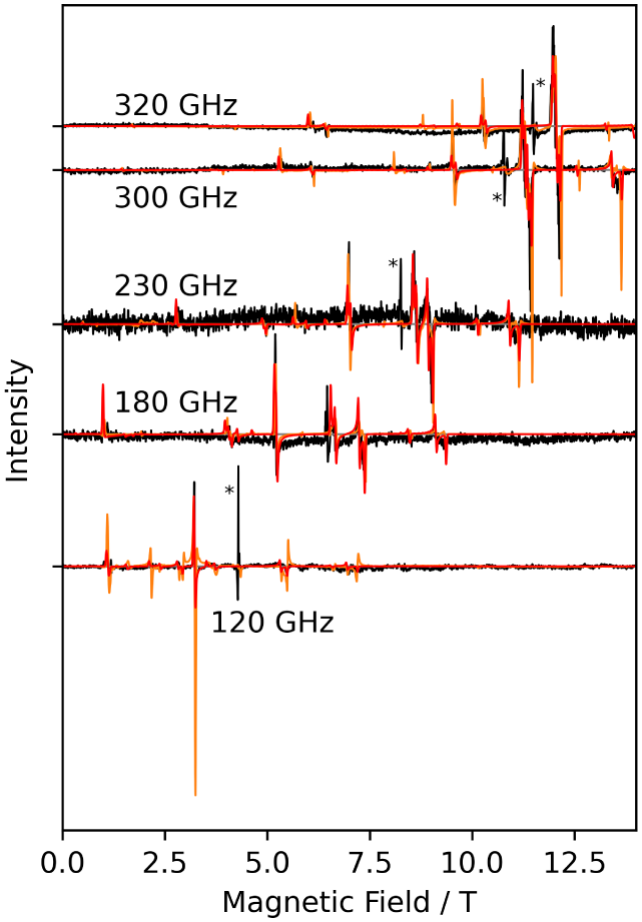
HFEPR measurements of **Cr-CAAC** in the solid state (orange) and in frozen solution (black) measured at different frequencies between 120 and 320 GHz at 10 K. Cr^3+^ impurities in the frozen solution measurements are marked by asterisks and not considered in the simulation. Simulations are shown in red.

**Figure 9:**
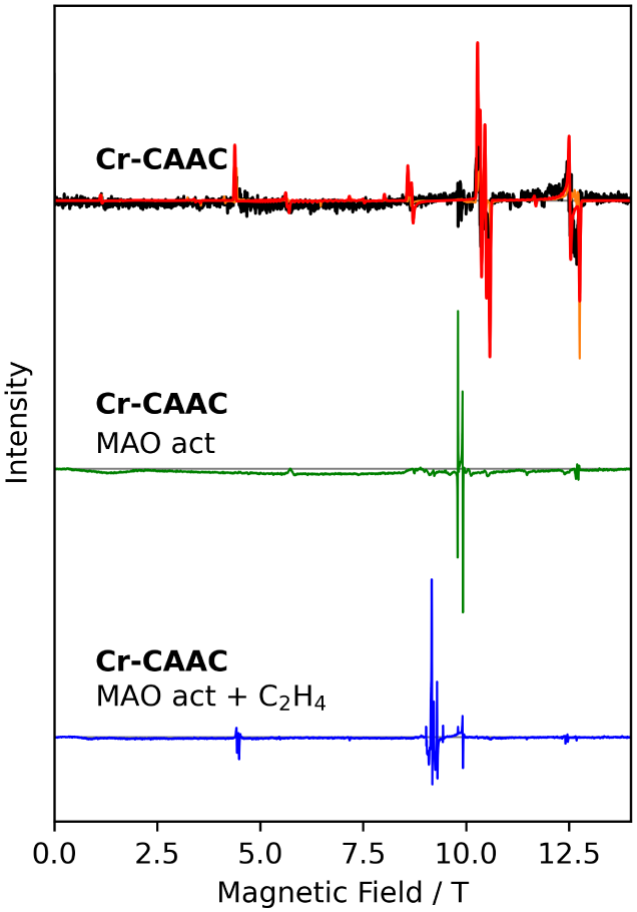
HFEPR measurements of **Cr-CAAC** (top) in the solid state (orange) and in frozen solution in toluene (black), after activation with MAO in frozen solution (green, middle), and in ethylene-saturated solution after MAO activation (blue, bottom). All spectra recorded at 4 K and 275 GHz.

Frozen solution HFEPR spectra, recorded on samples of **Cr-CAAC** in toluene after the addition of MAO (Figure 9), reveal the almost complete disappearance of the resonance lines that were present prior to activation. Instead, two rather narrow, intense resonance lines at *g* = 2.005(1) and *g* = 1.980(1) appear, in addition to a number of weaker resonance lines. Spectra taken at different temperatures (Figures S30 and S31) show that the intensity of the strong resonance lines decrease monotonically with temperature, from which we conclude that they must originate from an *S* = 1/2 species, presumably low-spin chromium­(I), potentially the [Cr­(toluene)_2_]^+^ complex found in EPR measurements on MAO-activated chromium catalysts previously.^17,53^ The weaker but well-defined resonance line follows a different temperature dependence, where with increasing temperature, the intensity first increases, then decreases. These spectra can be described assuming an *S* = 5/2 species, presumably high-spin chromium­(I), with a small ZFS. Satisfactory simulations (Figure S28) could only be obtained in our hands by assuming two of such species, one with positive and one with negative *D*-values (Table S5), in addition to the starting compound. The relative abundances of these three species are ca. one-third each. Note that X-band spectra (Figure S32) do not show the full spectrum, and HFEPR is therefore required to see the whole picture.

HFEPR spectra, recorded on ethylene-saturated frozen solution after MAO activation, are once more completely different (Figures 9 and S33). The intense signals that arose due to MAO activation are essentially absent, while the strongest EPR lines are found in the region of *g*_eff_ = 2.08–2.26 (ca. 9 T) is observed. These lines are due to high-spin chromium­(I) with a positive *D*-value (see Figure S14). The resonance lines at about 4.5 T are assigned to nominally EPR-forbidden transitions within the *S* = 5/2 manifold. The absence of resonance lines at *g* = 2.005 and *g* = 1.980 demonstrates that the low-spin chromium­(I) species, tentatively assigned to the [Cr­(toluene)_2_]^+^ complex, is not formed in the presence of ethylene.

### Cr-NHC-N

Chromium complexes of N-chelating NHC ligands have recently been shown to be active in ethylene oligomerization,^35^ but very little is known about the electronic structure in their native states, as well as changes therein upon MAO activation and reaction with ethylene. XAS measurements shed light on the geometric structure of **Cr-NHC-N**, demonstrating the compound to be a triply chloride-bridged dimer (see above). Magnetometric measurements (Figures S34 and S35) were carried out to investigate the magnetic properties of **Cr-NHC-N**. The room temperature *χT* value of 1.8 cm^3^ K mol^–1^ is consistent with that of chromium­(III) with a *g*-value of 1.98 (*χT* = 1.83 cm^3^ K mol^–1^). Below 200 K, the *χT* value decreases slowly and reaches a value of *χT* = 0.75 cm^3^ K mol^–1^). The molecular magnetization at 1.8 K and 7 T is 1.5 μ_B_, which is clearly smaller than the 2.9 μ_B_ expected for a noninteracting chromium ion, indicating the presence of antiferromagnetic interactions. A joint fit of the magnetometric results gave an exchange coupling value of *J* = 14 cm^–1^ (Ĥ = *J* **Ŝ**_i_·**Ŝ**_j_) and indicated the presence of a monomeric impurity (Table S6).

The HFEPR spectra recorded on solid **Cr-NHC-N** at 4 K display one broad feature centered around *g* = 1.99 with shoulders on both the low-field and high-field sides, as well as additional weaker signals at lower fields (Figures 10 and S36). On increasing the temperature to 10 K, the intensity of the central line increases at the expense of the intensities of the shoulders (Figure S37), indicating that the central line is due to the *m*_S_ = −1/2 → *m*_S_ = +1/2 transition and the shoulders are due to the *m*_S_ = −3/2 → *m*_S_ = −1/2 transition for different field orientations. We attribute this part of the spectrum to the monomeric impurity found in the magnetometric measurements. The additional, weaker peaks are attributed to the *S* = 1, 2, 3 states arising from the exchange coupling in the dimeric main species.^56^ The HFEPR spectra recorded on frozen toluene solutions of **Cr-NHC-N** display a central resonance line at *g* = 1.99 that is substantially narrower than that in the corresponding powder spectrum (Figure 10). No shoulders or additional peaks are resolved potentially because of strong *D*-strain. The poor quality of the spectrum precluded any meaningful fits.

**Figure 10:**
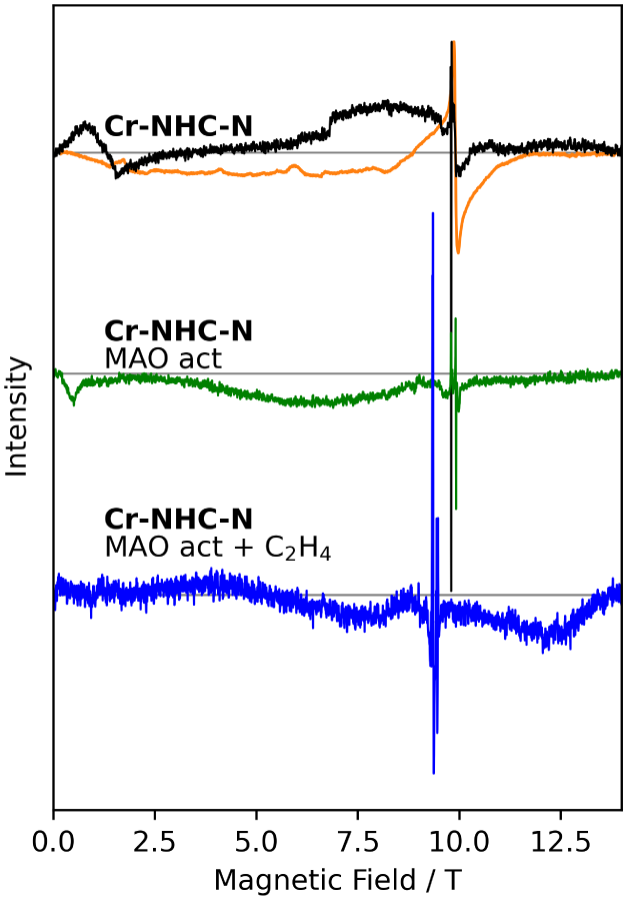
HFEPR measurements at 275 GHz and at 4 K of **Cr-NHC-N** as an inactive catalyst (top) in the solid state (orange) and in frozen solution in toluene (black). The spectrum after activation with MAO in frozen solution is shown in the middle (green), and the active reaction mixture with ethylene in the bottom (blue).

Interestingly, upon addition of MAO, the frozen solution HFEPR spectra at 4 K change substantially (Figures 10 and S38), and a number of narrow resonance lines appear. The lines that are most intense at the lowest temperatures are assigned to low-spin chromium­(I), as for **Cr-PNP** and **Cr-CAAC**. In addition, a number of lines are observed that increase in intensity with temperature, as for chromium­(III). Potentially these latter lines are due to methylated, monomeric chromium­(III) species. As for **Cr-CAAC**, the addition of ethylene shifts the two sharp resonance lines to higher *g*-values (Figures 10, S39, and S40) due to high-spin chromium­(I) (see above).

### Cr-NHC-O

This recently published^36^ compound is closely related to **Cr-NHC-N**, with the difference that the pendant arm of the NHC-ligand features an O-donor rather than an N-donor. The crystal structure revealed two crystallographically different chromium ions, which were assigned +1 (low-spin) and +3 oxidation states, respectively, with a surprisingly weak intramolecular exchange interaction. Solid-state HFEPR data were consistent with this assignment and further revealed a moderate ZFS of *D* = −0.30(1) cm^–1^, *E*/*D* = 0.31(1) for the chromium­(III) center. In frozen solution, the overall spectral shape remains similar (Figures 11, S41, and S42), and identical spin Hamiltonian parameters were obtained.

**Figure 11:**
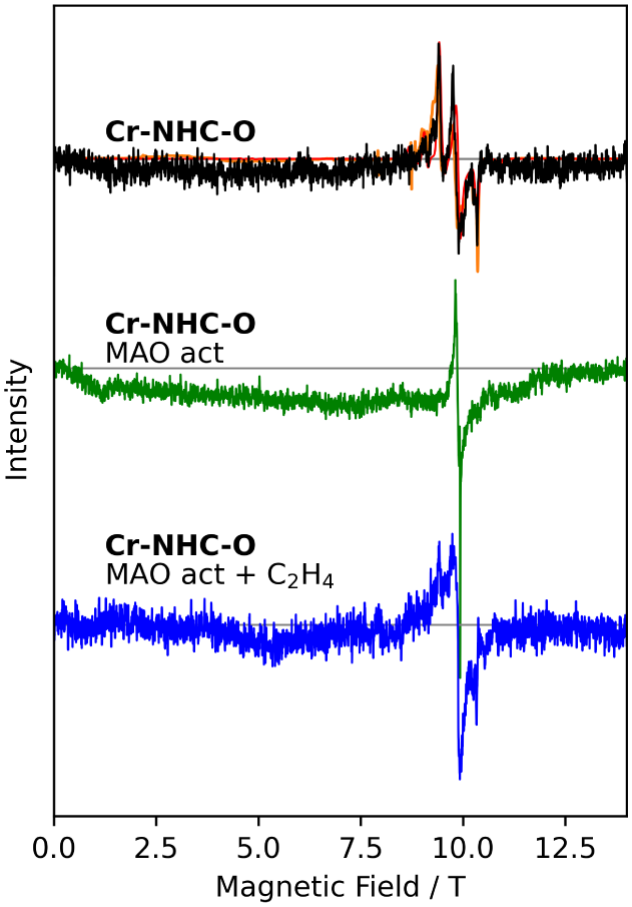
HFEPR measurements at 275 GHz and at 4 K of **Cr-NHC-O** dimer as inactive catalyst (top) in solid state orange) and in frozen solution in toluene (black). The spectrum after activation with MAO in frozen solution is shown in the middle (green), and the active reaction mixture with ethylene in the bottom (blue).

Addition of MAO leads to the disappearance of the outer resonance lines and the appearance of a very sharp resonance line at *g* = 1.980(1) nm and a broader one at *g* = 2.004(1) nm (Figures 11 and S43). Qualitatively, these results are very similar to those found for **Cr-PNP**, but the temperature dependence is somewhat different, because the sharp line decreases strongly with temperature, while the low-field, broader feature appears to show some thermally activated behavior. The latter observation allows an alternative hypothesis, where the broad line is due to a chromium­(III) species, while the narrow one is due to low-spin chromium­(I).

The addition of ethylene and the formation of the active reaction mixture change the spectrum again (Figures 11 and S44). The main signal broadens, and additional features arise that can be assigned to a chromium­(III) species, albeit different from native **Cr-NHC-O** in view of the resonance line positions. No indication of chromium­(I) species remains in these spectra.

## Conclusions

To shed light on the different chromium species occurring in chromium-catalyzed ethylene oligomerization, we investigated a range of chromium complexes. These complexes included the often-used catalyst **Cr-acac**, the best-studied precatalyst **Cr-PNP**, **Cr-CAAC** as a certified chromium­(II) species, as well as the more novel catalysts **Cr-NHC-N** and **Cr-NHC-O**. We have studied some of these by means of XANES/EXAFS and TD-DFT calculations, and all of these with the help of high-frequency EPR. The latter investigations included recording spectra in frozen solution, before and after the addition of methylalumoxane as an activating agent and of ethylene. We have profited from the higher *g*-value resolution of HFEPR to spectrally distinguish different species, from its access to larger energy splittings to unequivocally determine zero-field splitting values, and from the access to small thermal-to-Zeeman energy ratios to determine the spin quantum numbers of the species and the sign of the zero-field splitting. From these investigations, we extract a number of important conclusions: 1. Indeed, MAO activation often yields chromium­(I) species, as hypothesized in the literature. These chromium­(I) species are mostly low-spin but sometimes high-spin. 2. No unambiguous evidence for the generation of chromium­(II) species from chromium­(III) precatalysts, due to MAO activation, was obtained. Frozen solution measurements on a chromium­(II) complex yielded very well-resolved HFEPR spectra, underlining the suitability of HFEPR for studying integer spin species, such as chromium­(II). 3. Saturating the MAO solution with ethylene before the addition of the catalyst resulted in substantially different HFEPR spectra, suggesting that attaining the resting state of the catalyst involves reaction with ethylene. We can also attempt to correlate the HFEPR results with catalytic performance in selective ethylene oligomerization: it appears that redox activity at the metal center is crucial, since neither Cr-acac nor the chromium carbenes display either this redox activity or great selectivity. It must be noted here, though, that a substantial difference between the present studies is that we used an ethylene-saturated solvent rather than 10–45 bar ethylene, which is more common. It has been noted that the preactivated catalyst is unstable without high ethylene pressure.^17^ This study underlines the utility of HFEPR for studying catalytic reactions and its usefulness as part of the experimental palette of X-ray methods, X-band EPR, NMR, and electronic and vibrational spectroscopies.

## Experimental and Computational Methods

No uncommon hazards are noted in the procedures used in this work.

### XANES and EXAFS Measurements and Analysis

Samples were measured at the chromium K-edge (5989 eV) in transmission mode at the P65 beamline (PETRA III, DESY). Measurements were carried out at room temperature and up to 1000 eV above the Cr K-edge. *I*_0_ and *I*_1_ were detected using ionization chambers filled with 1000 mbar of N_2_ and 910 mbar of N_2_ plus 90 mbar of Ar, respectively. For energy selection, a Si(111) double crystal monochromator (DCM) was used with a resolving power of 1–2 · 10^–4^, resulting in an averaged experimental resolution at the Cr K-edge of approximately 1 eV. For energy calibration, a chromium foil was measured simultaneously with the homogeneous Cr samples. The calibration of the absorption edge energy *E*_0_ was performed by identifying the first inflection point in the XANES spectrum of the chromium foil. All samples were measured in the solid state as a self-supporting wafer using boron nitride as a binder. The concentration was calculated as an edge jump of 0.7. Spectra showing the beginning radiation damage were not included in the data analysis. Experimental spectra were analyzed with the aid of the Demeter package.^42^ Background subtraction and normalization of the XAS raw data were performed with Athena software. EXAFS fitting was carried out using the Artemis software, applying the full multiple scattering approach (FMS).^41^ During the multiple-parameter EXAFS fitting procedure, the parameters for each sample were set individually.

### Geometry Optimizations for EXAFS

Geometry optimizations without constraints were carried out with the PbEh-3c composite scheme.^57^ For compound **Cr-CAAC**, the overall multiplicity was set to *M* = 5 according to the SQUID measurements. For the dimeric compound **Cr-NHC-N**, the overall multiplicity for the geometry optimizations with PbEh-3c was set to *M* = 7 (only parallel-oriented 3d electrons at the Cr centers) and *M* = 1 to get a reasonable starting structure for the EXAFS analysis. Broken-symmetry geometry optimizations for **Cr-NHC-N** were performed at the B3LYP (20% HF exchange) and B3LYP/G (15% HF exchange) levels of theory, starting from PbEh-3c preoptimized structures of the doubly and triply bridged isomers. B3LYP and B3LYP/G calculations employed the def2-TZVP basis set in combination with the def2/J auxiliary basis for the RIJCOSX approximation, alongside tight geometry-optimization criteria (tightopt) and very stringent SCF convergence thresholds (verytightSCF). Mulliken population analyses were inspected for all broken-symmetry geometry optimizations to verify an *S* = 3/2 configuration for each Cr­(III) center. When using the B3LYP functional with 20% HF exchange, all Cr­(III) centers in both the doubly and triply bridged isomers converged to *S* = 1/2. Reducing the HF contribution to 15% in B3LYP/G yielded the expected *S* = 3/2 configuration for the Cr­(III) centers in the triply bridged isomer. In contrast, the Cr­(III) centers in the doubly bridged isomer consistently converged to *S* = 1/2 across all calculations. Frequency calculations were performed to verify the absence of imaginary frequencies, ensuring that the optimized structures correspond to real energetic minima.

**TD-DFT-based XANES calculation,** including the determination of orbital energies and the first 500 transitions, was performed using the ORCA quantum chemistry package (version 5.0.4).^58–61^ For this purpose, the TPSSh functional^62^ in combination with the def2-TZVP basis set, def2/J auxiliary basis set, and D4 dispersion correction^63^ were used. XANES transitions were plotted with a constant broadening of 1.7 eV and shifted by around 130.0 eV to align with the experimental spectrum. The analysis of the molecular orbital contributions was conducted using the Löwdin population analysis and extracted from the ORCA output file via MOAnalyzer (version 1.3).^64^ Spatial distributions of orbitals were visualized using IboView (version 20150427).^65^

**SQUID measurements** were performed on a Quantum Design MPMS3 SQUID magnetometer. Measurements were carried out on solid-state powder samples pressed into 5 mm pellets. Susceptibility measurements were performed at an applied field of 1000 Oe in the range from 1.8–60 K and 10000 Oe in the range from 30–300 K.

**HFEPR measurements** were carried out on a home-built instrument as described by Neugebauer et al.^66^ The microwave source is a 9–13 GHz synthesizer coupled to an Amplifier Multiplier Chain and broadband frequency multipliers. In this manner, frequencies between 82.5 and 1100 GHz are accessible. The quasi-optics are set up in a beam splitter setup, and detection was done by a bolometer. The probe is inserted into a 14 T Teslatron superconducting magnet from Oxford Instruments equipped with a variable temperature insert (VTI) that can stabilize temperatures between 4 and 300 K. The sample is placed in a new frozen solution sample holder as described later (SI, Section 4). Because the activated catalyst has limited stability after exposure to ethylene,^16,17^ we have minimized the time between exposure to ethylene and freezing of the sample (5 min).

**X-band EPR measurements** were carried out on a Bruker EMX spectrometer with the following settings: *T* = 7 K, ν = 9.6 GHz, 5 G modulation amplitude, and 5 mW microwave power.

Measurements were taken with a 23 G modulation amplitude at 1.23 kHz frequency, with a magnetic field sweep speed of 0.15 T/min and a lock-in amplifier time constant of 1 s.

**CASSCF calculations** were carried out in ORCA 5.0.3.^58^ The basis set for all calculations was def2-TZVP, and RI-NEVPT2 was added to increase accuracy.^67^ The active space was set to 5 orbitals with the number of d-electrons for the respective oxidation state of chromium. The multiplicity and excited states were derived from Tanabe–Sugano diagrams.^68–70^

Spectral simulations of EPR and SQUID data were done with the EasySpin (6.0.6) toolbox in MATLAB 2022b.^71^

## Supplementary Material



## Notes

Further experimental data, including synthesis and characterization (Section 1); XAS (Section 2); HFEPR and magnetism (Section 3); and frozen solution HFEPR sample holder (Section 4) (PDF)
