# Supplementary material for: Relevant Metal Oxidation States of MAO-Activated Chromium Catalysts for Ethylene Oligomerization
Source: Inorg Chem. 2026 Mar 25;65(13):7309–21. doi: 10.1021/acs.inorgchem.6c00208 (PMC13058880; doi:10.1021/acs.inorgchem.6c00208)
Supplement: Supplementary file 1 [file ic6c00208_si_001.pdf]

Supporting Information

Relevant metal oxidation states of MAO-activated chromium catalysts for ethylene oligomerization

Alexander Allgaier,[1] Felix R. Fischer,[2,] Somnath Bhattacharya,[3] Kevin Balliet,[1] Michael R. Buchmeiser,[3] Matthias Bauer,[2] Joris van Slageren[1,*]*

[1] Institute of Physical Chemistry, University of Stuttgart, Pfaffenwaldring 55, D-70569 Stuttgart, Germany. slageren@ipc.uni-stuttgart.de

[2] Chemistry Department and Center for Sustainable Systems Design (CSSD), Paderborn University, Warburger Str. 100, D-33098 Paderborn, Germany. felix.richard.fischer@uni-paderborn.de

[3] Institute of Polymer Chemistry, University of Stuttgart, Pfaffenwaldring 55, D-70569 Stuttgart, Germany

Contents

1 Synthesis and Characterization 3
2 XAS 4
2.1 xyz-files for the optimized structural models of Cr-CAAC and Cr-NHC-N 6
2.2 TD-DFT based XANES calculation 27
2.3 EXAFS Analysis 30
3 HFEPR and magnetism 32
3.1 HFEPR measurements on Cr-acac 34

3.2 HFEPR measurements on Cr-PNP 40
3.3 SQUID and HFEPR measurements on Cr-CAAC 43
3.4 SQUID and HFEPR measurements on Cr-NHC-N 49
3.5 HFEPR measurements on Cr-NHC-O 55
4 Frozen solution HFEPR Sample holder 58
5 References 59

1 Synthesis and Characterization

Cr-acac ([Cr^{3+}(acac)$_3$], acac$^-$ = acetyl acetonate) was obtained from Sigma-Aldrich.

Cr-PNP ([{Cr^{3+}(PNP)(Cl)$_2$}$_2$(μ-Cl)$_2$], PNP = $Ph_2PN(^iPr)PPh_2$) was prepared according to a published procedure.[1]

Cr-CAAC ([Cr^{2+}(CAAC)$_2$(Cl)$_2$], CAAC = 1-(2,6-diisopropylpheny-1-yl)-3,3,5,5-tetramethyltetrahydropyrrol-2-ylidene) was synthesized following a published procedure.[2]

Cr-NHC-N ([{Cr^{3+}(NHC-N)(Cl)}$_2$(μ-Cl)$_2$}] NHC-N$^-$ = 1-methyl-3-(2-amido-N-(2,6-diisopropylpheny-1-yl)phen-1-ylimidazol-2-ylidene) was prepared as previously reported.[3]

Cr-NHC-O ([{Cr^{+}(MeCN)}(μ-NHC-O)$_2$(μ-Cl){Cr^{3+}(Cl)}, NHC-O$^-$ = 1-(mesityl)-3-(2-O-phenyl)-4,5-dihydroimidazol-2-ylidene) was prepared as previously reported.[4]

All samples were characterized by elemental analyses and/or single-crystal X-ray analysis. **Cr-PNP** and **Cr-NHC-N** were characterized by elemental analyses whereas, **Cr-CAAC** and **Cr-NHC-O** were characterized by elemental analyses along with single-crystal X-ray analysis.

Note: MAO (10 wt % in toluene) was sourced from Sigma-Aldrich, toluene was removed under vacuum at 60 °C, and MAO was obtained as a white semi-crystalline powder and used as a cocatalyst for activation.

2 XAS

Table S1. Calculated bond lengths between Cr centers and donor atoms in first coordination sphere as well as Cr–Cr distances for both geometry-optimized structural models for **Cr-NHC-N**: a) M = 7 (PbEh-3c), b) M = 1 (PbEH-3c), c) broken symmetry approach with two Cr(III) centers (B3LYP), d) broken symmetry approach with two chromium(III) centers each (B3LYP/G with15 % HF exchange).

**Bond lengths / Å** **doubly bridged dimer**		**triply bridged dimer**	
Model a			
$Cr^1–C^1$ = 2.040	$Cr^2–C^2$ = 2.034	$Cr^1–C^1$ = 2.091	$Cr^2–C^2$ = 2.040
$Cr^1–N^1$ = 1.904	$Cr^2–N^2$ = 1.898	$Cr^1–N^1$ = 1.902	$Cr^2–N^2$ = 1.953
$Cr^1–Cl^1$ = 2.246	$Cr^2–Cl^4$ = 2.252	$Cr^1–Cl^1$ = 2.320	$Cr^2–Cl^4$ = 2.236
$Cr^1–Cl^2$ = 2.442	$Cr^2–Cl^2$ = 2.460	$Cr^1–Cl^2$ = 2.417	$Cr^2–Cl^1$= 2.568
$Cr^1–Cl^3$ = 2.436	$Cr^2–Cl^3$ = 2.438	$Cr^1–Cl^3$ = 2.424	$Cr^2–Cl^2$ = 2.454
			$Cr^2–Cl^3$ = 2.576
$Cr^1–Cr^2$ = 3.564		$Cr^1–Cr^2$ = 3.267	
Model b			
$Cr^1–C^1$ = 1.977	$Cr^2–C^2$ = 1.977	$Cr^1–C^1$ = 2.019	$Cr^2–C^2$ = 2.035
$Cr^1–N^1$ = 1.832	$Cr^2–N^2$ = 1.840	$Cr^1–N^1$ = 1.876	$Cr^2–N^2$ = 1.861
$Cr^1–Cl^1$ = 2.218	$Cr^2–Cl^4$ = 2.225	$Cr^1–Cl^1$ = 2.326	$Cr^2–Cl^4$ = 2.239
$Cr^1–Cl^2$ = 2.435	$Cr^2–Cl^2$ = 2.298	$Cr^1–Cl^2$ = 2.306	$Cr^2–Cl^1$= 2.361
$Cr^1–Cl^3$ = 2.288	$Cr^2–Cl^3$ = 2.439	$Cr^1–Cl^3$ = 2.491	$Cr^2–Cl^2$ = 2.504
			$Cr^2–Cl^3$ = 2.286
$Cr^1–Cr^2$ = 3.417		$Cr^1–Cr^2$ = 2.801	
Model c			
$Cr^1–C^1$ = 2.025	$Cr^2–C^2$ = 2.250	$Cr^1–C^1$ = 2.089	$Cr^2–C^2$ = 2.055
$Cr^1–N^1$ = 1.913	$Cr^2–N^2$ = 1.914	$Cr^1–N^1$ = 1.927	$Cr^2–N^2$ = 1.989
$Cr^1–Cl^1$ = 2.250	$Cr^2–Cl^4$ = 2.250	$Cr^1–Cl^1$ = 2.455	$Cr^2–Cl^4$ = 2.246
$Cr^1–Cl^2$ = 2.392	$Cr^2–Cl^2$ = 2.524	$Cr^1–Cl^2$ = 2.303	$Cr^2–Cl^1$= 2.475
$Cr^1–Cl^3$ = 2.518	$Cr^2–Cl^3$ = 2.393	$Cr^1–Cl^3$ = 2.401	$Cr^2–Cl^2$ = 2.668
			$Cr^2–Cl^3$ = 2.657
$Cr^1–Cr^2$ = 3.650		$Cr^1–Cr^2$ = 3.328	
Model d			
$Cr^1–C^1$ = 2.025	$Cr^2–C^2$ = 2.027	$Cr^1–C^1$ = 2.019	$Cr^2–C^2$ = 2.040
$Cr^1–N^1$ = 1.913	$Cr^2–N^2$ = 1.914	$Cr^1–N^1$ = 1.876	$Cr^2–N^2$ = 1.953
$Cr^1–Cl^1$ = 2.250	$Cr^2–Cl^4$ = 2.250	$Cr^1–Cl^1$ = 2.306	$Cr^2–Cl^4$ = 2.239
$Cr^1–Cl^2$ = 2.392	$Cr^2–Cl^2$ = 2.524	$Cr^1–Cl^2$ = 2.321	$Cr^2–Cl^1$= 2.504
$Cr^1–Cl^3$ = 2.518	$Cr^2–Cl^3$ = 2.392	$Cr^1–Cl^3$ = 2.491	$Cr^2–Cl^2$ = 2.361
			$Cr^2–Cl^3$ = 2.286
$Cr^1–Cr^2$ = 3.650		$Cr^1–Cr^2$ = 3.309	

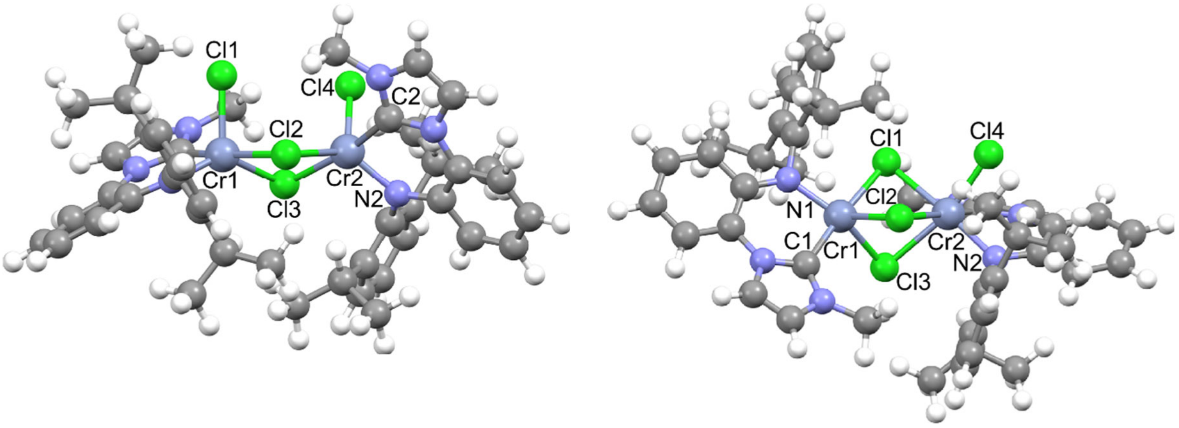

Figure S1. Optimized structural models of **Cr-NHC-N**, assuming two (left), or three (right) bridging chlorides

2.1 xyz-files for the optimized structural models of Cr-CAAC and Cr-NHC-N

Compound Cr-CAAC:

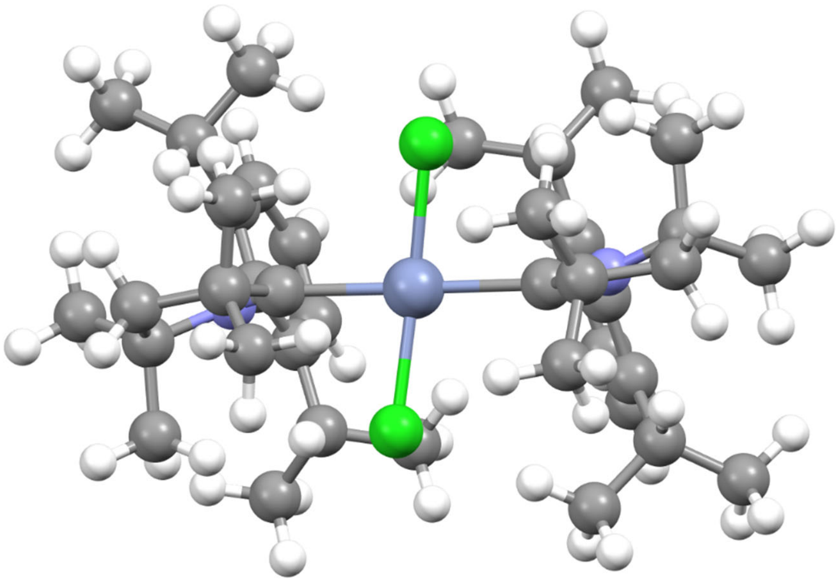

Figure S2. PbEh-3c optimized structure for Cr-CAAC (M = 5).

Cr	5.18821389685628	6.10905927850554	5.81896421588682
Cl	7.00845223094563	6.94310189111321	4.60066702203306
Cl	3.66777391587330	4.80990061790088	7.04529660922383
N	3.81425940721829	6.28946471107487	2.96393590656013
C	4.46821632014600	5.57234704016712	3.83576967342406
N	5.70004536760698	7.37850938252281	8.67985055679767
C	4.03408482930112	5.88074074324267	1.52792948306061
C	4.36139598336708	4.40376644283973	1.71962328814809
H	5.06243482650257	4.04300451779549	0.96594278697653
H	3.45020721808595	3.80675602430411	1.62279414712659
C	4.92134909007951	4.29153615628726	3.15340382176107
C	2.83654090181801	6.13110283518102	0.62959344285619
H	3.09594379392568	5.84105978769504	-0.38917380407377
H	2.55715784114406	7.18481556120519	0.60878614677854
H	1.96502424779357	5.54984477318852	0.91971693921078
C	5.23904663553176	6.64256496249973	0.97666163733460
H	6.10097410379613	6.58791462565602	1.64168694040215
H	5.00980868319819	7.69353465723979	0.81138052123659
H	5.52153387458354	6.21675047973221	0.01297842434455
C	4.23467810689060	3.14084160371590	3.90527820572398
H	4.61127376319019	3.03626050485090	4.92138188319903
H	4.41568692238885	2.20672803622340	3.36926667185399
H	3.15605522054709	3.28226253247862	3.98035349598848
C	6.42832182911065	4.03883378332100	3.18141228183484
H	6.99914641213676	4.80311137984868	2.66004721049231
H	6.63274986827480	3.07373330994131	2.71343969751543
H	6.80614663968265	3.99947633225379	4.20217338351100
C	2.89638624288949	7.34166725520757	3.32088156479270
C	1.55632892317561	6.96534236436222	3.53758486364336
C	0.63855097348891	7.95586340335467	3.86241837379544
H	-0.39322494351529	7.68453616166204	4.04567104567258
C	1.01820018123197	9.27987266007782	3.97162404480282
H	0.28828125501888	10.03623665216229	4.22931525212794
C	2.33496241681058	9.62851289934067	3.75859629276321

H	2.63025853205910	10.66554772694512	3.85302226962469
C	3.29924822923254	8.67905587175669	3.42582730709701
C	1.05004944648673	5.53728107026007	3.47013290053141
H	1.83121635368839	4.90176312669178	3.05240281601012
C	-0.18231117994501	5.39872601194470	2.57309096442777
H	-1.06295834068870	5.85956153133283	3.02207268769327
H	-0.41739937800749	4.34381536361849	2.42511090012234
H	-0.04331742569695	5.85351195523063	1.59212324500213
C	0.73777909719935	5.00274270814162	4.86720754973981
H	1.60983855809451	5.02988972918751	5.51701187534608
H	0.39212578869774	3.96883106442622	4.80796353575908
H	-0.05401733173265	5.58833088951040	5.33863898137977
C	4.71204668732236	9.16904673842708	3.20712064807474
H	5.36477968426311	8.31840898821464	3.02594187604603
C	5.25356073125168	9.86880432194927	4.45211856088693
H	4.72611494299384	10.80019264392202	4.66471365520148
H	6.30961840743459	10.10253865021677	4.31953908248153
H	5.17422513628208	9.23817083069817	5.33603467090327
C	4.78669232557813	10.10808089511246	2.00129310565758
H	4.36187910272949	9.66261825001393	1.10075126538995
H	5.82467273713086	10.36718087642472	1.78795444447158
H	4.24714585346334	11.03941676736703	2.18347689049033
C	6.02244161227283	6.47896759033731	7.79157455841411
C	6.98026325443937	5.49299289591239	8.43994867901216
C	5.98930509255388	6.98056349395680	10.10652399223206
C	6.55272593212608	4.02775626350910	8.38180186790826
H	6.42348505040900	3.68696091028547	7.35497606849624
H	5.61637759892715	3.83662922541482	8.90030021439724
H	7.33314759583537	3.41496098541800	8.83766828114214
C	8.30630658502877	5.63303210158942	7.67446424179358
H	9.07190121408801	5.03781142257547	8.17684644418592
H	8.65379439185978	6.66597169586507	7.63883279781881
H	8.21916666678886	5.28990730540277	6.64523607871832
C	4.76223427448970	6.26705096488852	10.67359652994326
H	4.40264354840396	5.47696313607037	10.01381778343274
H	3.94108512747358	6.95951685548951	10.84645668753113
H	5.01881498096500	5.82035377762733	11.63511472174130
C	5.16165697719713	8.67421245501107	8.34941439797848
C	3.78309937837620	8.90672827712221	8.25596414538839
C	3.35752047340093	10.19783571930551	7.94831088785731
H	2.29661614931690	10.39353158706254	7.86172258771356
C	4.25158652920170	11.22790273510881	7.74889872450679
H	3.89538278185301	12.22209764773834	7.51209119942696
C	5.60719962657986	10.97967064365548	7.84477775133864
H	6.30593454227518	11.78839272636318	7.67311461596566
C	6.09138695117425	9.71246430681181	8.14385240377609
C	2.71523913044695	7.85615645437121	8.46139847567850
H	3.18596010642842	6.88859392178582	8.62113735875192
C	1.84751058861885	8.18466047977776	9.67827106038033
H	2.43943411454087	8.36510445489251	10.57621249883506
H	1.16476284726699	7.35988876213052	9.88752997152493
H	1.23862715955594	9.07539474295578	9.51097158867152
C	1.83559907590942	7.70777894819800	7.22150119763671
H	1.24452163414589	8.60448803842154	7.02766800837315
H	1.14643826325595	6.87401831648251	7.35376453325201
H	2.42195486333537	7.50219766724350	6.32801848950362
C	7.59796304901979	9.53799745640620	8.19548598046199
H	7.83031668077800	8.54673286669884	8.58683419968196
C	8.20588227869491	9.62314359603583	6.79558612877453
H	7.80023844052769	8.86506451592682	6.12870857594054
H	9.28792293356614	9.48686179919915	6.84374739885558

H	8.01601734466747	10.60159236286249	6.35016527258050
C	8.26460975578333	10.56276136047664	9.11738502265343
H	8.22187214787951	11.56998053132379	8.70111103916078
H	9.31976251918627	10.31776702905117	9.24679883782475
H	7.80379466645397	10.59897292025448	10.10449225738209
C	7.14065427163132	6.00514567807323	9.88698054037873
H	8.09587826850422	6.52467925060457	10.00289143812743
H	7.12669288263720	5.19585485093582	10.61817384975622
C	6.34949105635691	8.14549468328851	11.01098075898821
H	5.55696715665145	8.89330685464151	11.05774319452896
H	7.27159352756781	8.63676542721502	10.71192251318473
H	6.50101396461951	7.76597292987965	12.02229288924924

Cr-NHC-N (doubly bridged, M = 7):

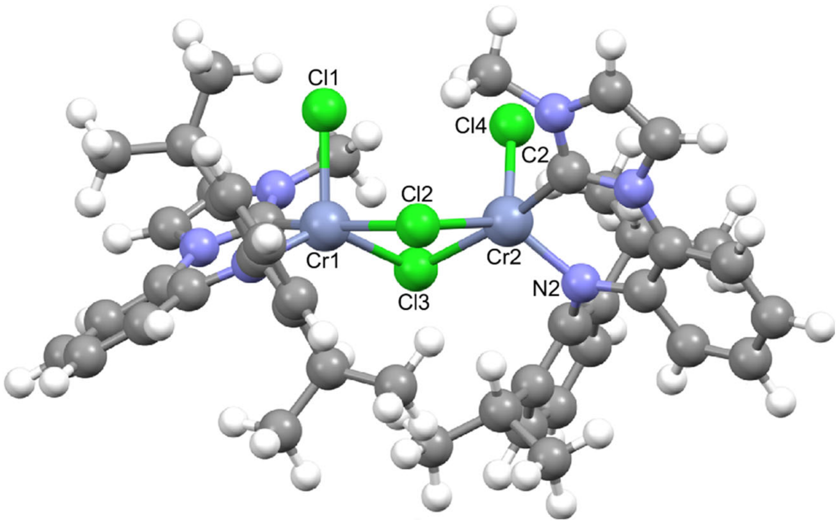

Figure S3. PbEh-3c optimized structure for **Cr-NHC-N** (doubly bridged, M = 7).

Cr	7.45296001646073	1.61144814710844	6.35358147298355
Cl	10.40456527577229	0.65456370377321	3.31124722489715
N	4.57481164874084	2.35475222291453	5.75261137124885
C	5.77274165555616	1.98961780336919	5.26007752555166
N	5.66796493360324	2.09998461702729	3.92598258342951
C	3.72890359409850	2.71931526705569	4.72194356081812
H	2.73762301884598	3.10327474635254	4.86624806692118
N	6.52346555695734	2.11981508952327	7.93553752834935
C	4.42147643346740	2.54794861734696	3.58282270295248
H	4.13131399248408	2.72289185544713	2.56154938545260
C	6.67433314736905	1.78364597673399	2.92469212571303
H	6.52179458942658	2.44062488441255	2.07152708565274
H	6.58183220155174	0.74779352860386	2.60861705159719
H	7.67375707078963	1.94754724943628	3.30869641992961
C	4.21269850522327	2.38110907399773	7.12060355618790
C	5.17814035531293	2.35092291527086	8.14874707347330
C	4.68390146555071	2.54372491869956	9.45601818743957
H	5.38620827309773	2.58585123812891	10.27505721188797
C	3.34178989528171	2.66381666317185	9.73894004050925
H	3.02881086146136	2.79666015942703	10.76591235794022
C	2.40544759060323	2.59710386585304	8.71900854742322
H	1.34555471865253	2.65237651564237	8.92257208763702
C	2.85540445207947	2.45823359260103	7.42385606786819
H	2.12176824816097	2.39298228524775	6.63341765095300
C	7.37696306881767	2.46382712695364	9.03078503713771

C	7.70834896485031	3.81470592033698	9.23303264122991
C	8.48505683086242	4.14914779902660	10.33688724150898
H	8.74421615599594	5.18608471119613	10.51104091145136
C	8.92657460325847	3.18468887800191	11.22078829148558
H	9.52426637713663	3.46335599228369	12.07903332240559
C	8.60558682219518	1.85780683988622	11.00083456433881
H	8.96386271984919	1.11017660214109	11.69563865480061
C	7.83734824918369	1.46588535366273	9.91046669168811
C	7.23058167985124	4.92811827729447	8.32419681196592
H	6.79149394352173	4.48752750058573	7.42970600630198
C	6.15811170924215	5.77635879726711	9.01105956531802
H	5.30013733738695	5.17920050607173	9.31927805897503
H	5.79592030483467	6.55328354059684	8.33510775739032
H	6.55325829600583	6.27323279423862	9.89970358918927
C	8.38871175568466	5.81058500572836	7.85744653107520
H	8.80458621926963	6.40374762445426	8.67412017237549
H	8.04311422261387	6.50773674645171	7.09330822335762
H	9.18829847816376	5.21375858245062	7.42351322876581
C	7.50189103499935	-0.00574291007076	9.75003853813869
H	7.26360585894886	-0.19879850208008	8.70213689007005
C	6.27009906825866	-0.38756539104287	10.57527057634532
H	6.43020140817621	-0.18136119772598	11.63551340583128
H	6.05915699566798	-1.45421401616497	10.47418547207823
H	5.37929191111377	0.15291388354690	10.25941804930637
C	8.67498594588150	-0.91321500206531	10.12331050597639
H	9.59284715813181	-0.61068736433944	9.62262316966973
H	8.46081655870254	-1.94332946320452	9.83494298847779
H	8.85883987131032	-0.92457839294962	11.19904400223977
Cr	10.14025682733251	-0.45463942631042	5.25360553575538
Cl	7.71370646539308	-0.41471043846401	5.02578273846580
N	13.08436947502150	-1.04115590308220	5.72899670675183
C	12.10690488716007	-0.11911080169086	5.64897754061776
N	12.71733808815202	1.07221672299327	5.73427973320045
C	14.31233340472743	-0.41586058165349	5.84514557134110
H	15.25082976120897	-0.93344038010602	5.87781382824753
N	10.51090875256703	-2.31143724425887	5.12512787506143
C	14.06975538567113	0.90609617282276	5.85458517096470
H	14.75081933837162	1.73612143462233	5.92462960983561
C	12.08828885804063	2.38262705144168	5.72536410554253
H	11.80788101853651	2.67820022449077	6.73268602137708
H	12.79834082338956	3.09736870473985	5.31621870638886
H	11.20284972821876	2.38118949033269	5.09980619861480
C	12.90305582286328	-2.44519068523352	5.70018123668711
C	11.66683605630500	-3.03085534494650	5.35188150584677
C	11.65198621656110	-4.44028720540242	5.28446506506692
H	10.73490606283511	-4.93528842085021	5.00288580475983
C	12.74741836856433	-5.21725995020766	5.58497801756075
H	12.66429573328113	-6.29404418145482	5.52287370095418
C	13.93587523037116	-4.62128952598791	5.97568137978683
H	14.80195222867770	-5.21140656687012	6.23938946088978
C	13.99441915711629	-3.24539117853896	6.02997711950405
H	14.91977026460201	-2.79470102858876	6.35595919128535
C	9.43208535115952	-3.05950606359839	4.55937765380141
C	8.40716927537482	-3.53745775459630	5.39645076443713
C	7.41450838317248	-4.32633329738780	4.82857745713072
H	6.61628484709687	-4.71254750809539	5.44856636002183
C	7.41644848721704	-4.62704317802663	3.47800301950270
H	6.63431702170281	-5.24739300068089	3.05970457812271
C	8.41235842511852	-4.12345651132627	2.66518846308727
H	8.40109565106438	-4.35423949909593	1.60700764184697
C	9.43216773099295	-3.33077283995808	3.18226508232204

C	8.35753410144882	-3.24730687570151	6.88440700349517
H	8.91244202251428	-2.32806862107464	7.08290664697408
C	9.02857079971483	-4.36272384972283	7.68868356521492
H	10.07489988924800	-4.49171605450902	7.41547484076922
H	8.99232501691767	-4.13865307844464	8.75670838136470
H	8.52203083688917	-5.31712319446464	7.53152802467222
C	6.93099379674351	-3.02184096908037	7.38558666022667
H	6.35145155620642	-3.94641735328291	7.40836107224915
H	6.94955746036062	-2.63591799593798	8.40517382180639
H	6.39993654379007	-2.30210214132467	6.76485694833579
C	10.49775462026483	-2.81374289459274	2.23752462610263
H	11.13321941067906	-2.10774351459353	2.77232722398235
C	11.38822129704104	-3.94744965109963	1.72627217254511
H	10.81662076495782	-4.67970543273594	1.15225057185205
H	12.16657399625723	-3.55192100952942	1.07139221111289
H	11.87906838644049	-4.47361527261259	2.54441806681848
C	9.87681390082223	-2.04412158612466	1.07157707357674
H	9.22987621999407	-1.24567331388792	1.42992650067447
H	10.65946233184555	-1.58744577244424	0.46443543557259
H	9.29292939450200	-2.69418493835961	0.41744412244165
Cl	9.61502929503971	0.90077209731011	7.23798130806732
Cl	8.43376417998450	3.35995495750559	5.34061309164833

Cr-NHC-N (triply bridged, M = 7):

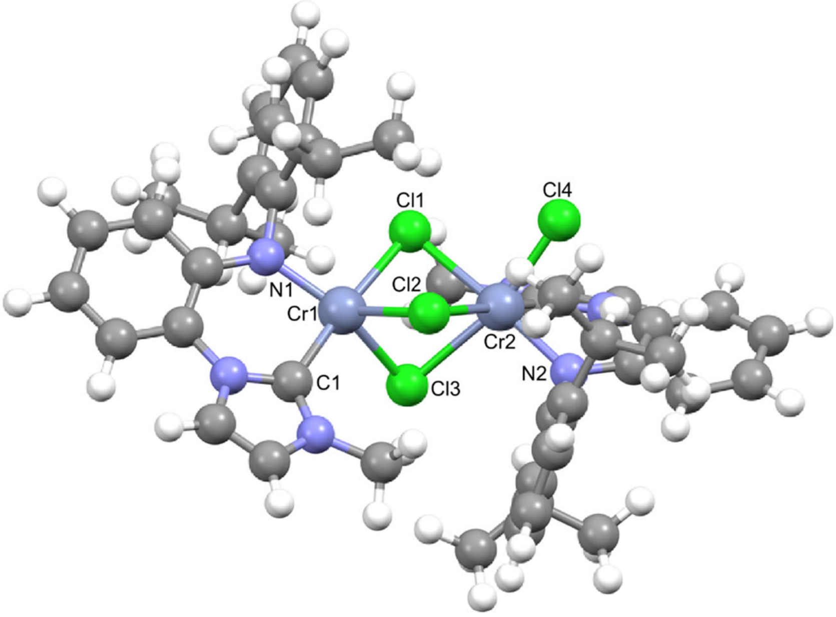

Figure S4. PbEh-3c optimized structure for **Cr-NHC-N** (triply bridged, $M = 7$).

Cr	7.11379796635797	1.14103387544240	6.67911660312010
Cl	8.19077821362238	-0.91270562831757	7.60142641273248
N	4.25854208524988	0.98910989264069	7.43924613769132
C	5.25725789951756	0.31123012104697	6.84683948120958
N	4.72270950450532	-0.87154919306852	6.49062352011022
C	3.10562554003812	0.23485780200556	7.45573327445332
H	2.20014762471839	0.55916114374377	7.93330506034224
N	6.75076578922801	2.02982849431844	8.38032264963010
C	3.40044225023171	-0.92761198633610	6.84952837388247

H	2.78754275183884	-1.79167669695600	6.66221552657164
C	5.36819080327809	-1.97905996125058	5.81407245334558
H	4.80236357240177	-2.88198366261462	6.03237689429371
H	6.37998240818588	-2.10653567775118	6.17809286472631
H	5.39111197241704	-1.81992652705658	4.73964536226062
C	4.35451372504661	2.24525926787577	8.08376253980909
C	5.57172790186433	2.65412256954398	8.66354793321676
C	5.47287909394719	3.72473372182511	9.57514428912139
H	6.34945651459869	4.00349594242345	10.14064778384781
C	4.30440691987780	4.43042677598249	9.76191119294186
H	4.29630748303349	5.25857389092075	10.45875247858554
C	3.15689685313119	4.10176847287953	9.05221708341490
H	2.25064971443302	4.68228634343689	9.14855253414974
C	3.19403289221031	2.99509557466392	8.22863317505486
H	2.30369960212918	2.71113626561492	7.68220322561616
C	7.88884240734091	2.37409619678697	9.15820559901282
C	8.62703597481890	3.54818707494367	8.90023356906893
C	9.80088600252609	3.75723417387440	9.61785295708388
H	10.39799639361948	4.63819011558841	9.41384096445779
C	10.21203462818161	2.88092146730820	10.60414098506824
H	11.12447742542558	3.07260423227890	11.15499637733826
C	9.42635908237822	1.78460536574471	10.91714072782834
H	9.72548695137235	1.13804418625794	11.73313569690659
C	8.26077558511455	1.51290109722134	10.20981741374281
C	8.15412806618195	4.65231803186601	7.96672293054200
H	7.18638433954765	4.37370464421210	7.55067311734939
C	7.96917562129695	5.95626370452050	8.75436350049097
H	7.36776905419266	5.82477975454272	9.65261594876136
H	7.46847714999532	6.69827026597272	8.13107384483257
H	8.92720314390228	6.38145115724205	9.05998889016919
C	9.08263234923144	4.92504214903899	6.78203337234506
H	10.12048265570023	5.05648809246901	7.09330678528075
H	8.77101750509580	5.84233419286966	6.27996321893462
H	9.04152727194242	4.13078048356786	6.04428316558780
C	7.35984245176164	0.37833198073763	10.65258475592351
H	6.75874012695501	0.07270366875035	9.79638377026982
C	6.40027527490265	0.87581675770201	11.73896594690705
H	6.94949953964680	1.21330000323117	12.62031131813294
H	5.72520583536687	0.07658034556011	12.05133078996033
H	5.79069413846088	1.70665719580943	11.38657751827068
C	8.11394397696792	-0.85210640088023	11.15118919134937
H	8.84437964077611	-1.20361009530915	10.42340954589738
H	7.41211387426432	-1.66786317210123	11.33007106055352
H	8.62916782740227	-0.66758166200810	12.09606660212904
Cr	9.70682889674963	-0.42698518574385	5.76247354145312
Cl	7.65757870078592	-0.13505461901959	4.57968649890202
N	11.59104850247354	-2.48286565784036	6.27815860018284
C	11.11246687005155	-1.37255599205166	6.86142915477925
N	11.71121124019313	-1.28721688492776	8.04787418909978
C	12.51132578903192	-3.09863364955583	7.10075749228533
H	13.04468339469060	-3.98906750079419	6.82033281672476
N	11.06287373416488	-0.43679652804486	4.39320053452759
C	12.58272086684614	-2.33906590456387	8.21420104178347
H	13.17913973084922	-2.45996965400624	9.10170234928622
C	11.50259212066866	-0.21609532839506	9.00450675672101
H	10.53827485982796	0.24793842779356	8.83132446606023
H	11.51613787474193	-0.62317837840837	10.01226381402175
H	12.27876020334056	0.53957001452854	8.90374643744324
C	11.30225546037402	-2.80696645798711	4.92961039965902
C	11.15198737710392	-1.75688430596431	3.99234130220018
C	11.07943816965125	-2.13401772527751	2.63947893564707

H	11.00214464943892	-1.35042021489664	1.89764622192046
C	11.11000973025608	-3.45525755680424	2.25404268820897
H	11.04574379278252	-3.70342197459775	1.20294414206489
C	11.19344099500266	-4.47215815380106	3.20369102837612
H	11.18500259012671	-5.50971301351915	2.90079053252006
C	11.28919810327296	-4.14169892267934	4.53862929253002
H	11.36309903161401	-4.92027280345962	5.28798720282762
C	11.87105555822253	0.51177204102718	3.71190576243694
C	11.31910881632108	1.55927051803918	2.94927261828388
C	12.17189288449202	2.37530792025112	2.21210051074600
H	11.74507927974323	3.16797610004081	1.61074579563654
C	13.54034665900969	2.19469790984114	2.21643588186966
H	14.18079863163467	2.83284874285278	1.62164245425924
C	14.08101290510089	1.20115322244466	3.00898438755646
H	15.15696082470222	1.07795009197851	3.04255927083571
C	13.27586432898545	0.37125301666880	3.77880272600134
C	9.83792354733253	1.83250631575529	2.85889104139055
H	9.34078973250960	1.32169569398459	3.67045234401600
C	9.24753062933392	1.28064767818436	1.56322786515411
H	9.70965315162416	1.73941863808242	0.68654452735513
H	9.38362808233798	0.20105259552810	1.48763809738086
H	8.17505566681736	1.47547922302557	1.52201176392956
C	9.49862227681310	3.31203447671402	3.03044044197129
H	9.76769838204748	3.91052045704745	2.15787741795037
H	8.42644697594251	3.42398199411077	3.19175606315494
H	10.00674233944154	3.73287395840849	3.89755089029012
C	13.96643140109206	-0.63187013419117	4.67726369641640
H	13.22189009024694	-1.07074980309266	5.33294922159501
C	14.61770049976504	-1.76280440099430	3.88252993548886
H	15.41131645216139	-1.38250509952792	3.23662608049482
H	15.06684794656276	-2.49856355872611	4.55322601860266
H	13.89718349024822	-2.28170164465473	3.25021424226012
C	14.98585078368538	0.03953695697574	5.60017724159781
H	14.53093023329432	0.85621199903533	6.16085576761079
H	15.38024406448177	-0.68424730928186	6.31633502320463
H	15.83654129136512	0.44703094772493	5.05252543395942
Cl	9.74017735925326	1.68675444147142	6.48221683673057
Cl	6.34156965515480	2.80165915248015	5.39889478826802

Cr-NHC-N (doubly bridged, M = 1):

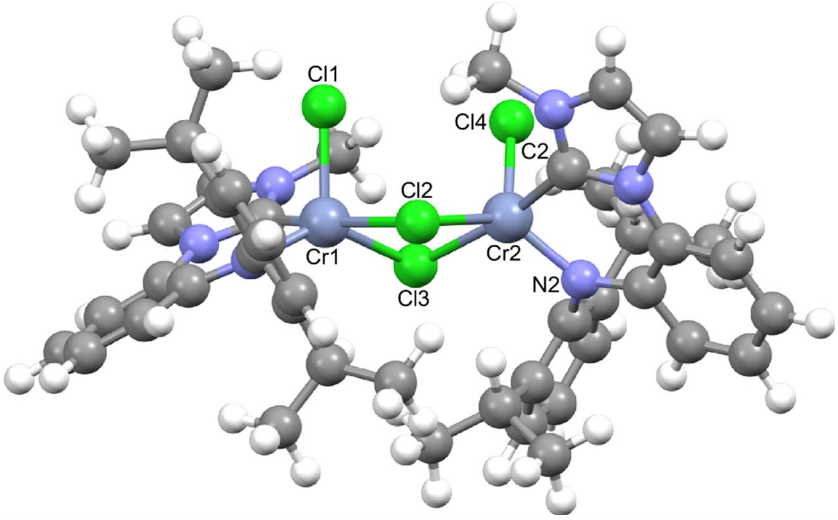

Figure S5. PbEh-3c optimized structure for **Cr-NHC-N** (doubly bridged, *M* = 1).

Cr	7.59975452259319	1.81823036635768	6.29482968416764
Cl	9.95949056481265	0.36922771098444	3.00760159340726
N	4.69791218521360	2.40317412272931	5.75930123667783
C	5.94987720735998	2.21434056382446	5.28063461504078
N	5.82803727325076	2.34460262088026	3.94430777315204
C	3.81619244455325	2.67156699490838	4.72897101294327
H	2.77830497795372	2.90503010894672	4.86644681093262
N	6.67625460510082	2.14964146335522	7.85183478304778
C	4.53462442684586	2.62471965648106	3.59464759177542
H	4.22987329144989	2.78162572012589	2.57470078162869
C	6.87638065477460	2.12770810685509	2.96810560098418
H	6.56851282550828	2.57473708401849	2.02597269786105
H	7.05686293038814	1.06512119500387	2.82245986931948
H	7.79871486345385	2.59567176584192	3.29082392193920
C	4.34063500333212	2.33482241612593	7.11936326416072
C	5.32401523877219	2.27746963333214	8.12697046045757
C	4.85603365226542	2.35827927097858	9.45251721838103
H	5.57265059493946	2.37473372096731	10.25962098169197
C	3.51588140280746	2.40810267823271	9.76955615085208
H	3.21861484778179	2.45774286445135	10.80849964924440
C	2.56436558271825	2.38085947610276	8.76344965335820
H	1.50757225720230	2.38933774227615	8.99003436651827
C	2.98895239291503	2.34869855924032	7.45138385976563
H	2.23826632727452	2.32508487340932	6.67552194928255
C	7.53435986681568	2.39946084967525	8.97559285341173
C	7.85808585104440	3.73144171845433	9.29370093394897
C	8.65758735839079	3.97525803546608	10.40585550673669
H	8.91526250968861	4.99525324448198	10.66241743111374
C	9.11892471819347	2.94372727682830	11.19830394487937
H	9.73537384235131	3.15233661187736	12.06317710234700
C	8.78067285368035	1.64086503458315	10.88457887190278
H	9.13740096763431	0.84040417686255	11.51852497104299
C	7.99005563664478	1.33688480013114	9.78172804370069
C	7.32724306402558	4.92393274544435	8.52452879407746
H	6.86909552760426	4.57322662935193	7.60043603492158
C	6.25176449951212	5.65614455724932	9.33112062627958
H	5.41506342161107	5.00446383102451	9.58058413081402
H	5.85763435963886	6.49742960969920	8.75822494624764
H	6.65603409593171	6.05448637593450	10.26404195739840
C	8.44170690156624	5.89528117657915	8.13398435169344
H	8.85896294419639	6.40778127469060	9.00290347183074
H	8.05004434840209	6.66211424217445	7.46479965051392
H	9.25047014037044	5.38392554030450	7.61660321641276
C	7.61840005126259	-0.11751210848881	9.55225059936651
H	7.33081238036797	-0.24839087403345	8.50772765192120
C	6.42183967855044	-0.52902822445553	10.41695211119116
H	6.62440229541383	-0.35337262186223	11.47558536718289
H	6.21461489061805	-1.59422075743130	10.29563276951354
H	5.51409303169196	0.00927551723560	10.15313564095409
C	8.78526773633640	-1.06543469762811	9.83702499150011
H	9.70120310198064	-0.75111348733892	9.34080298448300
H	8.54392026296969	-2.06900365231644	9.48632669240424
H	8.98934029778134	-1.15099010125081	10.90594116011264
Cr	9.92707323979624	-0.37705864397151	5.09588084063073
Cl	7.50342902493291	-0.24313261760421	5.28429688214242
N	12.85236285497916	-0.81798258504331	5.63407506825889
C	11.84978854922176	0.03071706417218	5.30651809842952
N	12.44371467176833	1.23576440058392	5.19807557585306
C	14.05734541879973	-0.14430067407503	5.70941260722330

H	15.00115920399554	-0.61441609611707	5.90587027900665
N	10.34310669894244	-2.16036606519306	5.15184766237113
C	13.78735916005201	1.14426115691093	5.44211773593066
H	14.44491118408564	1.99471857875972	5.39442672023336
C	11.76917366064003	2.49662854315573	4.96722396664379
H	11.29993419057621	2.85815781588375	5.87920806401290
H	12.50006692606642	3.22439991835112	4.62307836382584
H	11.00582066046498	2.38048676952824	4.20795197630473
C	12.69153169562472	-2.19722250530342	5.86059419667593
C	11.47917276344545	-2.84403230490601	5.55356596910236
C	11.47394288309483	-4.24596788792144	5.67449696392103
H	10.58420315281556	-4.78750083189104	5.39049018800891
C	12.55676044022221	-4.95740142657866	6.14305959844743
H	12.48973235573201	-6.03395561537534	6.22338828784017
C	13.71339881896177	-4.29122399213016	6.51489143359299
H	14.56566567234160	-4.82581331807265	6.91006325654362
C	13.76813685025919	-2.92083459202447	6.36554964751394
H	14.67461016801176	-2.41328600522145	6.65998561027487
C	9.32284411243473	-2.98124935772395	4.56568245898342
C	8.24503554719775	-3.45022525499515	5.34186207090167
C	7.28951679407399	-4.24743414300045	4.72160890525751
H	6.44765901558342	-4.61287066771356	5.29431938134239
C	7.38934726554868	-4.59186542303221	3.38681467310136
H	6.63100954285223	-5.21298271669953	2.92794738531486
C	8.46734437007395	-4.15002247903984	2.64622972393489
H	8.54811673108091	-4.43915016626701	1.60593171691601
C	9.45241323269871	-3.34784153901783	3.21232994533594
C	8.10505746837744	-3.19314098798366	6.83123760316885
H	8.66709070108568	-2.29401068796320	7.08775730766364
C	8.68259486736073	-4.35556979002158	7.64714088294615
H	9.75736924217395	-4.46294419390968	7.51835451124211
H	8.49779315656652	-4.20161572850873	8.71202634350131
H	8.21080637229626	-5.29966008777131	7.36686783462431
C	6.65193476989227	-2.96969981922393	7.25533530801199
H	6.07937072266642	-3.89921834599149	7.25148110964427
H	6.61811604194397	-2.58605567879056	8.27506361455490
H	6.14117379814683	-2.25386482145419	6.61519975127671
C	10.64313168106734	-2.96781084557316	2.35405470497522
H	11.21257068299346	-2.18682937989651	2.85726546888915
C	11.56850661455918	-4.17092839694251	2.15130715925321
H	11.05187056271978	-4.97883523594832	1.62950954593970
H	12.43215866642960	-3.88614638382563	1.54768816226684
H	11.94117475627998	-4.56660136452253	3.09507176081971
C	10.21972671995084	-2.40813016236847	0.99445662083696
H	9.47603190481307	-1.62173064055043	1.10105141277341
H	11.08371750651532	-1.98386724357231	0.48156108981668
H	9.81052012794126	-3.18349450815139	0.34411166924704
Cl	9.68598656472346	0.80022063626358	7.04335957667528
Cl	8.53624350756970	3.66869088960672	5.48924097349657

Cr-NHC-N (triply bridged, M = 1):

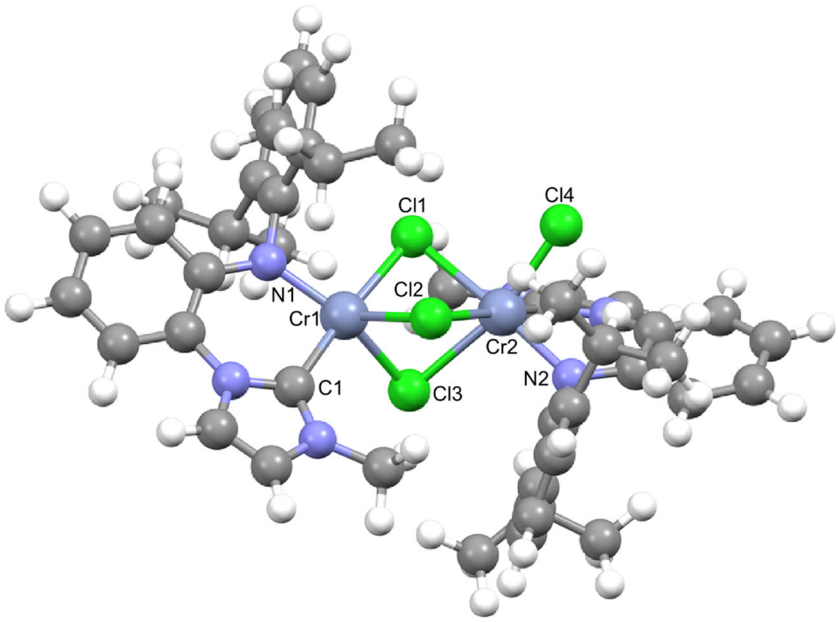

Figure S6. PbEh-3c optimized structure for **CrNHC-N** (triply bridged, $M = 1$).

Cr	6.92039480631366	0.60055929531011	6.63364836998185
Cl	8.99282668206173	1.53472620895567	5.42154676872331
N	4.54904656019657	2.16503285856082	5.76732798532381
C	5.61604560661324	1.48148737222765	5.31786237787823
N	5.54612586752164	1.56265807646513	3.97660673493317
C	3.81874248770211	2.67298923053996	4.71530603485002
H	2.96121657157589	3.30504197917241	4.84976282864142
N	6.54292248082970	2.12342446667244	7.82958908704256
C	4.44456430667920	2.28220257327332	3.59204571303417
H	4.21041282052713	2.47200602965360	2.55936794147081
C	6.46010839043144	0.99339269790759	3.00949220101438
H	6.34572825580164	1.53299811988972	2.07197801308073
H	6.24993066700928	-0.05998256147298	2.84714159812636
H	7.48108238978341	1.10692347469968	3.35274066455672
C	4.24535316085566	2.46651173677246	7.11448556700036
C	5.27984393291661	2.58951537802791	8.06371104583912
C	4.91515385532244	3.25664454839786	9.25219708848134
H	5.68624113154782	3.49747623749257	9.96802252563366
C	3.61193558624755	3.59799422371013	9.54196954217993
H	3.39491574813101	4.08487013446822	10.48407057060522
C	2.58709173012697	3.30421865537661	8.65321421334399
H	1.55488796791886	3.51429518003486	8.89441438761366
C	2.92455488692508	2.75491979413397	7.43309613244151
H	2.14459316700121	2.54037090786839	6.71370779361475
C	7.53027575290021	2.48081063543904	8.78622855385141
C	8.36785265308264	3.57671934640633	8.49762890504625
C	9.33819298060978	3.94556754403025	9.42307739902819
H	9.97883418358867	4.79607264665917	9.22406249381411
C	9.49702026358782	3.24691256845566	10.60672553746926
H	10.26222044139198	3.54042703302990	11.31477901536263
C	8.64871666026163	2.19464941017495	10.89773713053762
H	8.75231923651681	1.68061229452454	11.84594876816981
C	7.63435856570692	1.81069667966113	10.02436860391230
C	8.16211193228009	4.42031441711877	7.25665474722026
H	7.65270168544735	3.80692705504814	6.51379797821567
C	7.25352886025573	5.60997532555121	7.58112371183279

H	6.29113227852633	5.28720429011780	7.97599275189522
H	7.06372260719060	6.20542505258968	6.68581966448865
H	7.71687690476470	6.26240872400033	8.32444351414282
C	9.46426889263772	4.91676952898215	6.63177622709842
H	9.97597199172219	5.64551742731809	7.26328349303197
H	9.25456255578893	5.41294218239238	5.68338357291644
H	10.15509001081558	4.10026283252369	6.42468070699388
C	6.61411816889862	0.79927570372706	10.52742195293360
H	5.83125056572614	0.68277114876220	9.77889325574175
C	5.96217990707199	1.31050716805660	11.82018773617656
C	7.18278068431006	-0.59526798578139	10.79551428066624
H	7.40877696442238	-1.12703539222076	9.87742039564570
H	6.44491673323184	-1.19024299780063	11.33632193567145
H	8.08698390541151	-0.56080179615099	11.40721367353680
Cr	9.77361440712329	-0.73309986446689	5.76766044941658
Cl	7.50509062075252	-1.21156744250364	5.08531931507055
N	12.78416010186330	-0.97276925838345	6.34068543828912
C	11.65749167047666	-0.24680947456161	6.53193191456884
N	11.98494786460174	0.65217338809789	7.47537427304611
C	13.78571558182287	-0.54542537906759	7.18927118354307
H	14.74948194821311	-1.01109809259393	7.26024288236089
N	10.55493028605876	-2.28905387907891	5.00143258576881
C	13.27574082456792	0.47887451464842	7.89088687954294
H	13.71990203255946	1.08590430644699	8.66033224993027
C	11.14077275647678	1.66462960749359	8.08535759853755
H	10.69457931283061	1.29586704092036	9.00462104909749
H	11.75328395125324	2.53649496109680	8.30169037782713
H	10.35202125006584	1.95550530012164	7.40812892528833
C	12.95700612497045	-2.04106592884550	5.43700278152194
C	11.86080913590226	-2.73362958272707	4.88837863272886
C	12.16851760817797	-3.90885976409234	4.17143743216956
H	11.36153845878820	-4.51865060139456	3.79703269937896
C	13.46139186111088	-4.30974488116398	3.91900889917238
H	13.63330111642206	-5.21639211221397	3.35502785525710
C	14.52508900727121	-3.54310393189067	4.36745897023432
H	15.54675951142545	-3.81272400048763	4.14036788692327
C	14.25949946424741	-2.42232159228657	5.12547873741476
H	15.09368084413320	-1.82616482896593	5.46526658287451
C	9.58915679317434	-3.19666808357274	4.44746934983512
C	8.95472787964693	-4.11669009308298	5.28969744356095
C	8.01598211979383	-4.97551456118261	4.72927638527780
H	7.50524544221401	-5.68820464271893	5.36446241239065
C	7.71169916936334	-4.92558244150571	3.38224623876161
H	6.96962630147870	-5.59542401760459	2.96807518950096
C	8.36043834760735	-4.01657723875638	2.56533272012625
H	8.11995298337875	-3.98734453016269	1.50934540662077
C	9.31371664580138	-3.14410897252625	3.07429222575211
C	9.24832675448131	-4.21239638903227	6.77141432760405
H	9.88989903842542	-3.37783816407220	7.05818225467211
C	10.01988117833850	-5.49659737448666	7.08087960430038
H	10.95462128815807	-5.54478816311082	6.52090731230855
H	10.26271909557649	-5.55159868765783	8.14332686009189
H	9.43474713297122	-6.38323361697732	6.82980610115036
C	7.97774102537704	-4.10728033128669	7.61477326985412
H	7.32588154031600	-4.97192107601656	7.47706184077098
H	8.23468832121009	-4.06109363607604	8.67400945250411
H	7.40529352829706	-3.21305039488812	7.37363173821479
C	10.01161828067390	-2.18108856025550	2.13560827756249
H	10.77652871907731	-1.63772880982993	2.69339902453054
C	10.71909859012595	-2.91228887108117	0.99521648488138
H	10.01430825771894	-3.44821696417004	0.35769814277029

H	11.25630403430148	-2.20295025668749	0.36383056407580
H	11.44381117342077	-3.63308297802732	1.37318835539701
C	9.03236324751898	-1.14340375143974	1.59054996836118
H	8.55131193762307	-0.60942797997311	2.40642122223477
H	9.54870684202196	-0.41637924249614	0.96126418762827
H	8.25002823378705	-1.60792966364723	0.98748838675059
Cl	8.84132192219821	-0.55750127454307	7.88466335138950
Cl	5.30777414045907	-0.70826834850406	7.46302941009681
H	5.60610543892336	2.33570501093202	11.73841645038456
H	6.65513880313394	1.26999208114799	12.66339598257115
H	5.10435560609926	0.68670605843738	12.07502326729111

Cr-NHC-N (triply bridged, broken symmetry):

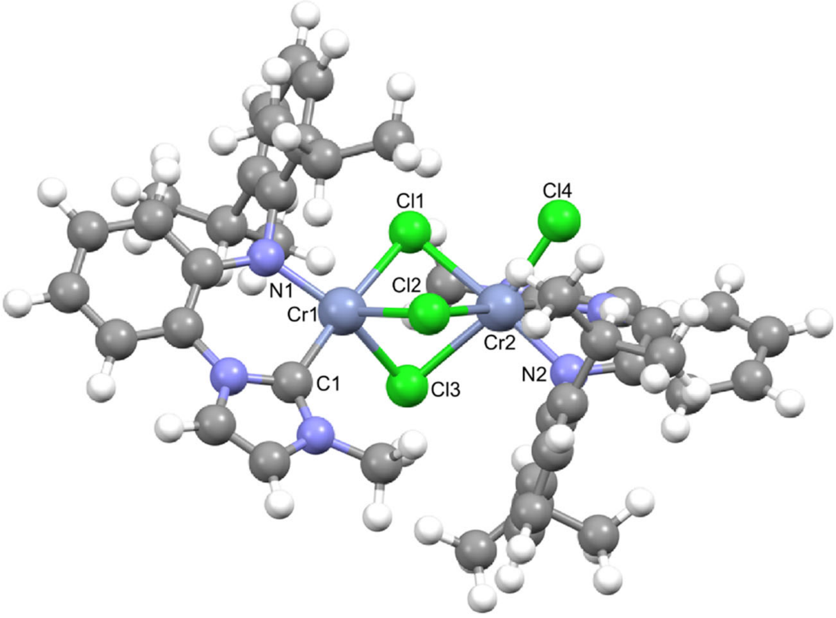

Figure S7. B3LYP optimized structure for **Cr-NHC-N** (triply bridged, broken symmetry).

Cr	0.00000	0.00000	0.00000
Cl	1.50084	1.16347	1.85814
N	1.58170	-0.33368	-2.50563
C	1.61154	-0.52711	-1.16134
N	2.80843	-1.12414	-0.92257
C	2.74407	-0.81261	-3.09347
H	2.95052	-0.71386	-4.14090
N	-0.27248	1.53256	-1.23825
C	3.50299	-1.31083	-2.09800
H	4.47938	-1.76055	-2.12092
C	3.35545	-1.53692	0.36380
H	4.44006	-1.57458	0.27266
H	2.97559	-2.51576	0.64861
H	3.08207	-0.81376	1.12295
C	0.58677	0.37402	-3.23397
C	-0.18767	1.38753	-2.61236
C	-0.85489	2.25447	-3.51282
H	-1.37247	3.11066	-3.11177
C	-0.89232	2.03832	-4.87617
H	-1.44563	2.72641	-5.50374
C	-0.25420	0.93509	-5.43514

H	-0.32239	0.72119	-6.49314
C	0.49501	0.12496	-4.60312
H	1.02109	-0.72333	-5.01943
C	-0.77735	2.81044	-0.80131
C	0.14547	3.87597	-0.64942
C	-0.33033	5.14044	-0.30190
H	0.36890	5.96058	-0.19556
C	-1.68246	5.37361	-0.11309
H	-2.03680	6.36539	0.14251
C	-2.57996	4.32966	-0.26581
H	-3.63622	4.52191	-0.12810
C	-2.16570	3.04051	-0.61019
C	1.63637	3.70998	-0.92561
H	1.84057	2.64110	-0.97042
C	2.01854	4.31966	-2.28696
H	1.43998	3.88400	-3.10135
H	3.07840	4.15050	-2.49497
H	1.84622	5.39927	-2.29228
C	2.52568	4.30070	0.17879
H	2.43630	5.38804	0.23825
H	3.57446	4.07474	-0.02906
H	2.28324	3.88222	1.15538
C	-3.24979	1.97925	-0.81124
H	-2.77376	1.00065	-0.80279
C	-3.97520	2.12196	-2.16434
C	-4.31794	1.99915	0.29783
H	-3.88177	2.01970	1.29443
H	-4.93586	1.10207	0.22239
H	-4.98523	2.85914	0.19830
Cr	-0.00000	-0.00000	3.32799
Cl	0.39826	-1.77428	1.67938
N	-0.56095	1.58747	5.92513
C	-0.37445	1.63706	4.57080
N	-0.65337	2.92466	4.23656
C	-0.97617	2.82609	6.40246
H	-1.22735	3.01850	7.42488
N	-0.03702	-1.13169	4.88690
C	-1.02630	3.65097	5.34230
H	-1.30378	4.68791	5.27841
C	-0.66682	3.54240	2.91189
H	-1.66601	3.50124	2.48521
H	-0.35747	4.58008	3.02091
H	0.02224	3.03104	2.25652
C	-0.39268	0.45358	6.76566
C	-0.21721	-0.85115	6.24504
C	-0.20800	-1.89395	7.20309
H	-0.12562	-2.90880	6.85232
C	-0.28817	-1.67536	8.56280
H	-0.27173	-2.52172	9.23775
C	-0.36888	-0.37873	9.05199
H	-0.39429	-0.17703	10.11437
C	-0.42303	0.66391	8.14790
H	-0.47455	1.66873	8.53554
C	0.16708	-2.54546	4.61674
C	-0.93654	-3.35603	4.28186
C	-0.70688	-4.70921	4.03648
H	-1.53948	-5.34648	3.76680
C	0.56371	-5.25493	4.12013
H	0.71846	-6.30755	3.91674
C	1.63491	-4.44723	4.46735
H	2.62454	-4.88150	4.54059

C	1.46309	-3.08935	4.73098
C	-2.36149	-2.82129	4.19860
H	-2.31591	-1.73509	4.27823
C	-3.22007	-3.33045	5.37026
H	-2.78823	-3.06331	6.33605
H	-4.22280	-2.89913	5.31846
H	-3.32406	-4.41797	5.33769
C	-3.03606	-3.14674	2.85680
H	-3.19168	-4.22134	2.73445
H	-4.01647	-2.66733	2.80983
H	-2.44563	-2.79047	2.01346
C	2.67346	-2.26271	5.15281
H	2.33698	-1.23995	5.32322
C	3.28755	-2.76887	6.46868
H	3.68089	-3.78233	6.36075
H	4.11583	-2.12315	6.77120
H	2.55512	-2.77628	7.27619
C	3.74334	-2.20934	4.05254
H	3.32769	-1.81463	3.12728
H	4.57225	-1.56640	4.35885
H	4.15037	-3.20150	3.84417
Cl	-1.75192	0.54605	1.93708
Cl	-1.38439	-1.37940	-1.10590
H	-3.31475	1.95613	-3.01153
H	-4.42326	3.11472	-2.26556
H	-4.77971	1.38528	-2.22587

Cr-NHC-N (doubly bridged, broken symmetry):

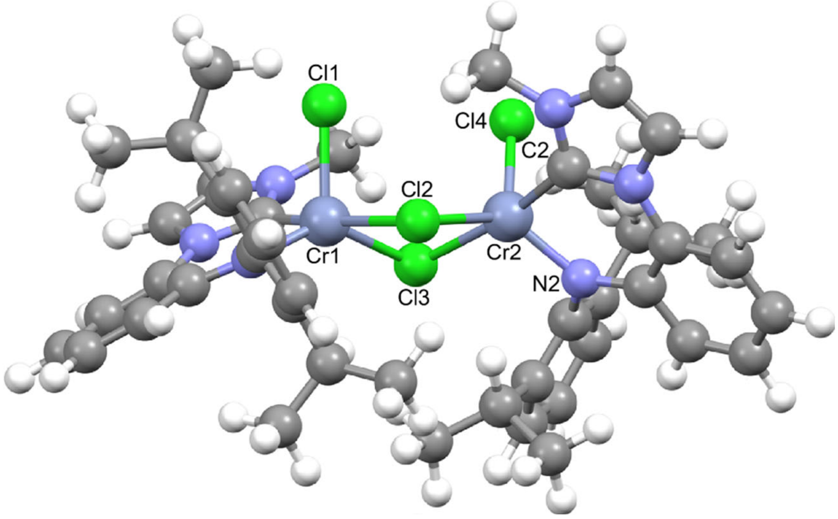

Figure S8. B3LYP optimized structure for **Cr-NHC-N** (doubly bridged, broken symmetry).

Cr	-0.14497774096285	0.09591254269766	-0.07649921237835
Cl	-0.76290509751496	2.14945046831122	3.68922858169777
N	1.32133695732043	1.30061645811554	-2.45605708666629
C	0.75769808063105	1.49404798367400	-1.23059184083071
N	0.87069932261599	2.82720114849224	-0.99625665346934
C	1.75984182333281	2.51359616239724	-2.98161064845734
H	2.18650364570397	2.61741738908213	-3.96064635636525
N	-0.03242176221519	-1.15345527417824	-1.52082714066449

C	1.48095665813335	3.45686216322026	-2.05802237429628
H	1.65158344801996	4.52015043589773	-2.07481640340953
C	0.46835449935336	3.53928065300134	0.21479811333289
H	0.22474332561865	4.56660609228496	-0.05812656822576
H	1.27791161712215	3.52968565493254	0.94510086428693
H	-0.40777878307779	3.06602363715086	0.64873720653429
C	1.44376993862452	0.05003411974784	-3.11867467412634
C	0.71964433313498	-1.09604703902750	-2.69531971233462
C	0.82102418191131	-2.22694695485491	-3.54128603334165
H	0.24362681495279	-3.10393429754080	-3.28985519332654
C	1.62960959335071	-2.26407611002857	-4.66042792264953
H	1.66878831656451	-3.16872109528729	-5.25717878159822
C	2.39662059271043	-1.15621927856521	-5.00313402153919
H	3.06307688694148	-1.17420787375941	-5.85693619612295
C	2.28826826845135	-0.01426556119346	-4.23062095991113
H	2.88828944236729	0.84622465846290	-4.49275443582866
C	-0.91620965143740	-2.29902913851668	-1.41791721466857
C	-2.18189001611754	-2.24860854105512	-2.04904804062481
C	-3.00653842489397	-3.37233399759873	-1.97579256057109
H	-3.98039214392491	-3.34560099692197	-2.45182184833920
C	-2.60467654839422	-4.52320309478578	-1.31850763809781
H	-3.25915856714632	-5.38765294151851	-1.27855028993808
C	-1.35891983811778	-4.56361127231573	-0.71194783288390
H	-1.04957671984923	-5.46886954607110	-0.20290864338598
C	-0.49310870734772	-3.46971491918323	-0.74073043491843
C	-2.67014067074081	-1.04427653991617	-2.84601754129207
H	-1.99695262301706	-0.21072078574782	-2.64397524875800
C	-2.63885952977122	-1.32208469019180	-4.36030799326139
H	-1.63713428938745	-1.58363404485663	-4.70732010258375
H	-2.96632884975452	-0.43517208808809	-4.91179935420551
H	-3.31190600075267	-2.14449671481648	-4.62289713219070
C	-4.07807231822227	-0.59645465020761	-2.42454002353581
H	-4.83520318858075	-1.34160465779051	-2.68668782576355
H	-4.34099561355431	0.33248709702343	-2.93873097084446
H	-4.12888286458354	-0.41006948593832	-1.35107925270363
C	0.87536063362141	-3.61354838009233	-0.07932332379978
H	1.27546325210428	-2.60955855895578	0.07854013596448
C	1.87031488865657	-4.37629494434622	-0.97432117505216
H	1.50169697653024	-5.38417484951285	-1.19117247036713
H	2.83366782528441	-4.47646343621881	-0.46463632423257
H	2.04797125718494	-3.86874230317562	-1.92275924117122
C	0.80237298192598	-4.30700190694302	1.29072556748367
H	0.06021277090407	-3.84187653981900	1.93962393238406
H	1.77449022586468	-4.24830287227468	1.78719859764432
H	0.55917329143140	-5.36921375116753	1.19405418040525
Cr	-0.02894585276527	0.02598155219645	3.57046176787921
Cl	1.44978917645321	0.64482136976638	1.62032869936228
N	-1.40117850411044	-1.23262377866357	5.98195491762994
C	-1.52912598863076	-0.61350497182423	4.77407415539663
N	-2.86726613112431	-0.45727167520920	4.59769754626286
C	-2.65483193006947	-1.43951703709270	6.55235039727205
H	-2.80251986238623	-1.86178791634090	7.52754694647301
N	1.23323269102507	-0.37702257831930	4.95188661909063
C	-3.56089777304724	-0.95908959972740	5.67598879348165
H	-4.63549348720064	-0.91968564826759	5.73686039594382
C	-3.54052345247681	0.12397232736579	3.43744954282372
H	-3.76557748430779	-0.65018685718754	2.70335811982994
H	-4.46405869582666	0.59249677550721	3.77957277979765
H	-2.90539664457170	0.87616669534599	2.97957841845178
C	-0.17368210571529	-1.60108251726009	6.59500806484920
C	1.07508803359883	-1.11218562091548	6.12746758320670

C	2.19574624355964	-1.43976923023735	6.92867182416535
H	3.15880746923933	-1.04601482709593	6.64005708696459
C	2.11731759273812	-2.24828593415555	8.04565952880113
H	3.01909584270124	-2.46815809682379	8.60639398066990
C	0.89456862467319	-2.78573389568306	8.43165113489318
H	0.81410926718495	-3.44896537748551	9.28437279545397
C	-0.23247571381420	-2.45022655232025	7.70381219129753
H	-1.18271562971706	-2.87248527850797	7.99964372517300
C	2.54258517231660	0.22209676086437	4.77575190974597
C	3.54322405403512	-0.46234115423572	4.04234434331930
C	4.80365349848280	0.12679709955055	3.93848766485581
H	5.58234123189491	-0.38431906788165	3.38468658753796
C	5.08547941573988	1.35147957939028	4.52410453050250
H	6.07348042069686	1.78893759399646	4.42559730385165
C	4.09700785254848	2.01203196104976	5.23390571374262
H	4.32149062785278	2.96854999703176	5.69270607540514
C	2.81990163725411	1.46849926607698	5.38409984671944
C	3.32222976614169	-1.82525936575461	3.39223549890604
H	2.24471379556473	-1.97286279471612	3.28879786171355
C	3.86673553148428	-2.97474590812150	4.26147976186183
H	3.38686630981641	-3.01576967673545	5.23957155446947
H	3.69654194910425	-3.93505643131850	3.76480806734083
H	4.94522756089442	-2.86585406199905	4.41591836593867
C	3.93922724614200	-1.91939276319158	1.98770718266625
H	5.03226787649763	-1.93364586160351	2.02602219071128
H	3.62521745293909	-2.85028527608644	1.50878932707396
H	3.62773319260590	-1.08774442220945	1.35525944401443
C	1.80852252458035	2.22402915583880	6.23788411871525
H	0.83551494654179	1.75017681984921	6.10508372520460
C	2.17242315374638	2.14766641962257	7.73197105499176
H	3.13535692044653	2.63012241623341	7.92785882652250
H	1.41424568273851	2.66032751673011	8.33211940444785
H	2.23823420411110	1.11508836302777	8.08086927448595
C	1.65893895406259	3.69076143402051	5.80603654023701
H	1.41360263427100	3.76744335958260	4.74595976638516
H	0.85136278417417	4.16419605611091	6.37177928641902
H	2.57171514455515	4.26376259304578	5.99544424014546
Cl	-1.01022133090405	-1.29598760719995	1.83449937928664
Cl	-2.08452779674990	1.23555913993700	-0.10585834338979

Cr-NHC-N (triply bridged, broken symmetry):

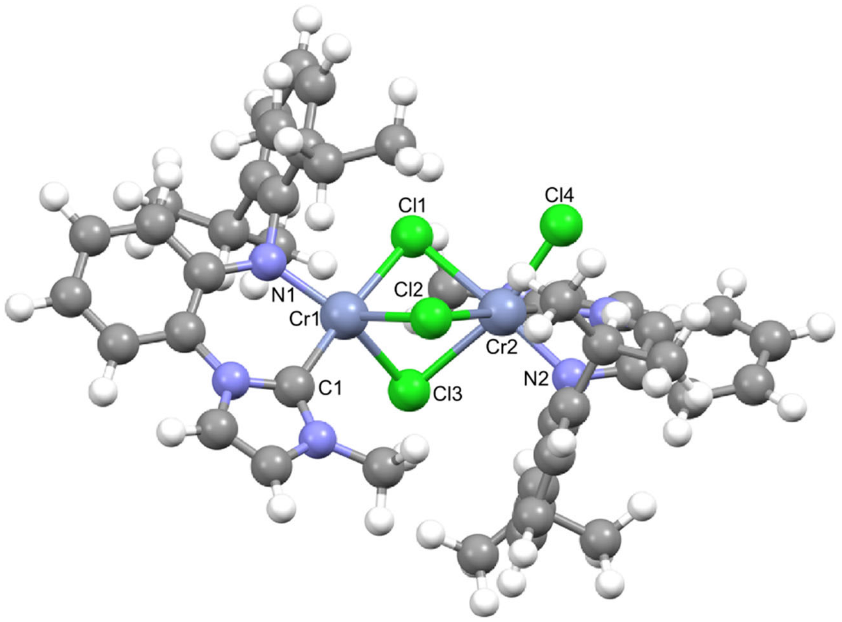

Figure S9. B3LYP/G optimized structure for **Cr-NHC-N** (triply bridged, broken symmetry).

Cr	6.84330681839342	0.56414192816479	6.73114147978931
Cl	9.00881281818254	1.41286578723534	5.50131669137000
N	4.50424847528299	2.21095074828410	5.84880115487339
C	5.57353597161082	1.49973761947785	5.40326923407135
N	5.49314939030794	1.55859244428936	4.04675397776999
C	3.76651489883894	2.69997077636092	4.77923314236245
H	2.91011605502073	3.33522946045056	4.90619597904535
N	6.51218409771383	2.13138355399440	7.96403972558897
C	4.38688631970475	2.28233290152458	3.65613113170710
H	4.15089242083418	2.45383488842532	2.61959494612712
C	6.40693313022515	0.96907720983185	3.07661851542068
H	6.30858560836225	1.52066580689201	2.14097625888004
H	6.17114815788941	-0.08177726019864	2.91144949716824
H	7.42646772964673	1.05310102878470	3.44024393886838
C	4.21211285681102	2.54964528863047	7.19965443637739
C	5.25121243364858	2.65099967559068	8.16411883471458
C	4.89284250431233	3.36173926948337	9.33933904560427
H	5.66484419072171	3.57624299470703	10.06347860706751
C	3.59988951009488	3.76867249322709	9.60698987891686
H	3.39174807784311	4.28317625170464	10.53934811074080
C	2.56963888504007	3.49743267415280	8.70914268847200
H	1.54243441897860	3.75775265700056	8.93310571749491
C	2.89749537807049	2.90526787772195	7.50279282528783
H	2.11879840697442	2.71023923520794	6.77515912162505
C	7.52550016554293	2.62111632016854	8.85936967596692
C	8.21738077557461	3.80746131953505	8.50133488105323
C	9.15573413751267	4.34010231500464	9.38683521094411
H	9.67834749722260	5.25345121048414	9.12305251918811
C	9.41676127878405	3.73712901507134	10.60746019401530
H	10.13969895737328	4.17192769464901	11.29045668521851
C	8.73549652237131	2.58071782800715	10.95484000330273
H	8.93505527262645	2.12200608328903	11.91685593471509
C	7.78538188301472	1.99855833997217	10.11070646977462
C	7.91437167548145	4.56575211251983	7.21407786712373
H	7.34130787678719	3.89449085374988	6.57247695428821
C	7.04445056630511	5.80490766620209	7.49496605079789

H	6.11331626583304	5.53957024407307	7.99889552370409
H	6.79163229073157	6.31297907978505	6.55876401119617
H	7.57866880703346	6.51992147164831	8.12924234046795
C	9.17599735154827	4.97757011625810	6.44148526488610
H	9.77185602057972	5.71277766321695	6.99100552582158
H	8.89503912482946	5.43778531523402	5.48923129072984
H	9.81001477136238	4.11708206677960	6.22006683895640
C	7.04806854409700	0.75926290767059	10.62028868280302
H	6.55479441056158	0.28358380945335	9.77193282564525
C	5.95337341146785	1.10643973385625	11.64930816090656
C	7.98940512398186	-0.27308198941965	11.26711854177341
H	8.85735835290527	-0.49178834990672	10.64546880749785
H	7.44767813119502	-1.20947336165916	11.42626215742630
H	8.34506963965545	0.06411664753013	12.24599167872589
Cr	9.70562049930991	-0.87101375986996	5.89698132970119
Cl	7.41003966035324	-1.29561110080973	5.18325651417424
N	12.75395433574514	-1.09877298626672	6.44024135667639
C	11.60550429223513	-0.38065615679095	6.64199739991513
N	11.95361908900555	0.57263003562434	7.54701945901336
C	13.77978790463788	-0.60356480986325	7.23833937717359
H	14.75939190659991	-1.03661206472384	7.28710375223855
N	10.50990753165291	-2.43240679822783	5.04853285552389
C	13.27032004547692	0.44054867731930	7.91939888449599
H	13.72865819329981	1.09286098944140	8.64344616953562
C	11.11117542763382	1.60043591293685	8.15704082380422
H	10.70116116540949	1.24483029054879	9.10113457534243
H	11.72768349312394	2.48170385426649	8.33271010738468
H	10.29871120675518	1.85730739103021	7.49015504292844
C	12.92573362575456	-2.20774990418194	5.56700246034735
C	11.82688861095751	-2.88347918356120	4.97704825504246
C	12.14860308533682	-4.06556183949093	4.26295264391039
H	11.33924200431495	-4.64244761678358	3.84288065001848
C	13.44240963639035	-4.50470540340892	4.06858166613305
H	13.61593130422528	-5.41557486697856	3.50654912249088
C	14.50750989674365	-3.76890355254777	4.57412923432889
H	15.53375195441627	-4.06908601153030	4.40047973877228
C	14.23376115410947	-2.63595473450191	5.31768699301997
H	15.06640655034781	-2.06307656288734	5.69904801218203
C	9.56470030533721	-3.29480668900344	4.36007369393835
C	8.88155783248038	-4.29578361030783	5.08055225640643
C	7.97705116282807	-5.10194479238900	4.38839015981759
H	7.43273874462170	-5.86968219666751	4.92696765060512
C	7.75061536005971	-4.93646099676531	3.03089099487203
H	7.03568206978982	-5.56962053989813	2.51636911978597
C	8.44486635663989	-3.95858642221503	2.33411679377075
H	8.27098481656571	-3.83976188301902	1.26981009798859
C	9.36657363124383	-3.12949211462156	2.97259841927334
C	9.10477343896955	-4.54014688214120	6.56764399172383
H	9.72119102917818	-3.72451453649782	6.95194573758200
C	9.87722910813368	-5.84975135179097	6.80673640177447
H	10.83934796424264	-5.85384869835628	6.28817868164478
H	10.06822655195760	-5.98747996326580	7.87538895322743
H	9.30323033701623	-6.71351378062640	6.45697016301156
C	7.79407145056490	-4.53163370911729	7.36944195031082
H	7.14728918475038	-5.36971011237806	7.09288156901108
H	8.01158957932478	-4.62456155724520	8.43736778290842
H	7.23550362934010	-3.60656532659055	7.21787276457797
C	10.13066161778421	-2.10016821280598	2.14648879488575
H	10.83558864361754	-1.59565861307854	2.81010105906081
C	10.94747869014623	-2.75294938047456	1.01965316480360
H	10.29919099357724	-3.23826216809324	0.28416585921126

H	11.53493567284178	-1.99524270980967	0.49203328488426
H	11.63861027125235	-3.50525445635601	1.40596385359175
C	9.19769706089203	-1.02281215157808	1.57430742898247
H	8.65702885928059	-0.51575437684451	2.37360459471513
H	9.77222412473900	-0.27565985477141	1.01812854846457
H	8.46160732297648	-1.45679942803016	0.89093609007680
Cl	8.74165137187349	-0.71312706495478	8.00746695438553
Cl	5.16869366524677	-0.67821294718583	7.59010670442610
H	5.15601815869009	1.71232859655817	11.22235889927568
H	6.37606021160358	1.64520798522845	12.50439756203694
H	5.50091079970628	0.18436872223349	12.02649455853900

Cr-NHC-N (doubly bridged, broken symmetry):

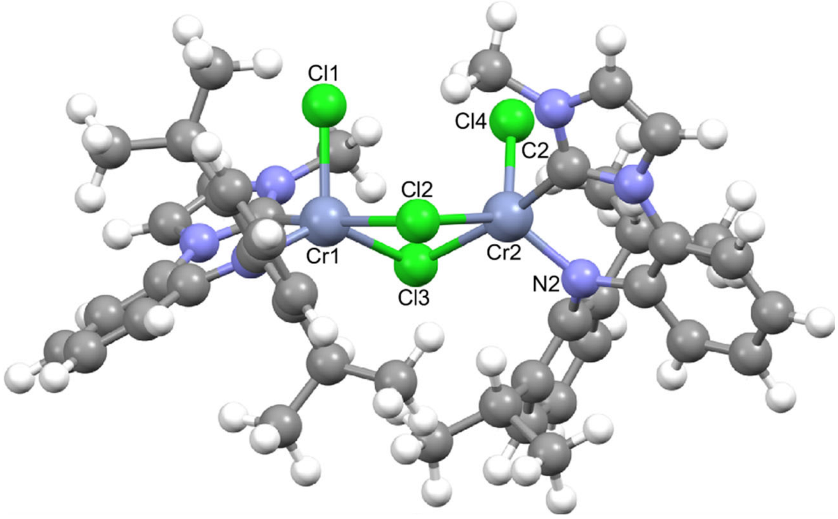

Figure S10. B3LYP optimized structure for **Cr-NHC-N** (doubly bridged, broken symmetry).

Cr	-0.14519275160974	0.09612769485859	-0.07628839126883
Cl	-0.76414795015477	2.14787734622263	3.68757004347155
N	1.32092897855693	1.30113840627838	-2.45578322228537
C	0.75767097575804	1.49425359602282	-1.23010443724382
N	0.87092485933970	2.82730562426912	-0.99535909049462
C	1.75942725624875	2.51423295888743	-2.98107727218868
H	2.18575996974969	2.61834064600570	-3.96022414293328
N	-0.03272154929760	-1.15308728734818	-1.52089238117837
C	1.48093082742134	3.45723756221036	-2.05711088150567
H	1.65166480211853	4.52051143083336	-2.07360052372758
C	0.46880451426215	3.53899876834019	0.21597180268104
H	0.22565346584537	4.56655637822526	-0.05647528988018
H	1.27827362807160	3.52873954491582	0.94636397061568
H	-0.40757426320149	3.06592949369793	0.64963619921883
C	1.44322094457401	0.05069219964012	-3.11872192326092
C	0.71919186037288	-1.09547288733560	-2.69542925480961
C	0.82058323915627	-2.22627252908625	-3.54155944098321
H	0.24330014286980	-3.10332523282584	-3.29010194506364
C	1.62899216495764	-2.26317689216654	-4.66082188268894
H	1.66820478237376	-3.16773378495692	-5.25770298597503
C	2.39581404432032	-1.15518888732072	-5.00354167998447
H	3.06211777236057	-1.17299897840624	-5.85746319153465
C	2.28749914122342	-0.01335782033371	-4.23084030486028
H	2.88738221289973	0.84722521333901	-4.49297168724807
C	-0.91637698283698	-2.29877493401668	-1.41797219048446
C	-2.18206221221568	-2.24859974969553	-2.04911113109843

C	-3.00650452572310	-3.37247688028127	-1.97590872457232
H	-3.98033426920657	-3.34587479567284	-2.45198638870583
C	-2.60442715803391	-4.52330082401218	-1.31867415165414
H	-3.25871004821458	-5.38789988312223	-1.27873074688703
C	-1.35866247696676	-4.56348492392245	-0.71211837288722
H	-1.04916476328538	-5.46873035503791	-0.20315468279616
C	-0.49307305700799	-3.46941892780625	-0.74086409447298
C	-2.67058548495319	-1.04436066598824	-2.84605688766116
H	-1.99745773152366	-0.21071943835025	-2.64417926724586
C	-2.63955168369081	-1.32222735573425	-4.36033673936333
H	-1.63789478022832	-1.58383487304926	-4.70749753202010
H	-2.96705076732754	-0.43532048661312	-4.91181641074489
H	-3.31267626369571	-2.14460983683537	-4.62281671231854
C	-4.07848151806912	-0.59665497047534	-2.42434377916928
H	-4.83558584828014	-1.34194661786848	-2.68615813839824
H	-4.34165428555286	0.33214964525537	-2.93865183184721
H	-4.12905910209785	-0.41006285299664	-1.35090926937093
C	0.87543460528144	-3.61299545244307	-0.07950980345033
H	1.27535511702722	-2.60892632769150	0.07831434170409
C	1.87052603263557	-4.37554571373748	-0.97451335290149
H	1.50218197983485	-5.38356197385808	-1.19120179969483
H	2.83399639875588	-4.47535762141944	-0.46499355128054
H	2.04786486194053	-3.86804357264450	-1.92304114679049
C	0.80258817538660	-4.30638179410534	1.29057100889103
H	0.06052880568047	-3.84116077312023	1.93952221427144
H	1.77477051284334	-4.24769995785748	1.78691225201140
H	0.55930884924597	-5.36858721110893	1.19405609886152
Cr	-0.02925612456711	0.02477485815355	3.57068249040694
Cl	1.45013055482036	0.64324614295527	1.62023836755539
N	-1.40047351448746	-1.23363275886569	5.98267045071516
C	-1.52895305257235	-0.61480550665775	4.77466690111044
N	-2.86720604059245	-0.45898860291285	4.59867682639885
C	-2.65389949022999	-1.44078196701350	6.55346224411360
H	-2.80115204001085	-1.86281596569601	7.52882446594148
N	1.23342521253838	-0.37747662895685	4.95189515124041
C	-3.56035710718535	-0.96081237037020	5.67727557705375
H	-4.63494643592311	-0.92171685679999	5.73842559093815
C	-3.54116764270047	0.12191697386206	3.43866187184839
H	-3.76723924545155	-0.65255869774437	2.70521735142614
H	-4.46418795239000	0.59109286948999	3.78128895919333
H	-2.90610480761728	0.87354850910606	2.97980956576971
C	-0.17273992611886	-1.60153300929438	6.59559789093142
C	1.07576507366161	-1.11224727170942	6.12778707070612
C	2.19663052073261	-1.43896964545161	6.92906785695549
H	3.15944913721137	-1.04476079085878	6.64027513161086
C	2.11865875707347	-2.24718370740653	8.04629572135069
H	3.02054732529925	-2.46641085687176	8.60710143117032
C	0.89620804966482	-2.78519805007180	8.43245568588852
H	0.81613855495891	-3.44828756029303	9.28532162000415
C	-0.23104897634857	-2.45044567682278	7.70460603260106
H	-1.18105972366327	-2.87310207013080	8.00059512663532
C	2.54264222367908	0.22187766678643	4.77528592469089
C	3.54352851502127	-0.46275812814952	4.04242477326678
C	4.80384947998506	0.12658109761781	3.93841759073766
H	5.58272334801166	-0.38471415835071	3.38504818967585
C	5.08533451596751	1.35166970218875	4.52333871720914
H	6.07325874297034	1.78927105132077	4.42472512021860
C	4.09658808640632	2.01246422886950	5.23254209592583
H	4.32076393336738	2.96931737131661	5.69078335175924
C	2.81958940506147	1.46872490066702	5.38285690564818
C	3.32283587369556	-1.82598381515189	3.39288609480099

H	2.24534620903698	-1.97381514281189	3.28948294488755
C	3.86747972041965	-2.97509689809338	4.26252195860715
H	3.38799595980026	-3.01547742768336	5.24082723035642
H	3.69690295734169	-3.93564349148765	3.76645074219718
H	4.94605299111755	-2.86632302498768	4.41648622629118
C	3.93988192562461	-1.92050966963184	1.98839975347139
H	5.03292277502437	-1.93447330962603	2.02676455321860
H	3.62613213989173	-2.85167182228090	1.50984976493361
H	3.62819353008003	-1.08918322385623	1.35562976573300
C	1.80789383509910	2.22458037372943	6.23598692984891
H	0.83502623255628	1.75038901190851	6.10339796900628
C	2.17159608861994	2.14930705558058	7.73016898411780
H	3.13435711098253	2.63216255842263	7.92591004355146
H	1.41318470146584	2.66208932203262	8.32991162433568
H	2.23768165836649	1.11695485259193	8.07969283058387
C	1.65794507510672	3.69095257302613	5.80306507955917
H	1.41258880966492	3.76675298178892	4.74292712797365
H	0.85024148276262	4.16463849787612	6.36841249589168
H	2.57055502970362	4.26436342049472	5.99201442809344
Cl	-1.01133497464766	-1.29567732341052	1.83434095956688
Cl	-2.08466993622386	1.23574596990682	-0.10641085451998

2.2 TD-DFT based XANES calculation

Table S2. Analysis of atomic contributions to selected acceptor orbitals of TD-DFT calculated XANES transitions for compound **Cr-CAAC**.

**Transition (energy)**	**Acceptor orbital / %**								
	No.	Cr center			NHC ligands		chloride ligands		
		d	p	s	p	s	d	p	s
**a** (5992.7 eV)	196	2.1	37.8	0.2	16.5	16.1	1.5	4.0	0.0
**b** (5993.4 eV)	197	8.9	1.0	0.0	23.2	53.4	0.2	0.4	0.0
	198	3.5	17.4	0.2	25.4	41.2	0.5	1.7	0.0

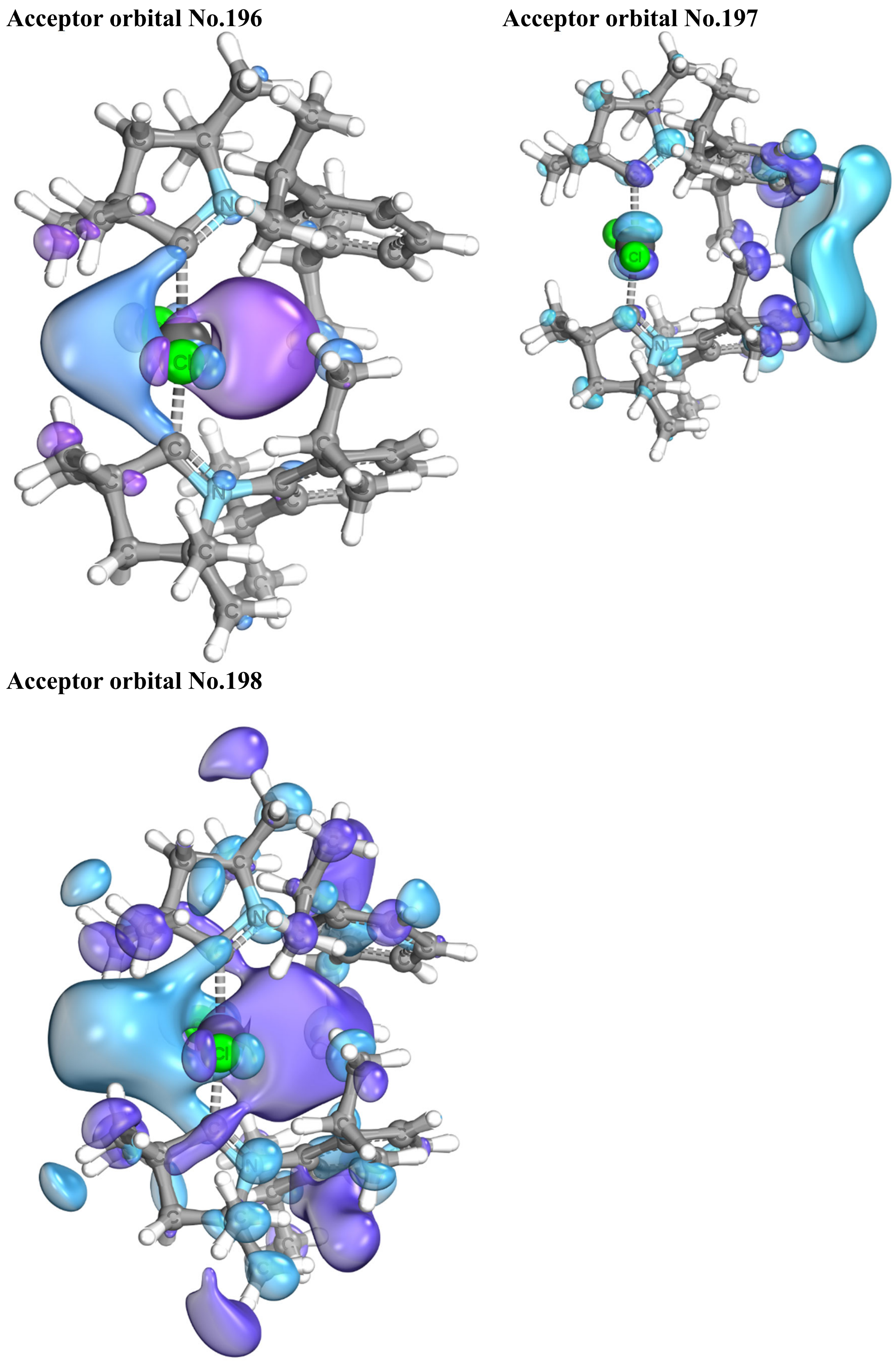

Figure S11. Spatial distributions of acceptor orbitals 196, 197 and 198.

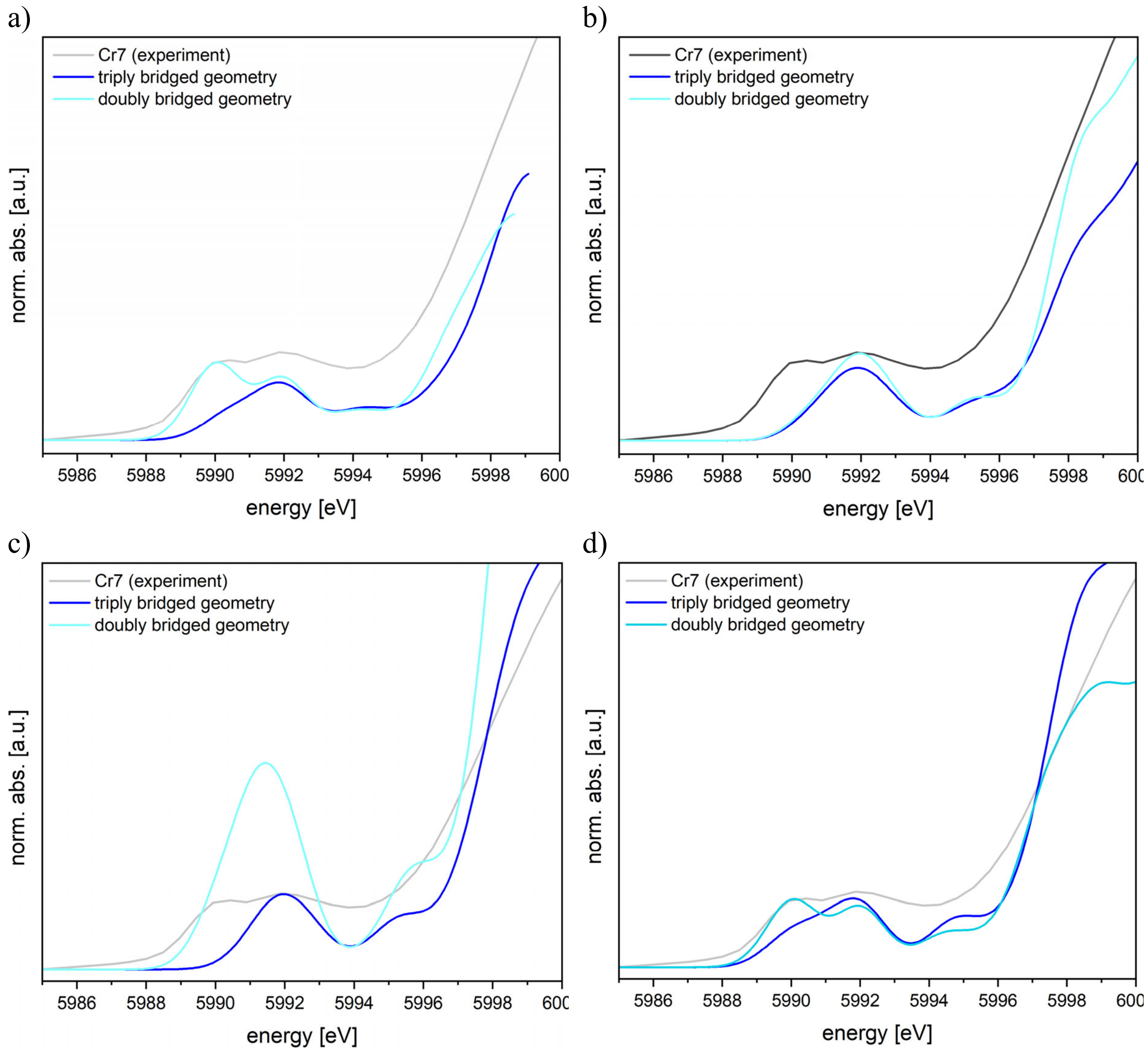

Figure S12. TDDFT-XANES calculations (TPSSH) for a) the PbEH-3c-optimized structures of doubly and triply bridged isomer with M = 1, b) the PbEH-3c-optimized structures of doubly and triply bridged isomer with M = 7, c) the B3LYP/G optimized structures of doubly and triply bridged isomer with broken symmetry approach and M = 7, d) the B3LYP/G optimized structures of doubly and triply bridged isomer with broken symmetry approach and M = 1.

2.3 EXAFS Analysis

The *k*- and *R*-ranges of the EXAFS-analysis of compounds **Cr-CAAC** and **Cr-NHC-N** together with the respective values for *R*-factors, reduced χ^2, delE_0 and S_0^2-parameters are collected in **Table S3**. *del*E_0 describes the energy offset of the theoretical absorption edge energy (E_0) based on the fitted EXAFS data compared to the experimental E_0 value. S_0^2 represents the amplitude reducing factor that reduces the EXAFS signal intensity due to many body effects, such as shake-on/off processes in the absorbing atom. **Figure S13** illustrates the fitted function, experimental data, residual plot as well as first shell contributions for **Cr-CAAC** and **Cr-NHC-N** in *R*- and *k*-space. **Table S3** shows coordination numbers (*N*), bond lengths ($R + \Delta R$) and Debye-Waller factors (σ^2) for all scattering paths of **Cr-CAAC** and **Cr-NHC-N** obtained by EXAFS fitting with the Artemis program.

Table S3. *k*- and *R*-ranges as well as corresponding fit parameters of the analysis of the compounds **Cr-CAAC** and **Cr-NHC-N**.

sample	*k*-range [Å^{-1}]	*R*-range [Å]	*R*-factor	reduced χ^2	S_0^2-value	delE_0 [eV]
**Cr-CAAC**	2.4 – 12.7	1.17 – 4.00	0.0013	60	1.0	-3.85(92)
**Cr-NHC-N**	1.1 – 12.2	1.15 – 4.00	0.0013	48	1.0	-1.47(39)

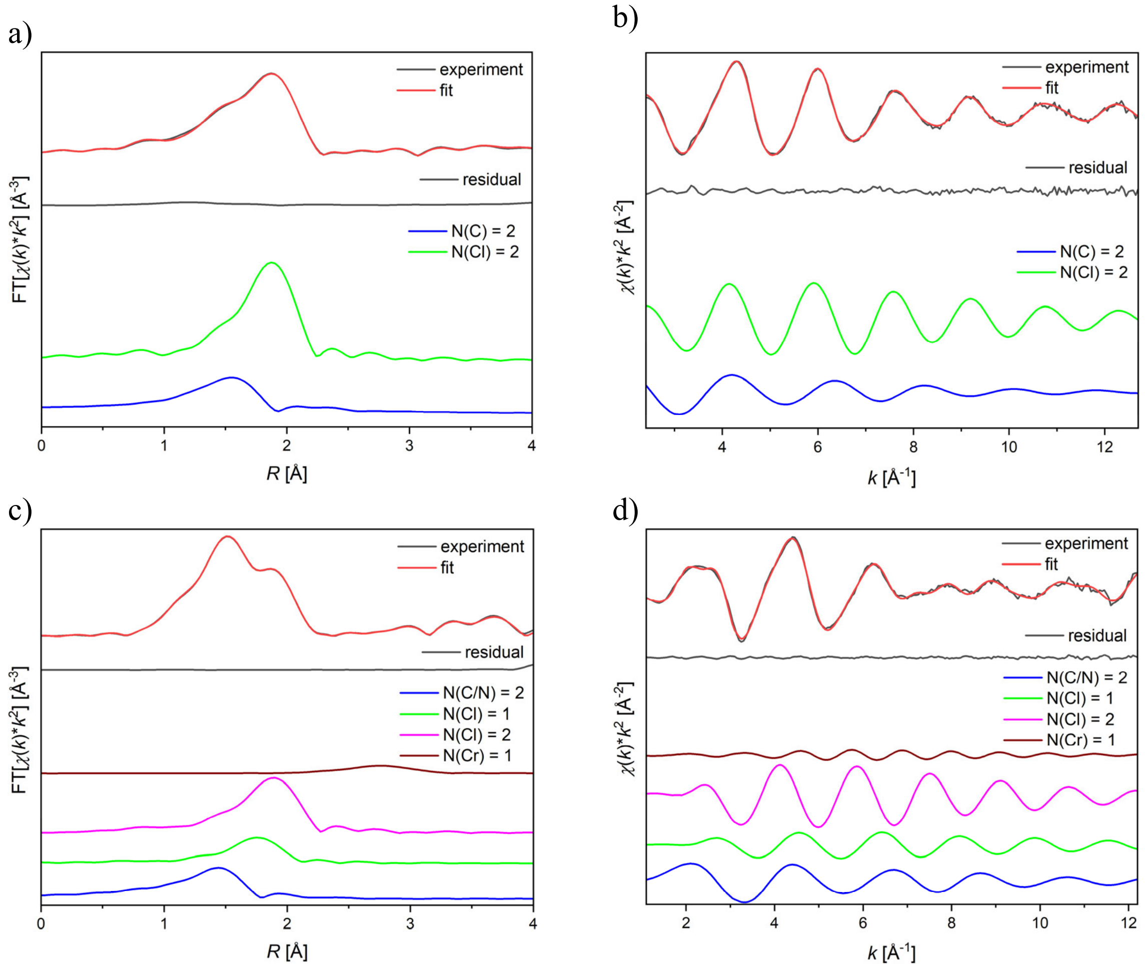

Figure S13. Fitted function compared with experimental data, residual plot and first coordination shell paths for **Cr-CAAC** and **Cr-NHC-N** in the *k*- and *R*-space: a) *R*-space of **Cr-CAAC**, b) *k*-space of **Cr-CAAC**, c) *R*-space of **Cr-NHC-N**, d) *k*-space of **Cr-NHC-N.**

Table S4. Coordination numbers (*N*), bond lengths ($R + \Delta R$) and Debye-Waller factors (σ^2) for all scattering paths of **Cr-CAAC** and **Cr-NHC-N** obtained by EXAFS fitting with the Artemis program. Cr–CC-, Cr–CN-, Cr–NC-, Cr–Cr^2Cl^2-, Cr–Cl^2C-, Cr–CO- and Cr–ClCl-scattering paths represent multiple scattering phenomena.

**scattering paths**	**N**	$R + \Delta R$ [Å]	σ^2 [Å^2]
**Cr-CAAC**			
Cr–C	2.2(1)	2.064(11)	0.0069(7)
Cr–Cl	2.1(1)	2.333(5)	0.0042(2)
Cr–N	2.3(4)	2.994(21)	0.0042(16)
Cr–C	9.0(7)	3.205(17)	0.0109(11)
Cr–C	6.1(9)	3.496(28)	0.0115(12)
Cr–C	4.8(9)	4.582(32)	0.0141(14)
Cr–NC	9.4(17)	3.172(38)	0.0077(20)
Cr–CC	27.9(44)	3.526(35)	0.0120(10)
Cr–ClCl	4.9(5)	4.736(21)	0.0084(4)
**Cr-NHC-N**			
Cr–N	1.9(1)	1.957(4)	0.0036(4)
Cr–Cl^1	0.8(1)	2.120(7)	0.0028(4)
Cr–Cl^2	2.7(1)	2.357(6)	0.0051(7)
Cr–C	2.8(3)	2.437(18)	0.0035(10)
Cr–N	1.8(1)	3.001(12)	0.0057(7)
Cr–Cr^2	1.0(1)	3.268(11)	0.0075(10)
Cr–C	2.8(2)	3.840(17)	0.0048(8)
Cr–C	4.0(2)	4.047(13)	0.0053(8)
Cr–CN	7.3(5)	3.337(12)	0.0054(11)
Cr–Cl^2C	11.0(3)	4.416(12)	0.0042(11)

3 HFEPR and magnetism

Explanation of typical HFEPR spectra for different species.

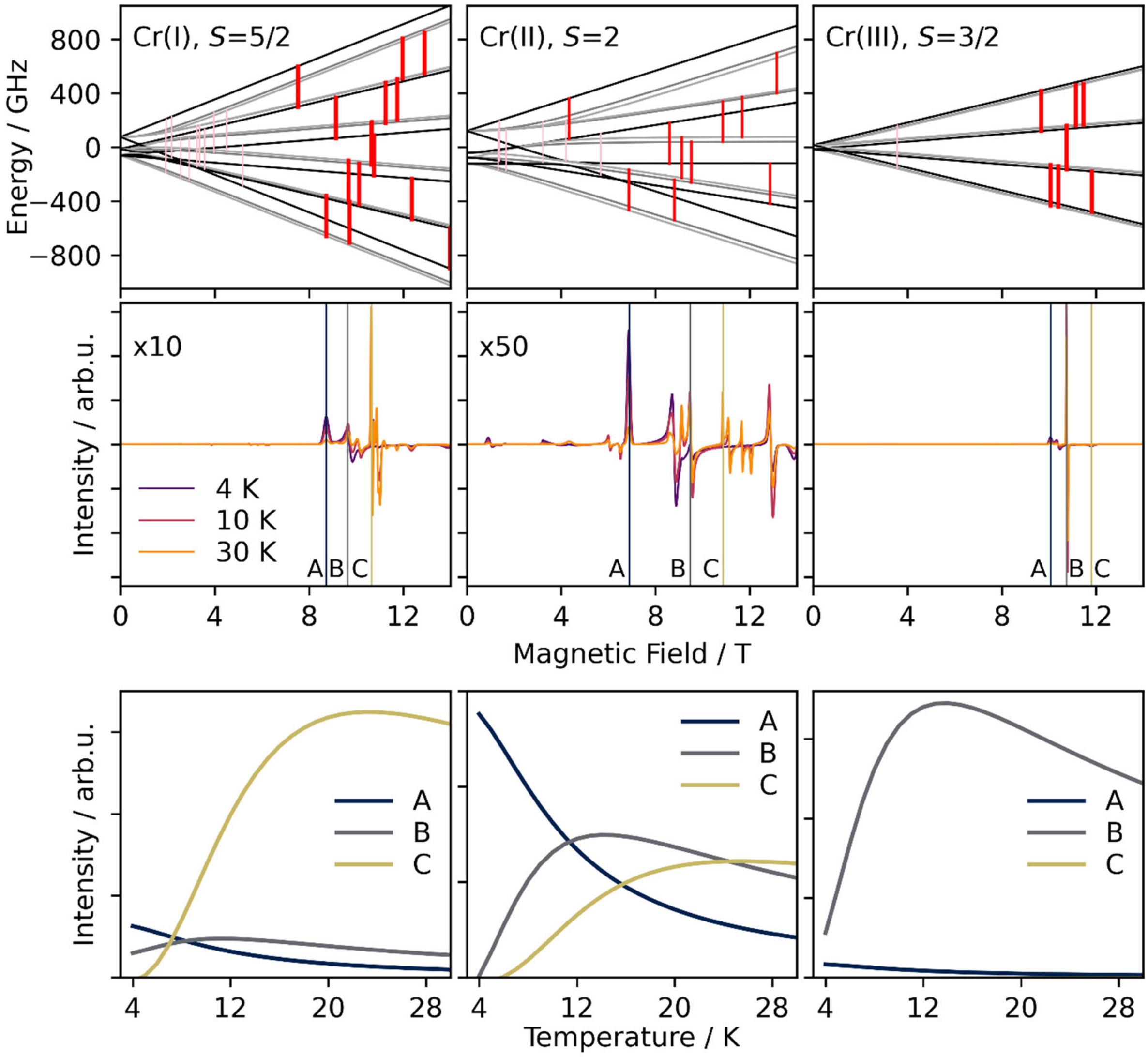

Figure S14. (top) Zeeman diagram of the spin microstate energies of different relevant oxidation/spin states of chromium for positive D-values as a function of applied field for the magnetic field B_0 parallel to the molecular z-axis (black) and perpendicular to it (grey) with EPR transitions shown in different shades of red, where a darker shade indicates a more allowed transition. (middle) HFEPR spectra calculated for these species, using typical spin Hamiltonian parameters. (bottom) Calculated resonance line intensity for characteristic transitions as a function of temperature.

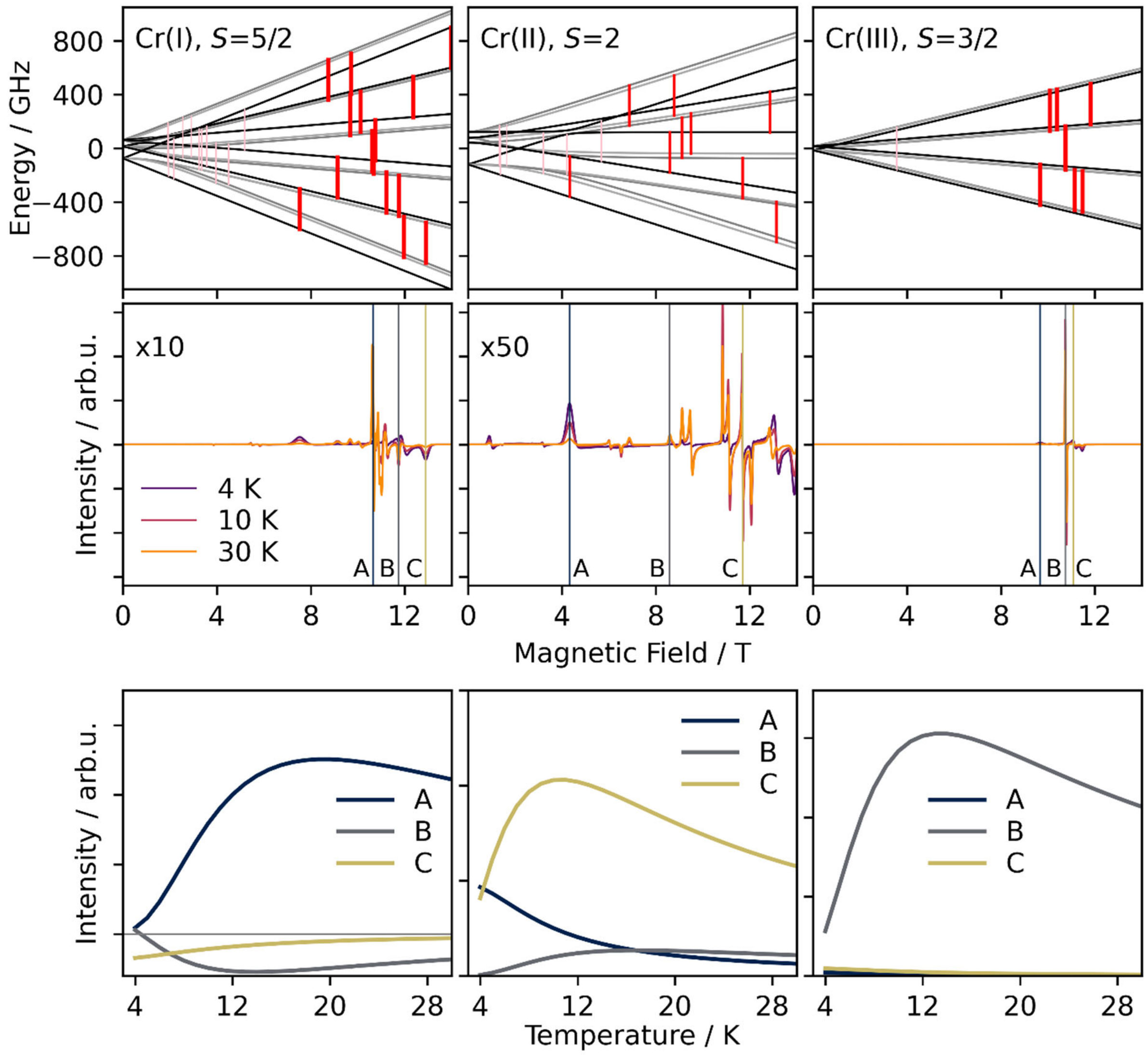

Figure S15. (top) Zeeman diagram of the spin microstate energies of different relevant oxidation/spin states of chromium for positive *D*-values as a function of applied field for the magnetic field B0 parallel to the molecular z-axis (black) and perpendicular to it (grey) with EPR transitions shown in different shades of red, where a darker shade indicates a more allowed transition. (middle) HFEPR spectra calculated for these species, using typical spin Hamiltonian parameters. (bottom) Calculated resonance line intensity for characteristic transitions as a function of temperature.

3.1 HFEPR measurements on Cr-acac

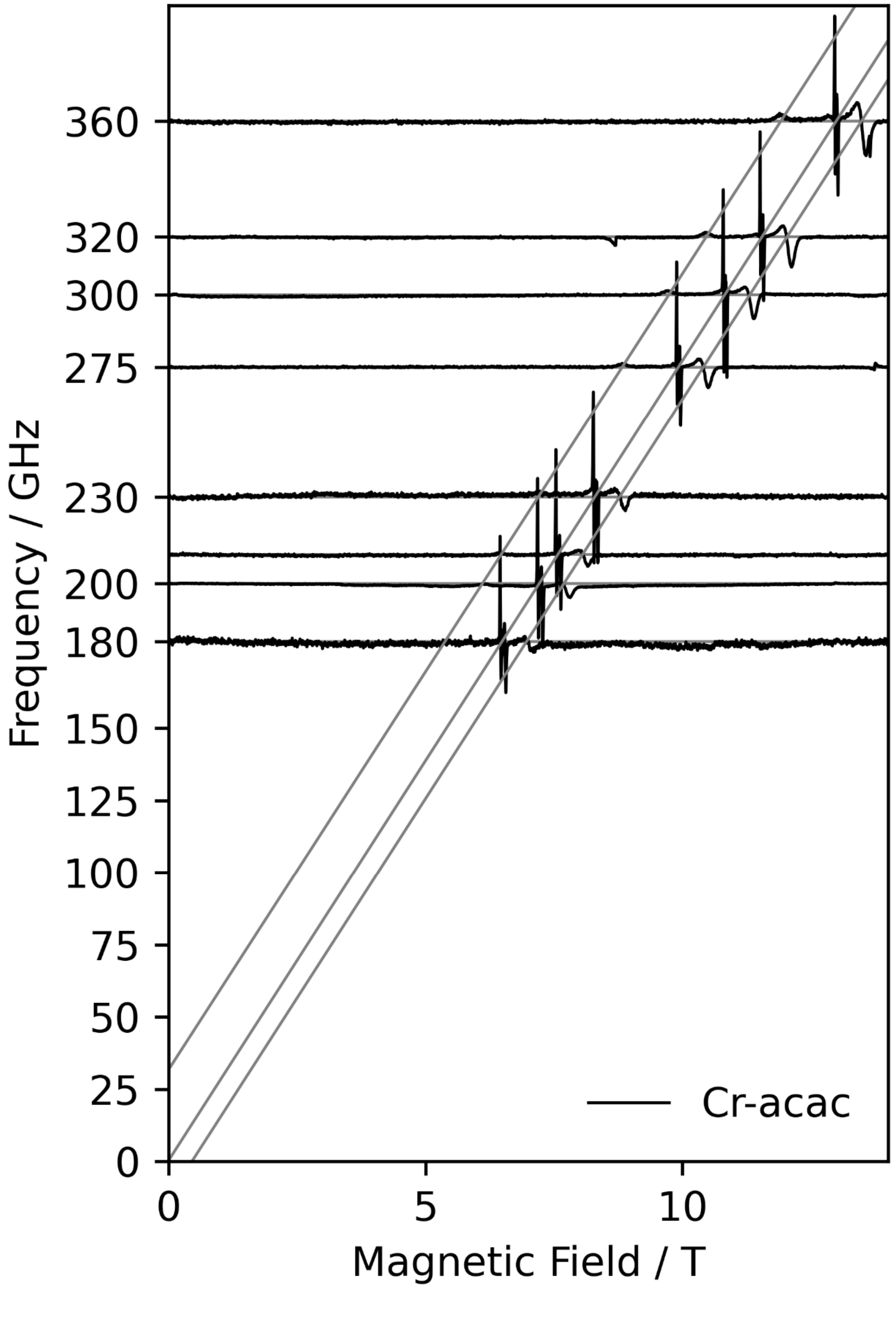

Figure S16. HFEPR measurements of **Cr-acac** in frozen toluene solution measured at multiple frequencies and at 4 K.

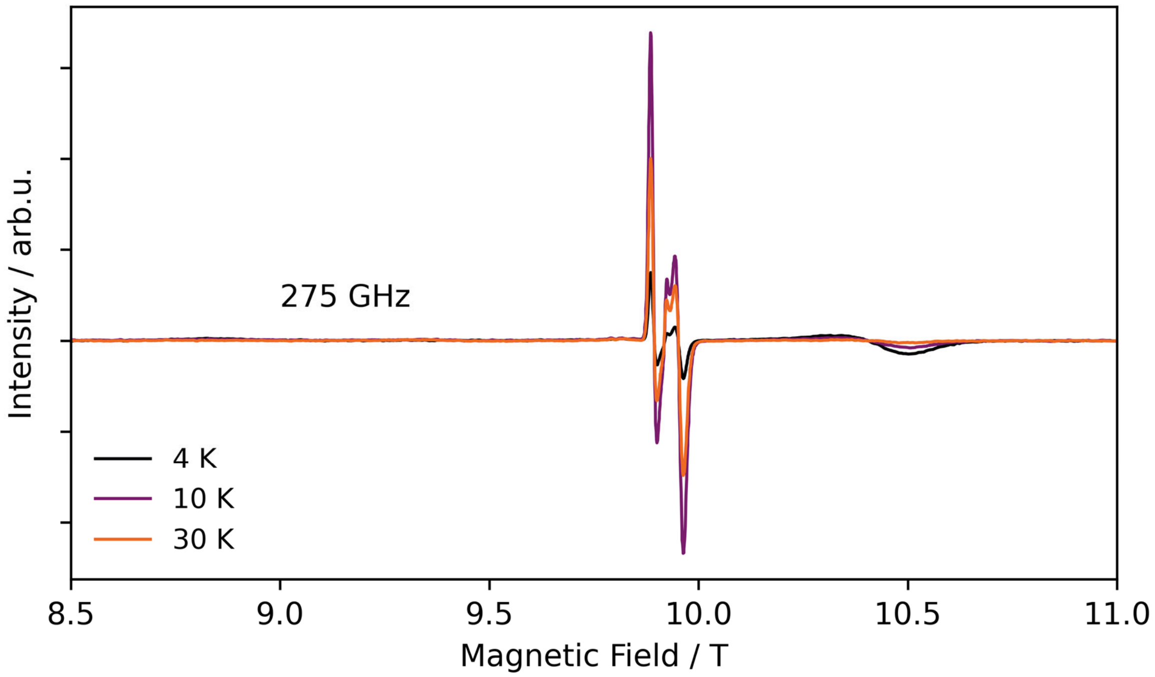

Figure S17. HFEPR measurements of **Cr-acac** in frozen solution (toluene) measured at 275 GHz and at various temperatures to illustrate the temperature dependence of the signals in the spectrum.

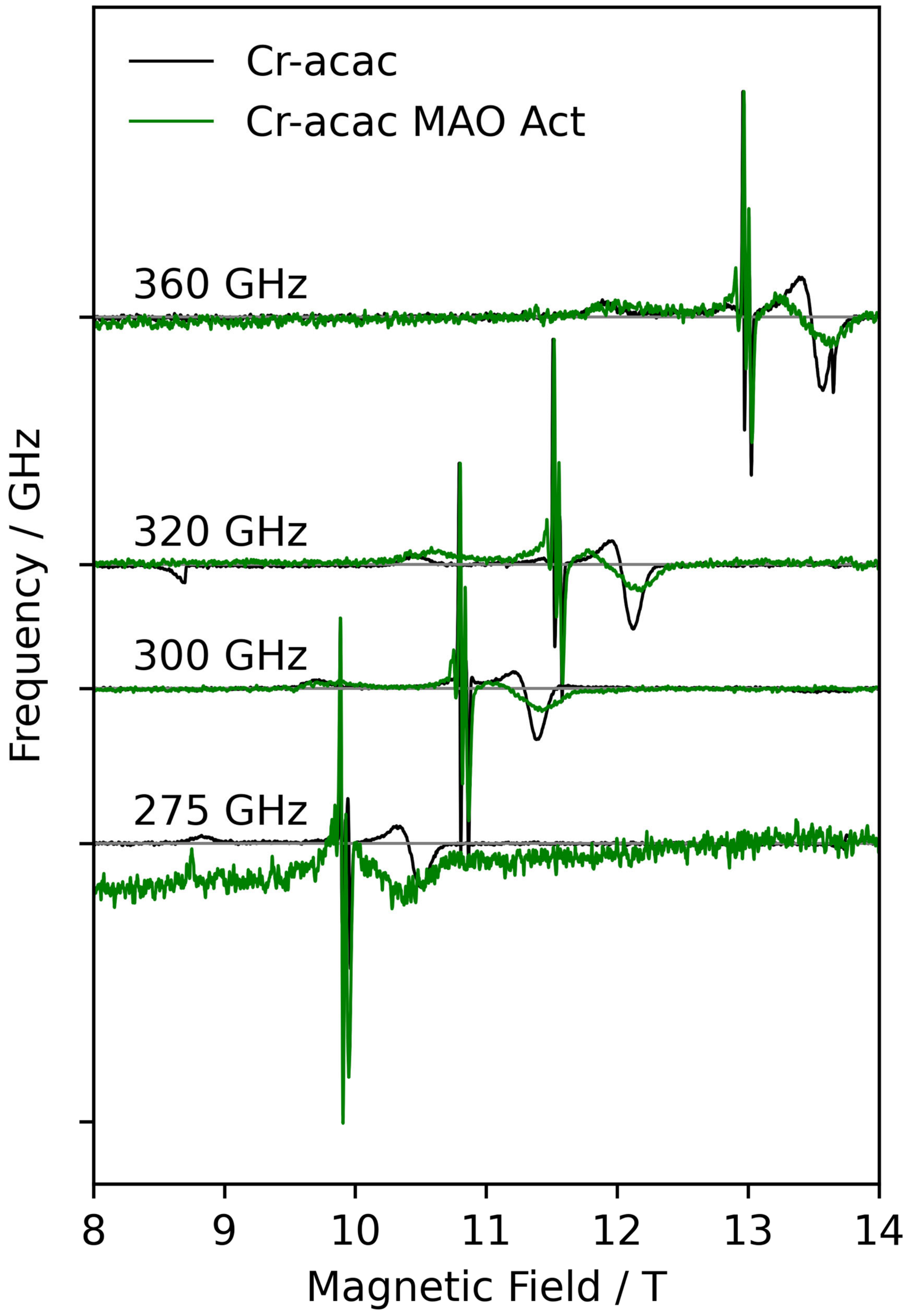

Figure S18. HFEPR measurements of **Cr-acac** activated with MAO in solution (toluene) measured at multiple frequencies at 5 K with in comparison to measurements of the non-activated **Cr-acac** under the same conditions.

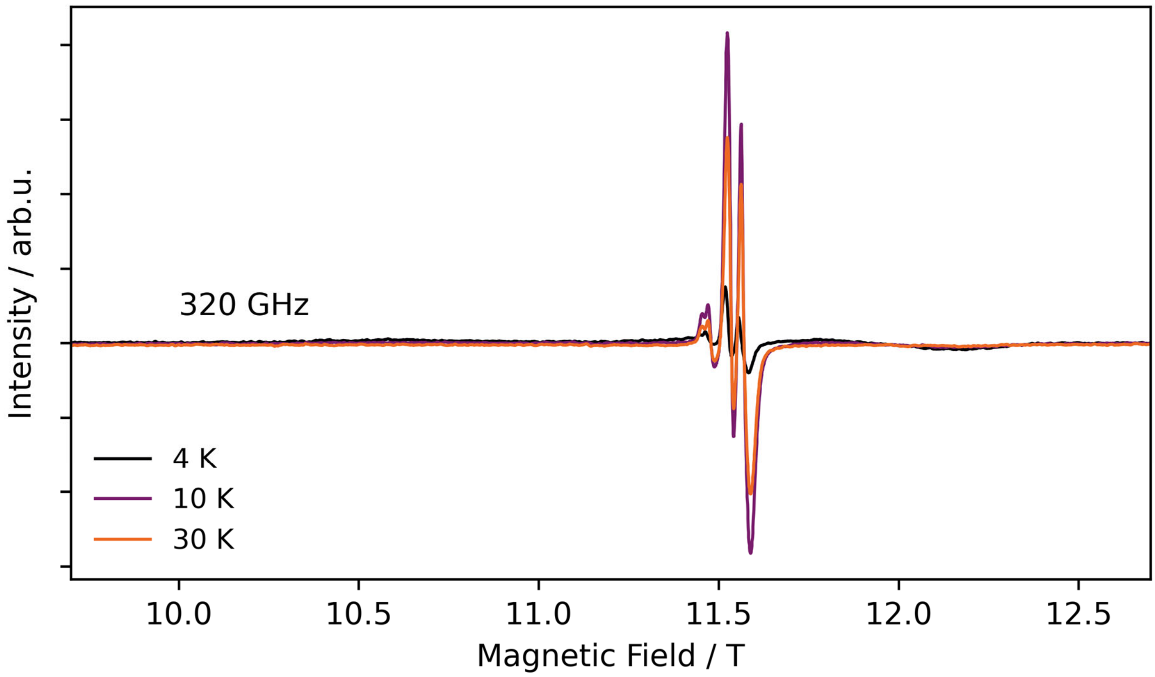

Figure S19. HFEPR measurements of **Cr-acac** activated with MAO in solution (toluene) measured at 320 GHz at temperatures between 5 K and 30 K.

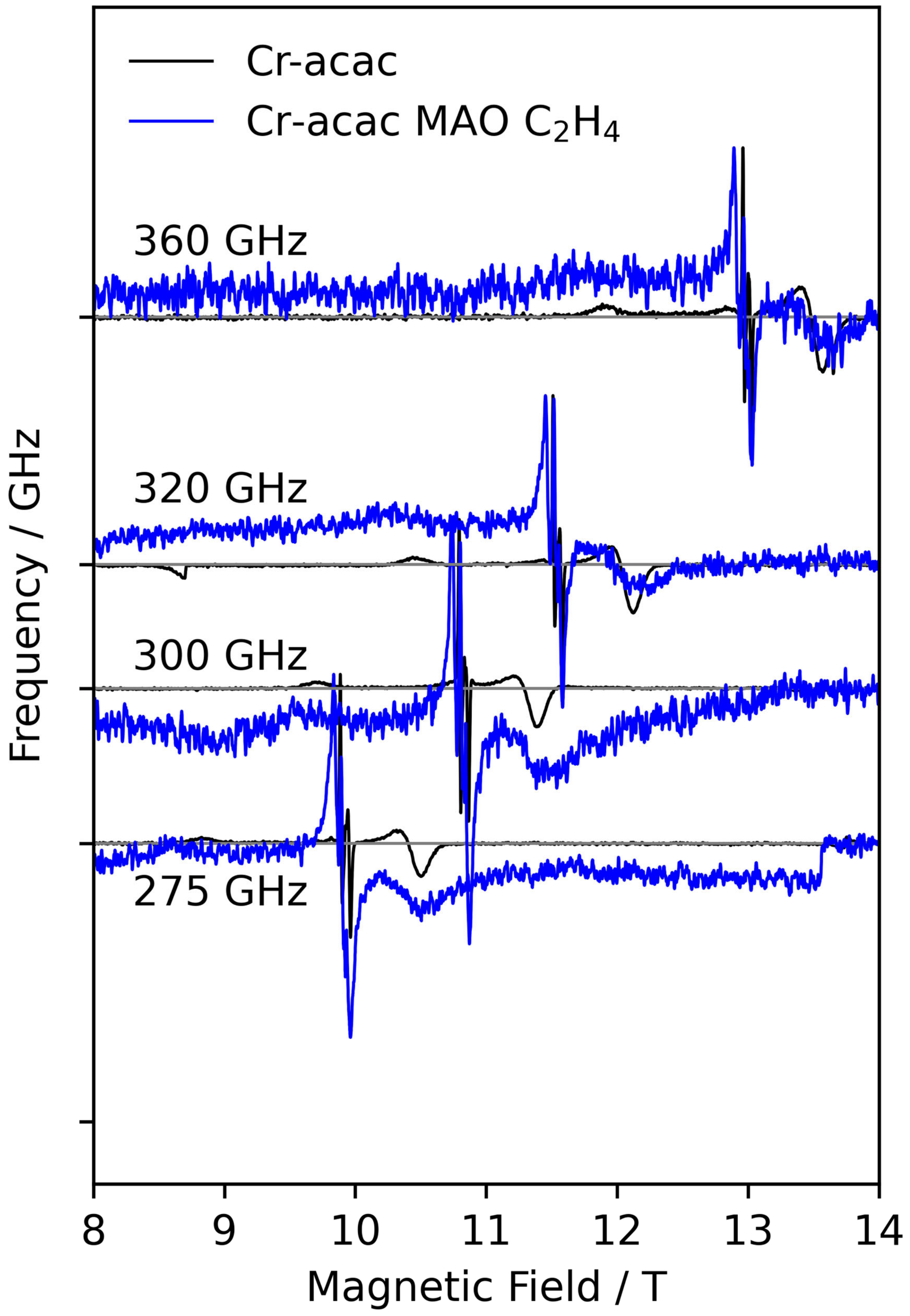

Figure S20. HFEPR measurements of **Cr-acac** activated with MAO in the presence of ethylene in solution (toluene) measured at multiple frequencies, and at 4 K with a comparison to measurements of the non-activated **Cr-acac** under the same conditions.

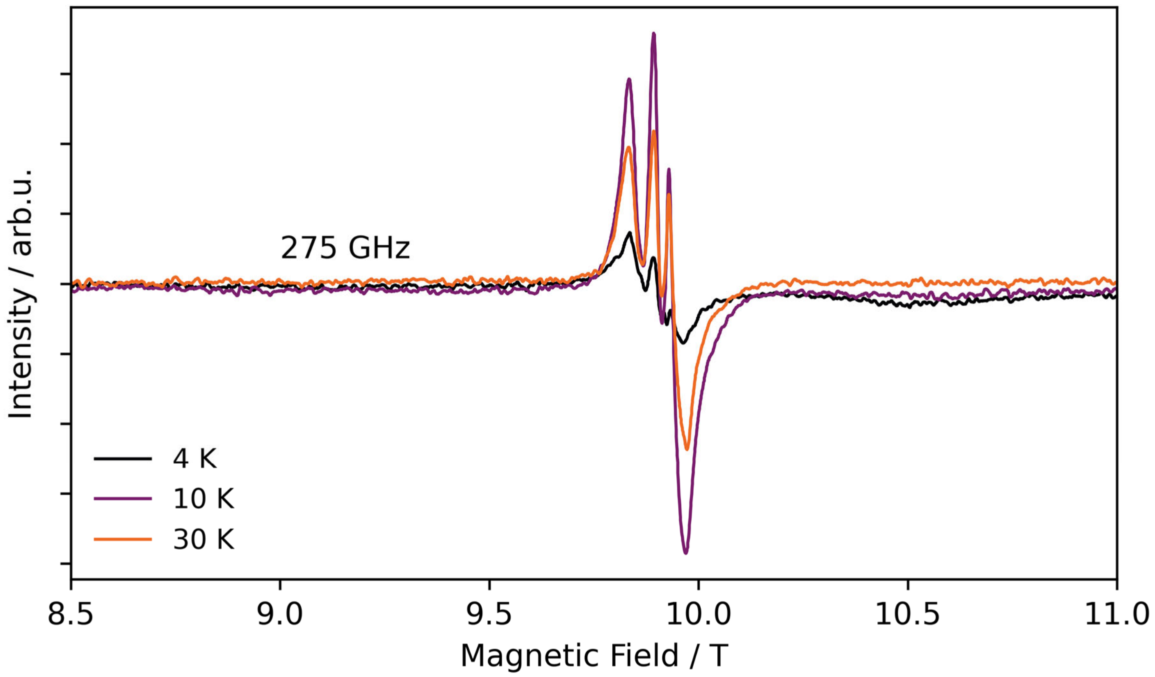

Figure S21. HFEPR measurements of **Cr-acac** activated with MAO in the presence of ethylene in solution (toluene) measured at 320 GHz at temperatures between 5 K and 30 K.

3.2 HFEPR measurements on Cr-PNP

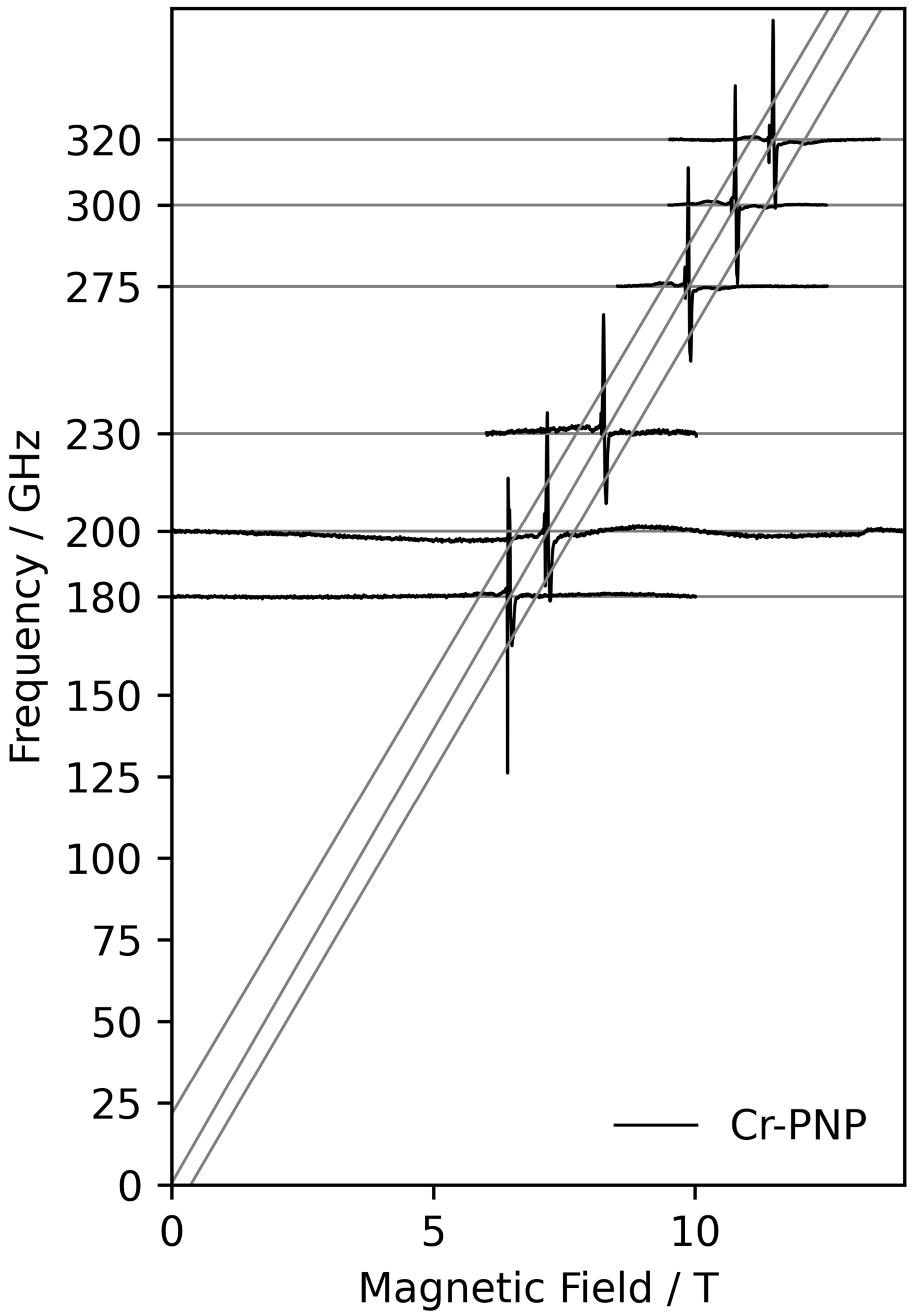

Figure S22. HFEPR measurements of **Cr-PNP** in solution (toluene:DCM) measured at multiple frequencies, and at 5 K.

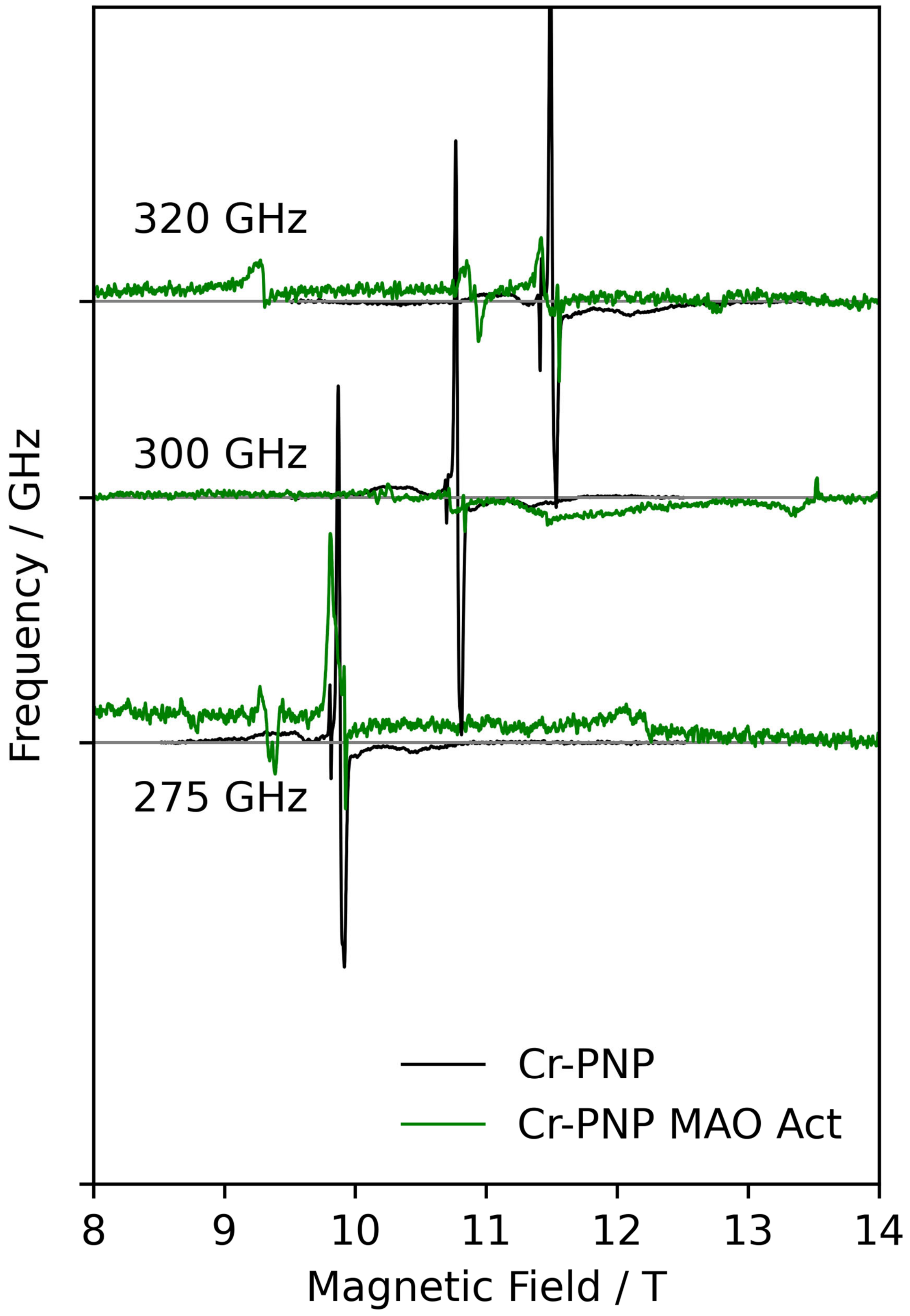

Figure S23. HFEPR measurements of **Cr-PNP** in solution (toluene:DCM) activated with MAO measured at various frequencies at 5 K (blue) in comparison to the non-activated complex (green).

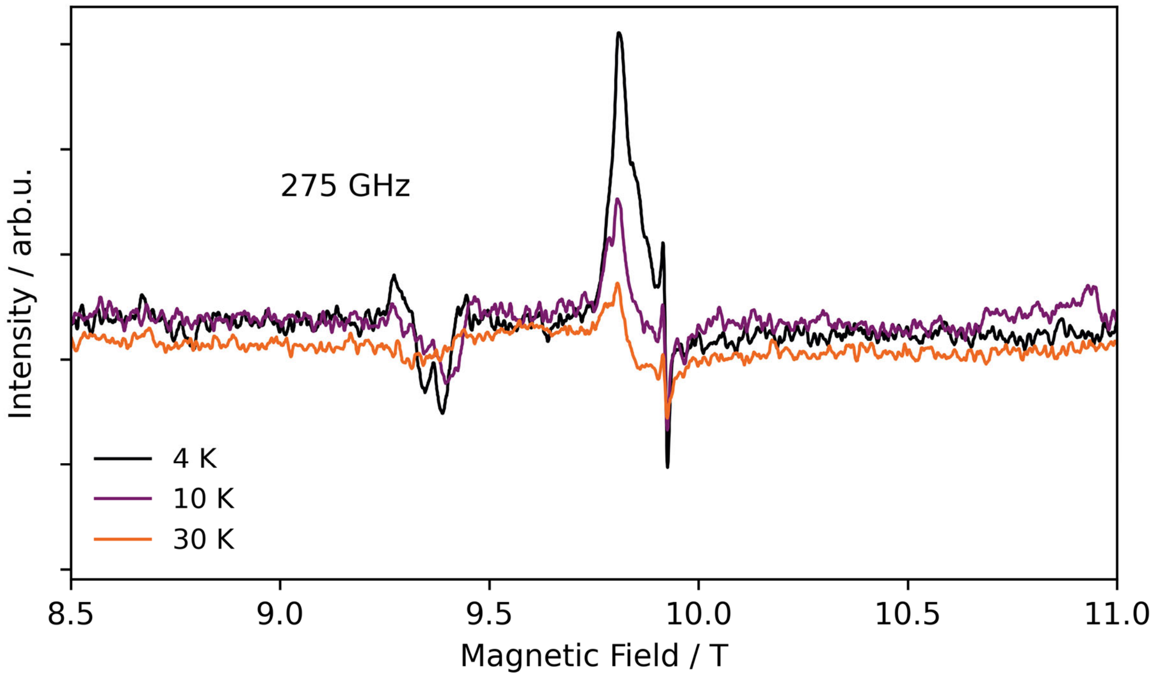

Figure S24. HFEPR measurements of **Cr-PNP** activated with MAO in solution (toluene) measured at 275 GHz, and at temperatures between 4 K and 30 K.

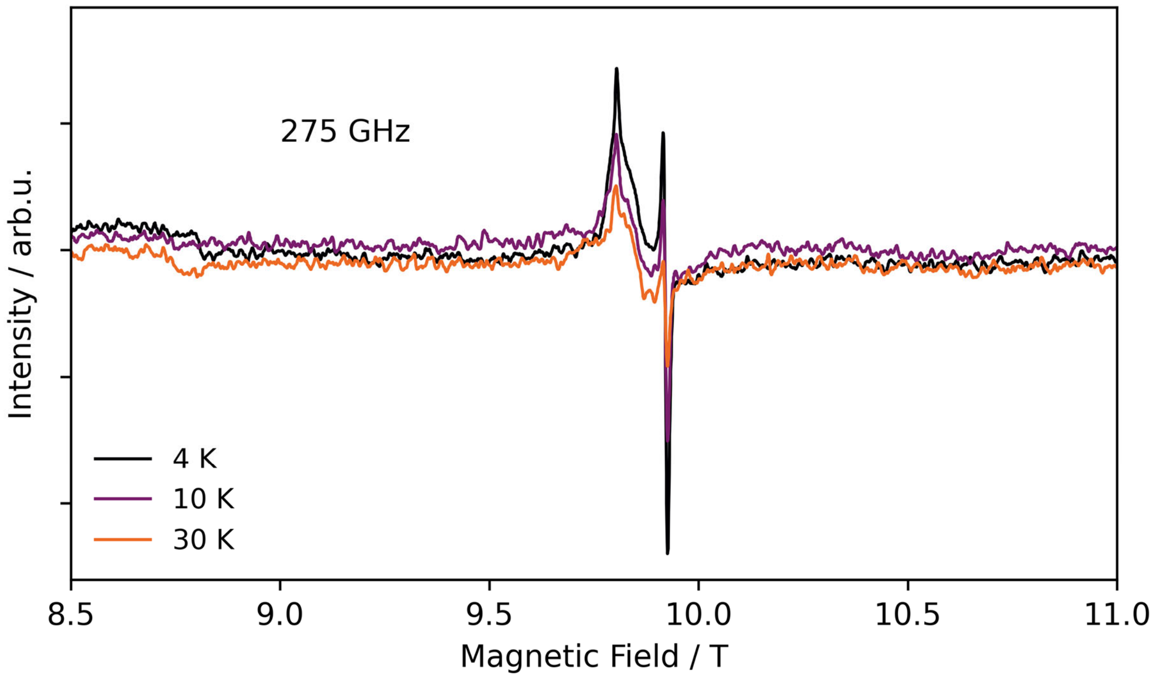

Figure S25. HFEPR measurements of **Cr-PNP** activated with MAO in the presence of ethylene in solution (toluene) measured at 275 GHz, and at temperatures between 4 K and 30 K.

3.3 SQUID and HFEPR measurements on Cr-CAAC

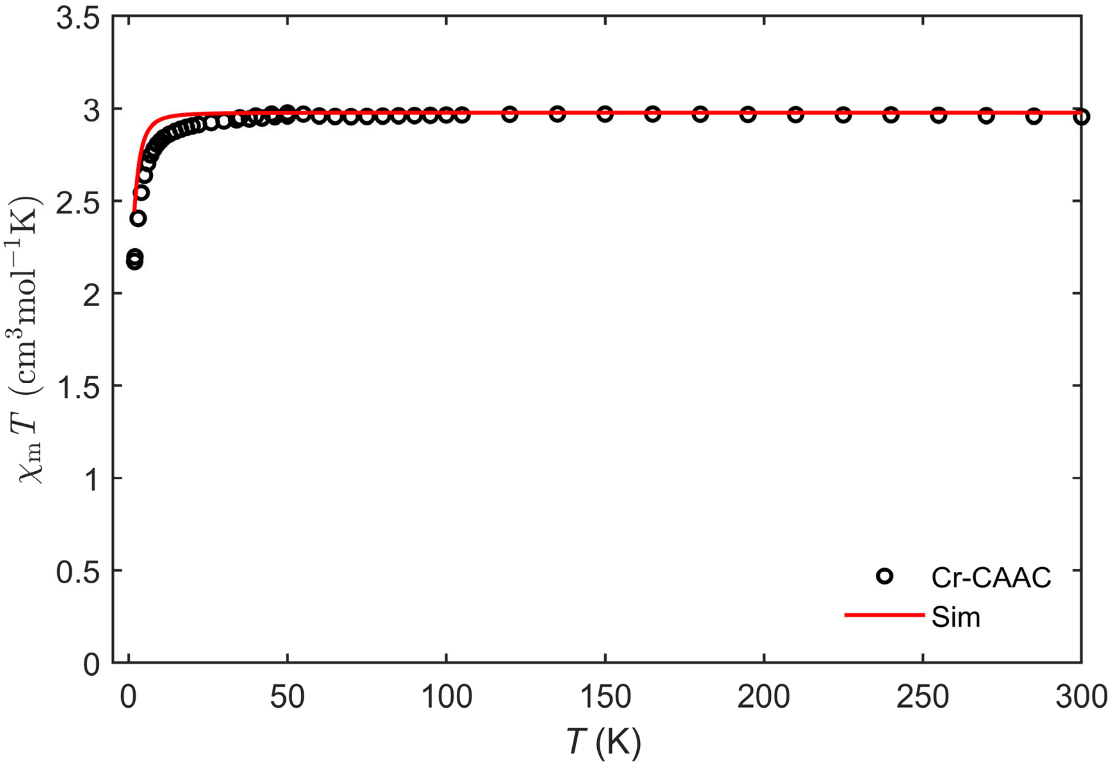

Figure S26. SQUID measurements of the paramagnetic susceptibility χT on a solid-state sample of **Cr-CAAC** with the simulation based on the parameters in **Table 1** in the main text.

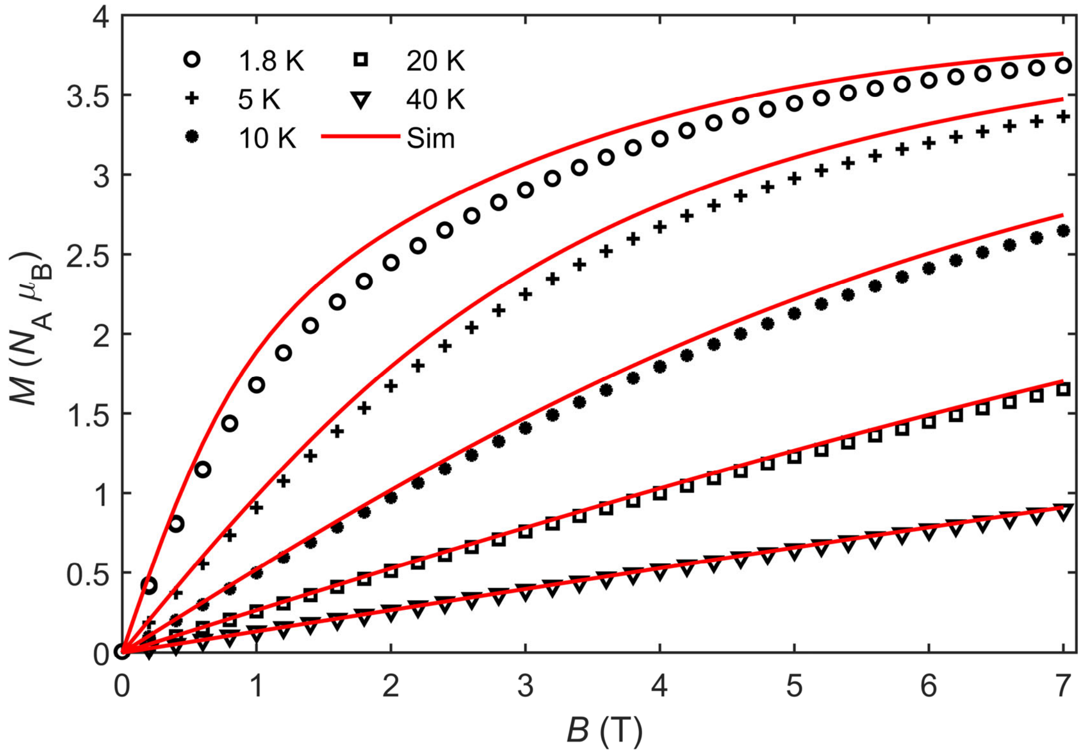

Figure S27. SQUID measurements of the magnetization M on a solid-state sample of **Cr-CAAC** with the simulation based on the parameters in **Table 1** in the main text.

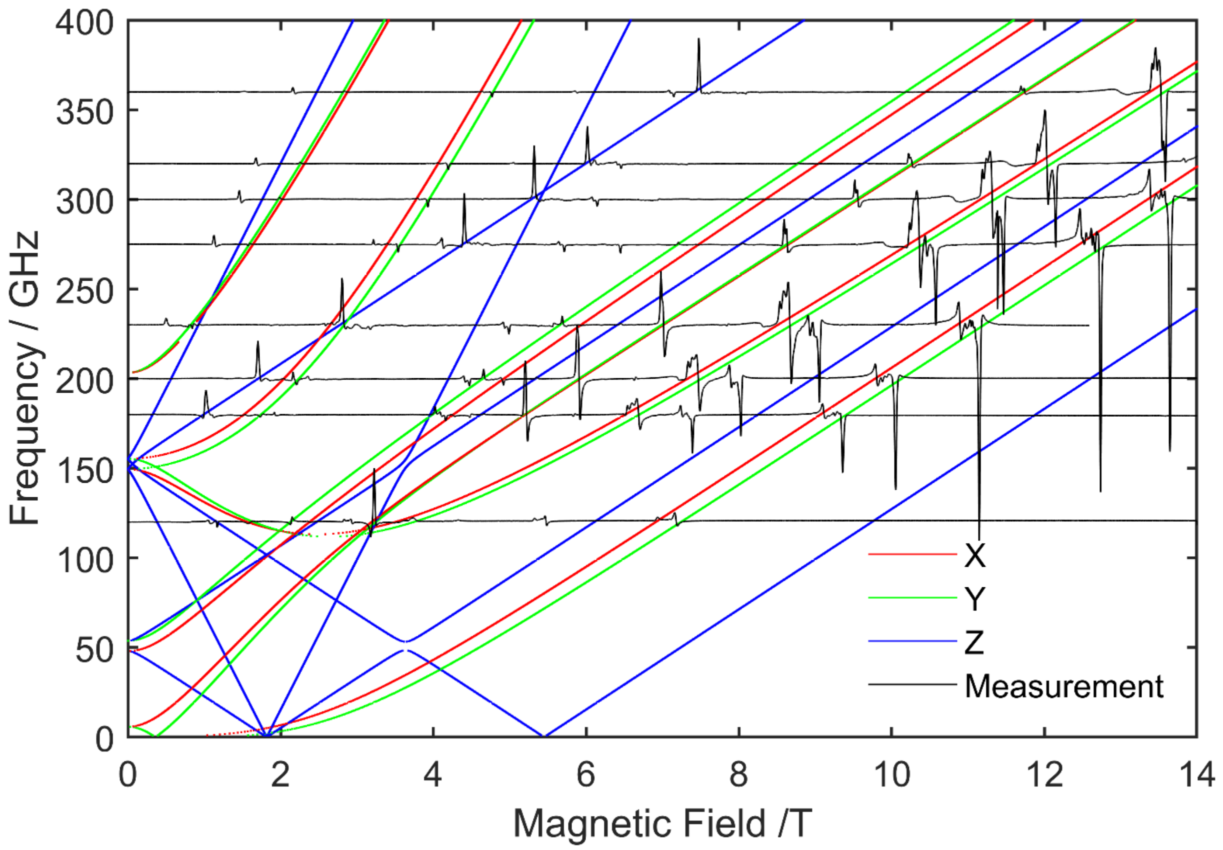

Figure S28. HFEPR measurements of **Cr-CAAC** in solid state (black) measured at different frequencies between 120 – 320 GHz at 4 K, together with the field dependence of the transition frequencies along the different Cartesian axes in the molecular coordinate frame

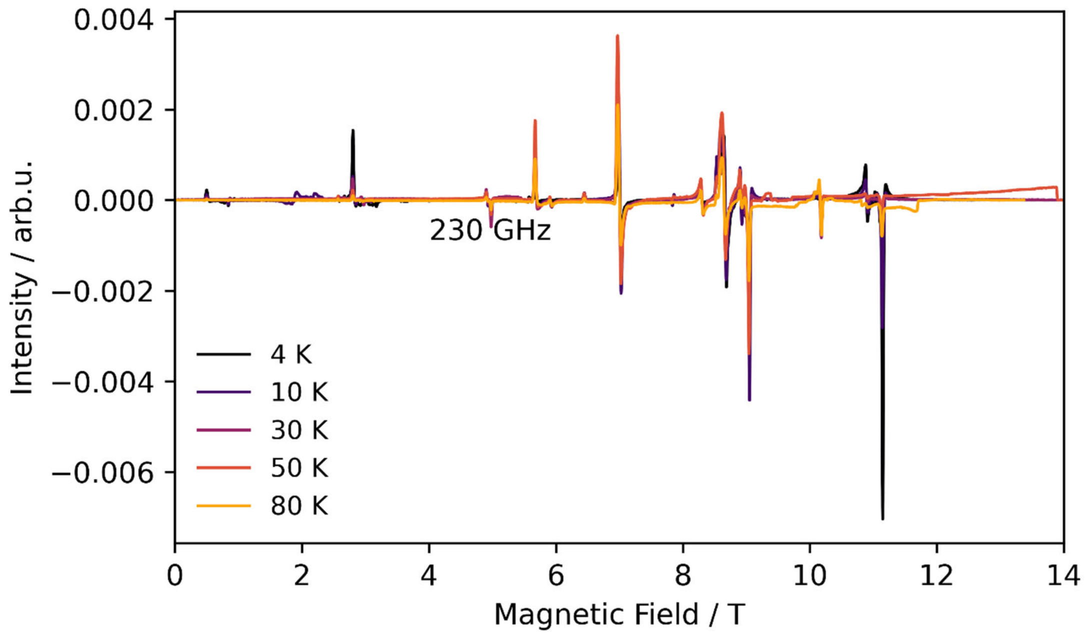

Figure S29. HFEPR measurements of **Cr-CAAC** in the solid state measured at different temperatures between 4 – 80 K and at 230 GHz,

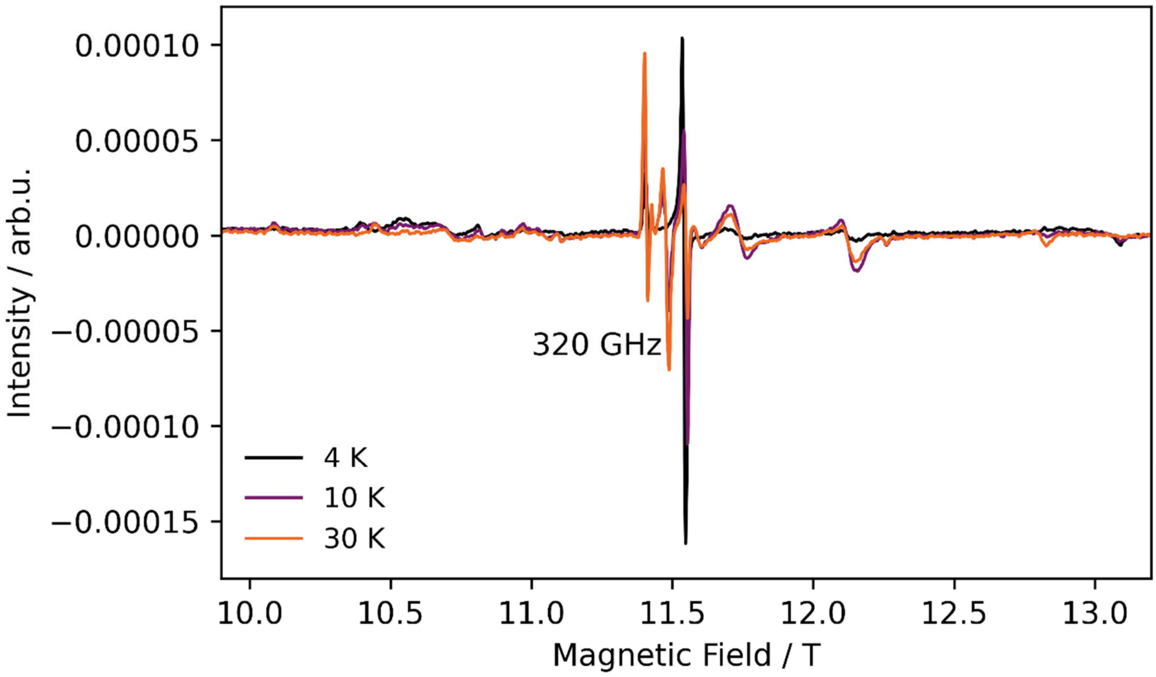

Figure S30. HFEPR measurements of Cr-CAAC activated with MAO in solution (Toluene) measured with 320 GHz at temperatures between 4 K and 30 K

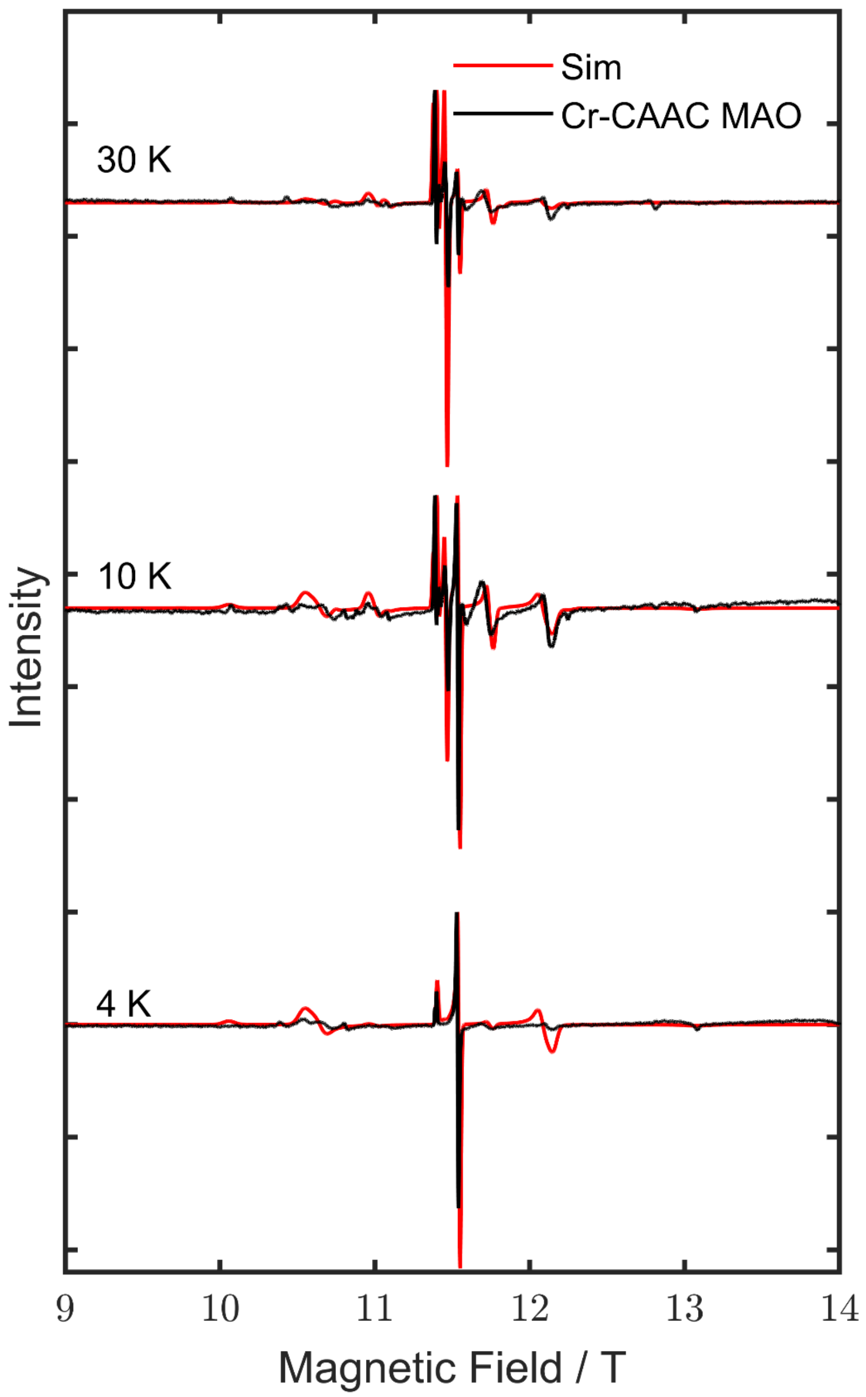

Figure S31. HFEPR measurements on a frozen solution sample of **Cr-CAAC** activated with MAO, at various temperatures, and at 320 GHz with simulations of multiple species based on the parameters in **Table S5**.

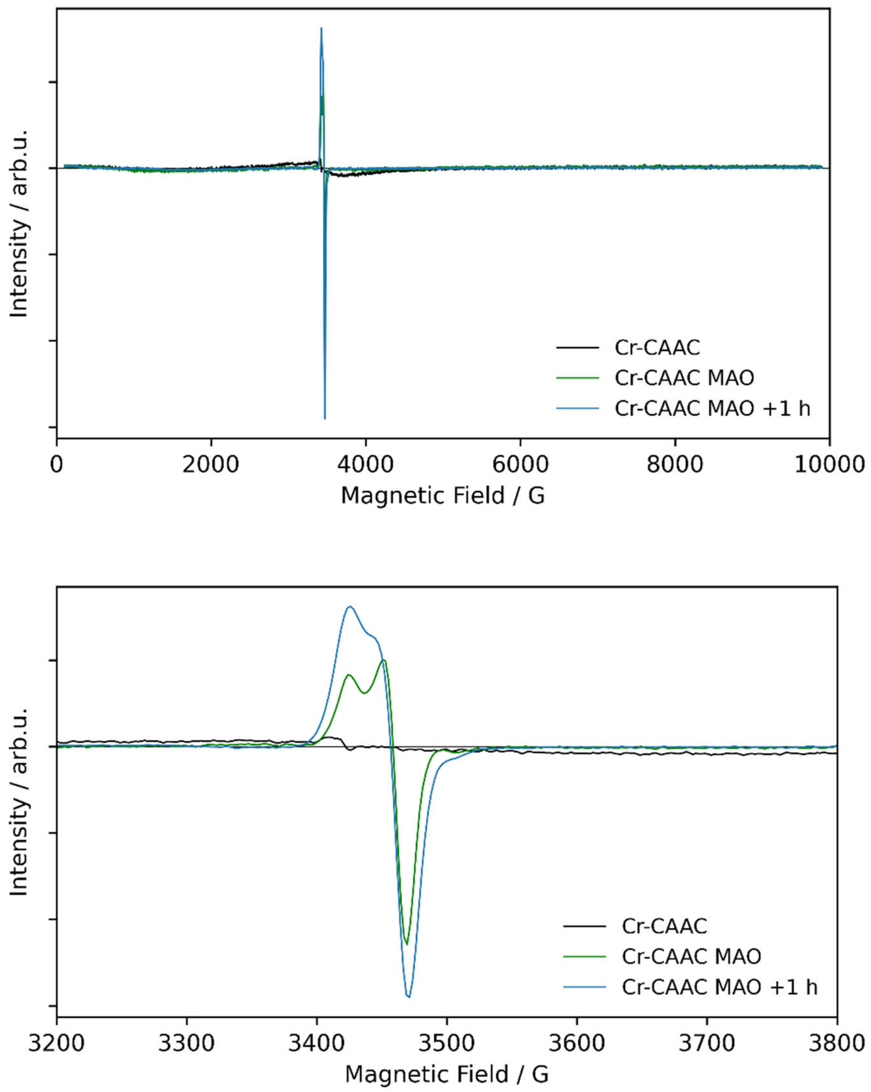

Figure S32. X-Band EPR measurements on Cr-CAAC in toluene before and after activation with MAO at 7 K.

Table S5. Parameters for the spin Hamiltonian simulation of the HFEPR spectra obtained for **Cr-CAAC** activated with MAO measured in frozen solution shown in **Figure S31**.

Parameter	Value(s) System 1	Value(s) System 2	Value(s) System 3
S	5/2	5/2	1/2
g_x	2.000(2)	2.001(2)	2.005(1)
g_y	2.002(2)	2.004(2)	1.980(1)
g_z	2.000(2)	2.001(2)	1.980(1)
D, *E* [cm^{-1}]	-0.33(3), 0.001(1)	0.39(3), 0.001(1)	
DStrain [cm^{-1}]	0.02(1), 0.005(1)	0.02(1), 0.001(1)	
Linewidth [mT]	15	15	15
Weight	0.33(5)	0.33(5)	0.33(5)

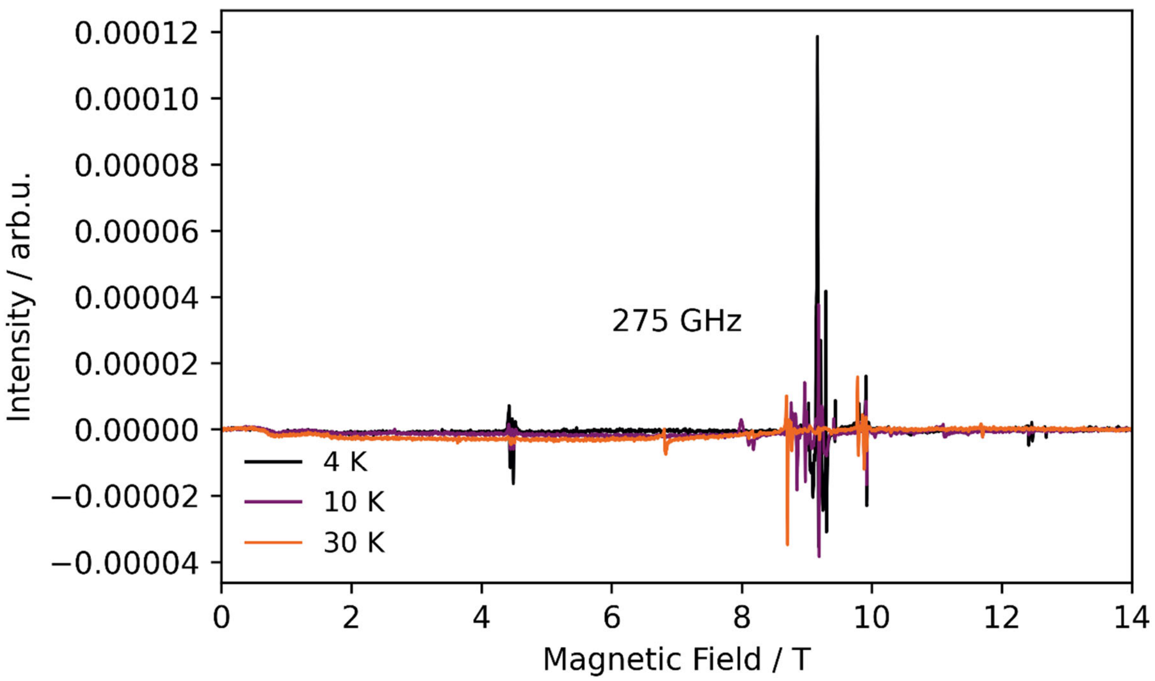

Figure S33. HFEPR measurements on a frozen solution sample of **Cr-CAAC** activated with MAO with ethylene in solution at various temperatures at 275 GHz.

3.4 SQUID and HFEPR measurements on Cr-NHC-N

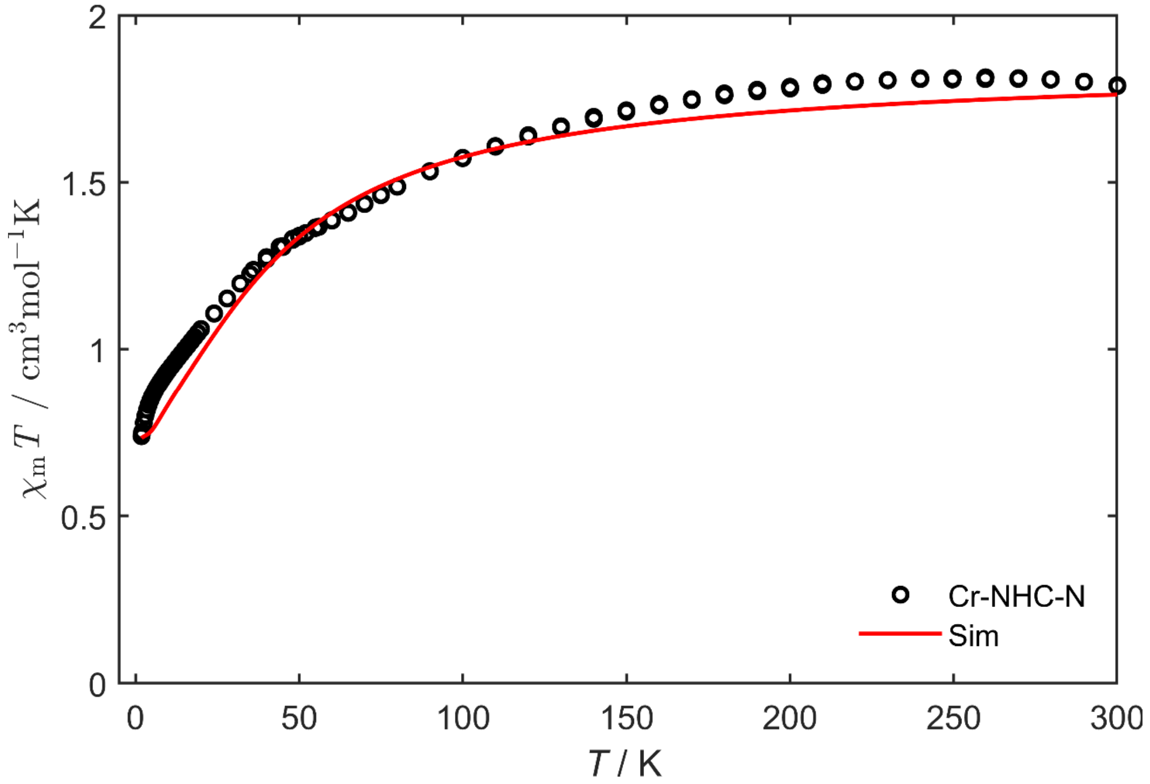

Figure S34. SQUID measurements of the paramagnetic susceptibility χT on a solid-state sample of Cr-NHC-N with the simulation with two coupled Cr(III) systems based on the parameters in **Table S6**.

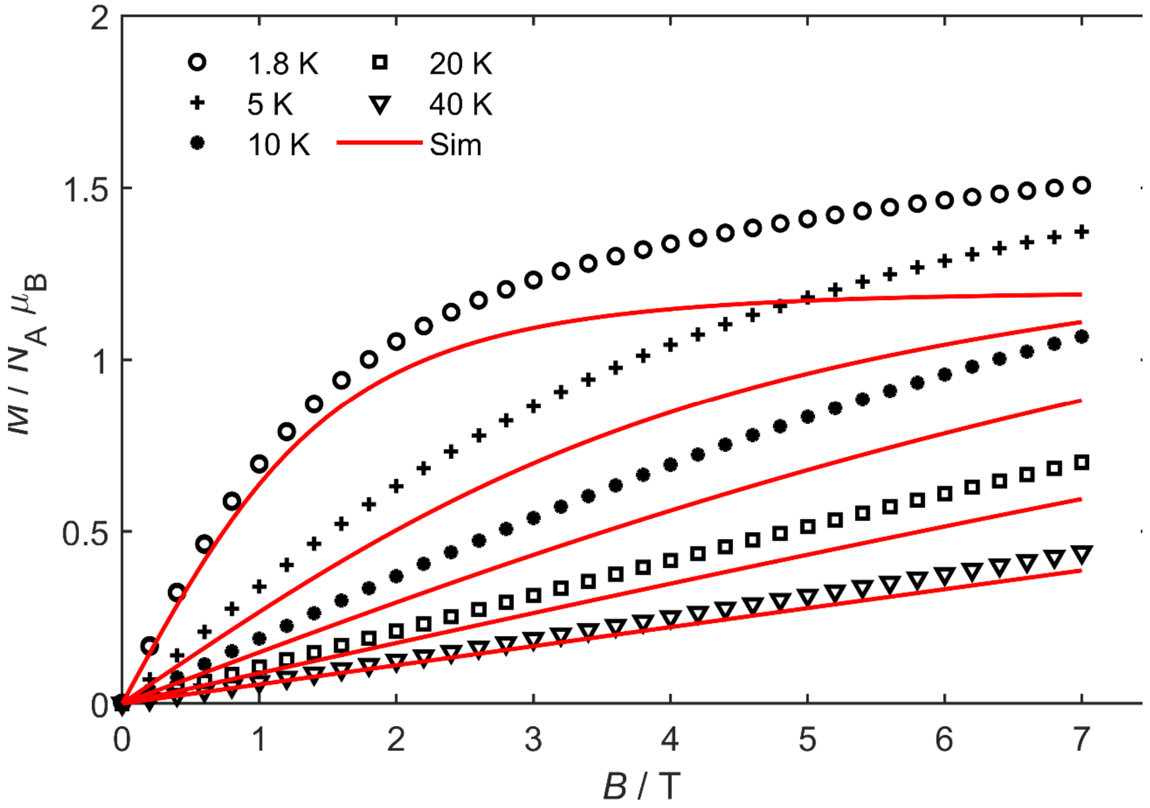

Figure S35. SQUID measurements of the magnetization *M* on a solid-state sample of Cr-NHC-N with the simulation with two coupled Cr(III) systems based on the parameters in **Table S6**.

Table S6 Parameters for the spin Hamiltonian Simulation of the SQUID data of a pellet sample of **Cr-NHC-N** shown in **Figure S34** and **Figure S35**.

Parameter	Value(s) System 1	Value(s) System 1
S	3/2	3/2 ; 3/2
g_x, g_y, g_z	1.99(1)	1.99(1) ; 1.99(1)
D, E [cm^{-1}]	0.3(1), 0.10(3)	0.3(1), 0.10(3)
J [cm^{-1}]	-	14
Weight [%]	40	60

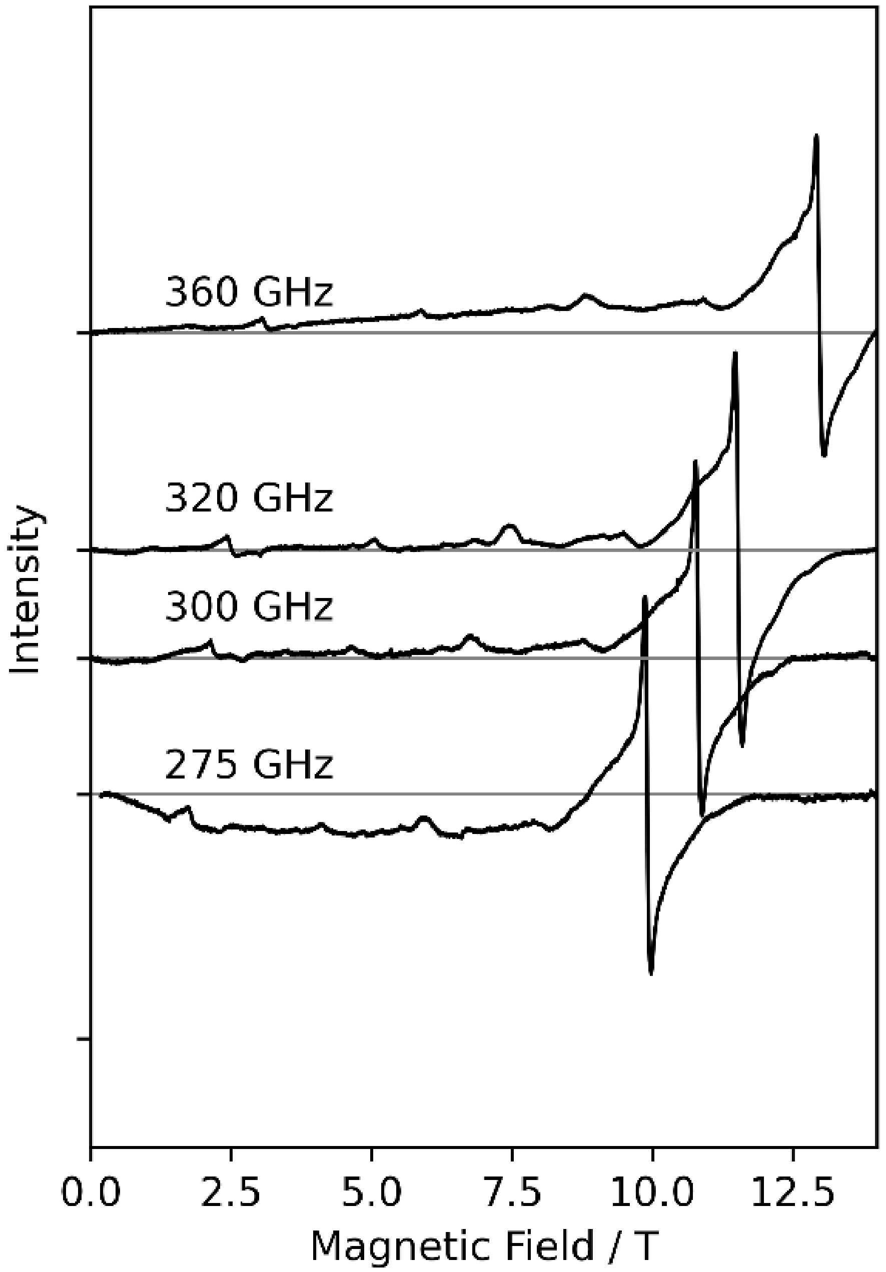

Figure S36. HFEPR measurements of **Cr-NHC-N** in solid state measured at various frequencies, and at 4 K.

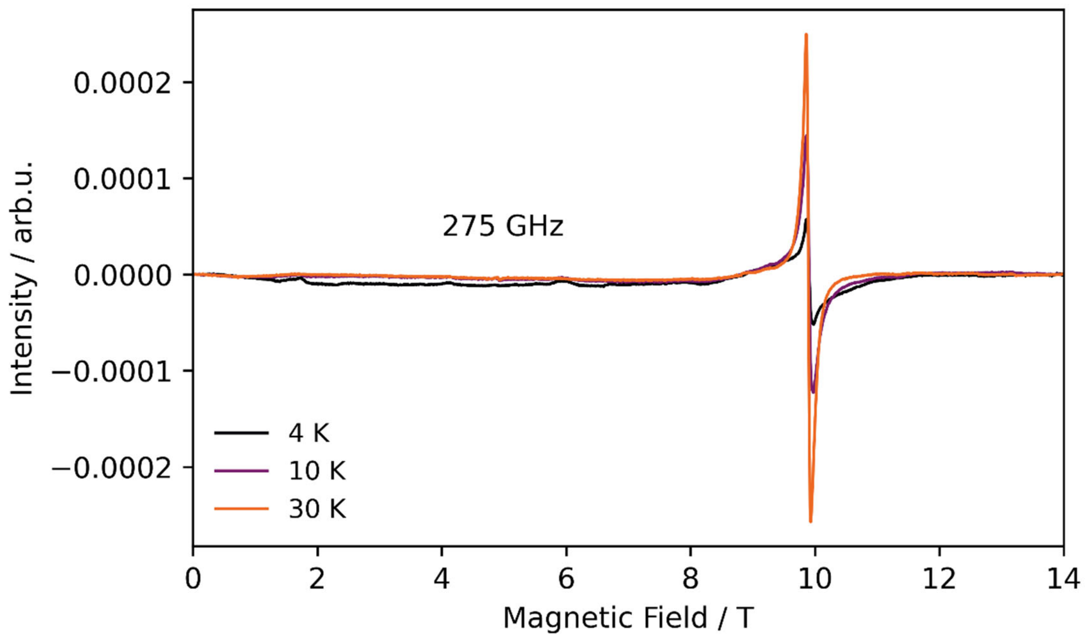

Figure S37. HFEPR measurements of Cr-NHC-N solid state measured at 275 GHz, and at different temperatures between 4 K and 30 K

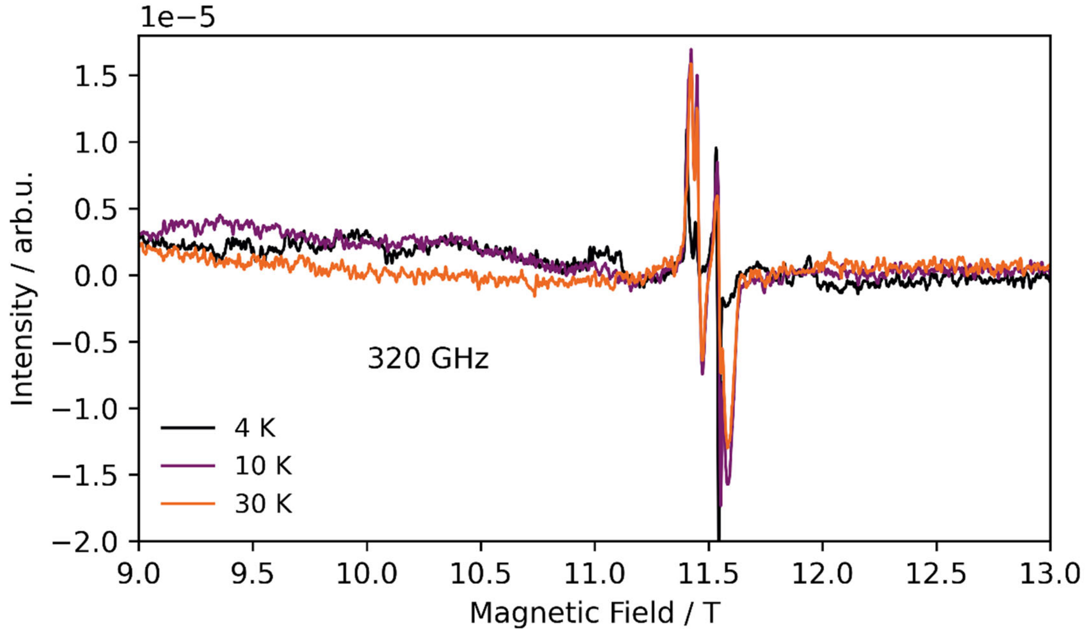

Figure S38. HFEPR measurements of **Cr-NHC-N** activated with MAO in solution (Toluene) measured at 320 GHz at temperatures between 5 K and 30 K.

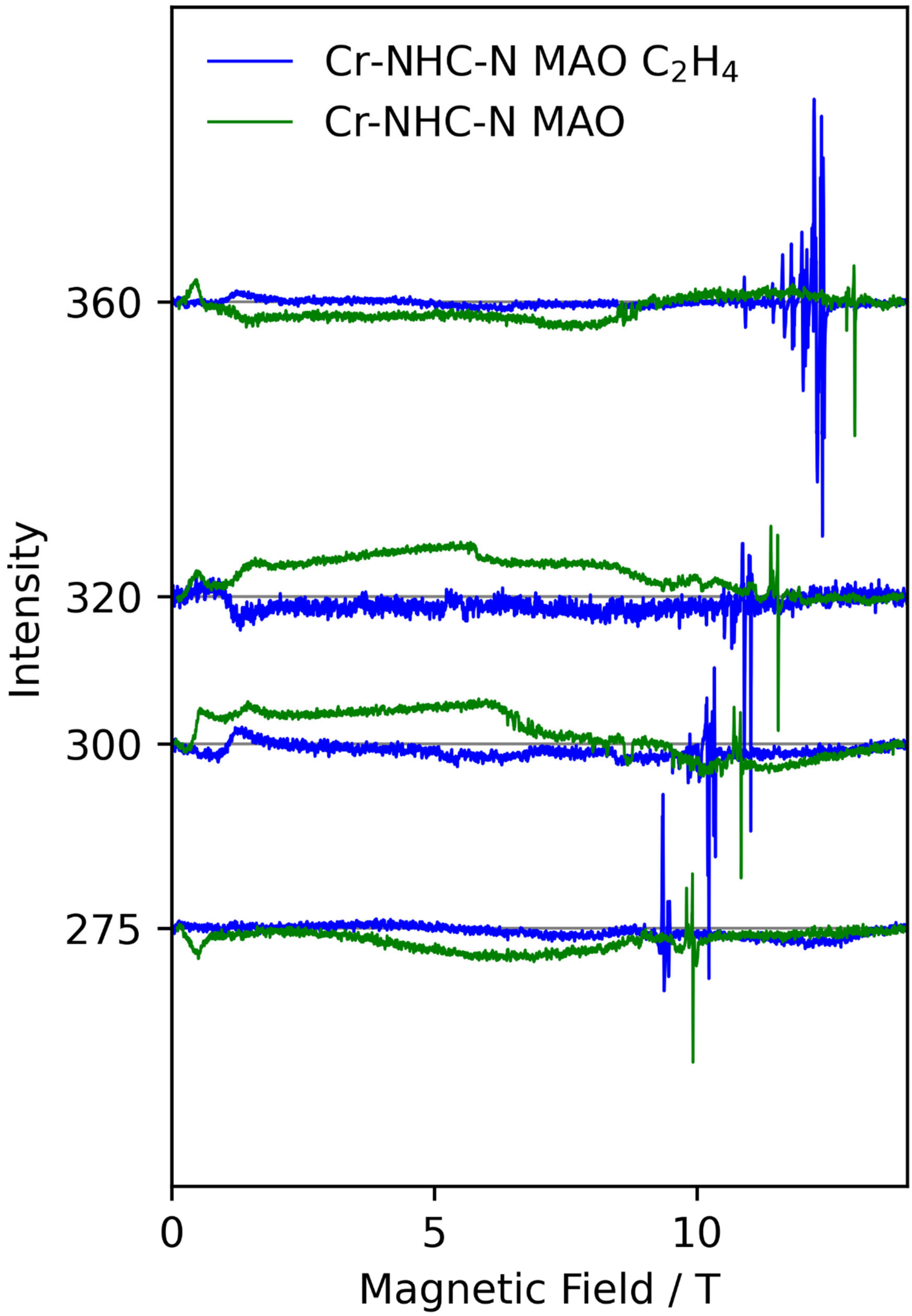

Figure S39. HFEPR measurements of **Cr-NHC-N** Dimer in frozen solution (toluene) at different stages of catalysis. The MAO activated catalyst in green and the active reaction mixture with ethylene in blue. All measurements were taken at 4 K at various frequencies (275, 300, 320 and 360 GHz).

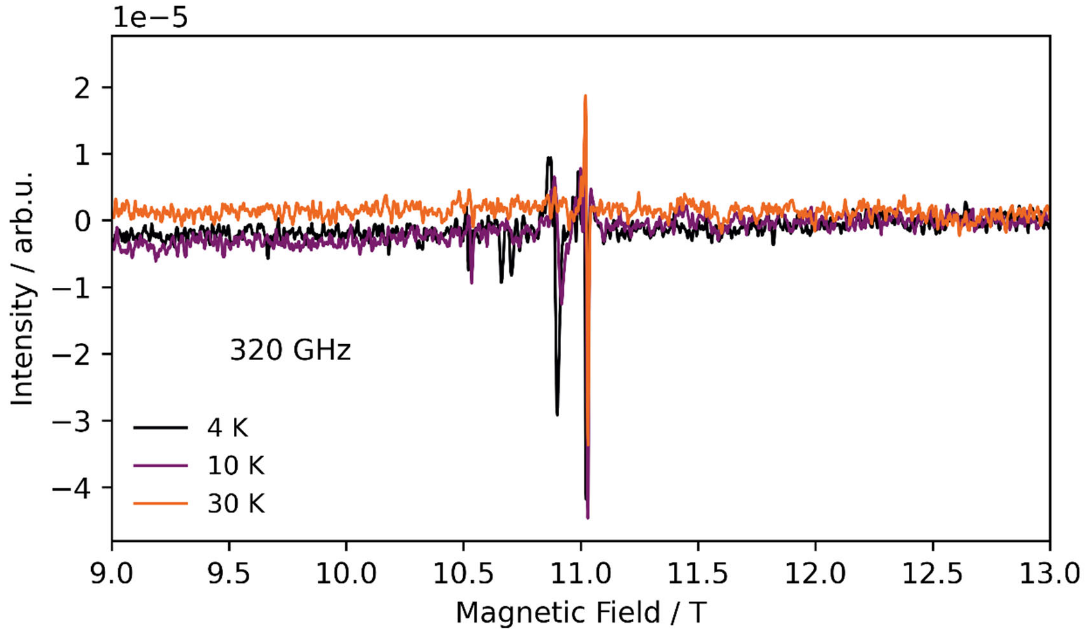

Figure S40. HFEPR measurements of Cr-NHC-N activated with MAO in presence of ethylene in solution (toluene) measured at 320 GHz at temperatures between 4 K and 30 K.

3.5 HFEPR measurements on Cr-NHC-O

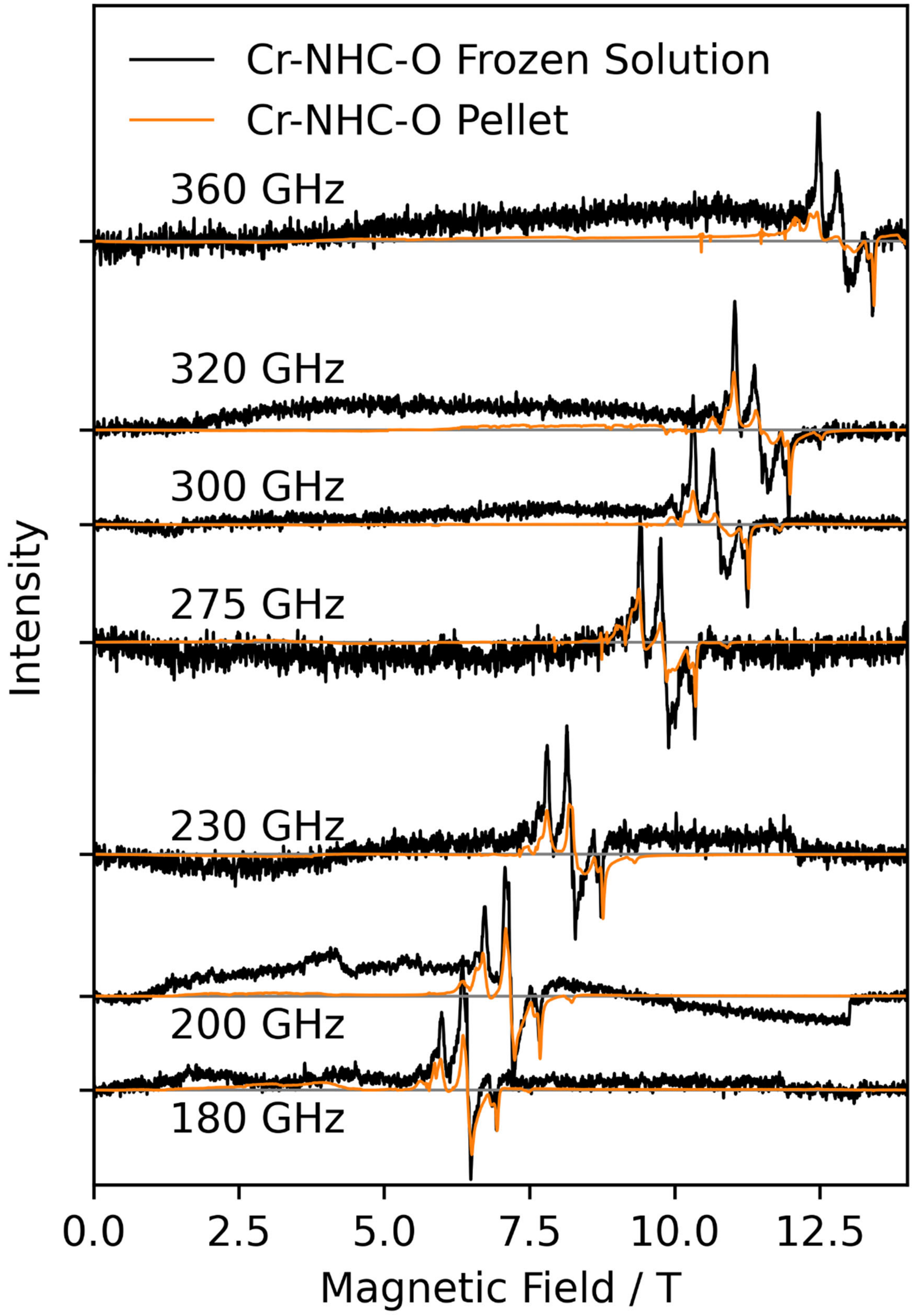

Figure S41. HFEPR measurements of **Cr-NHC-O** in frozen solution (toluene) measured with multiple frequencies at 4 K (green). In comparison to the solid state sample (black)

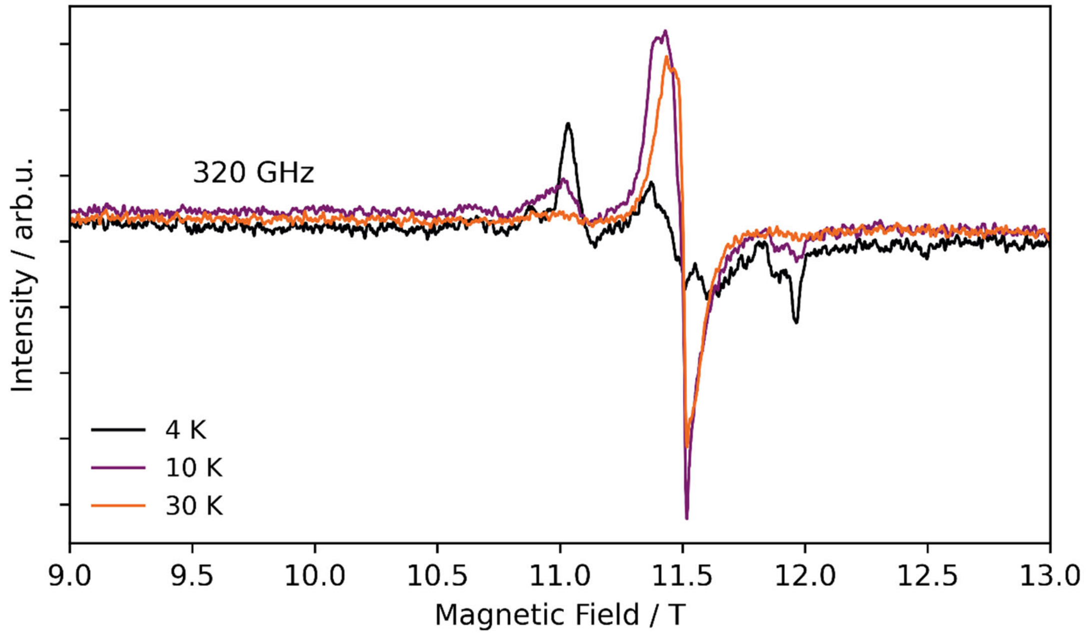

Figure S42. HFEPR measurements of **Cr-NHC-O** in frozen solution (toluene) measured with 320 GHz at temperatures between 4 K and 30 K.

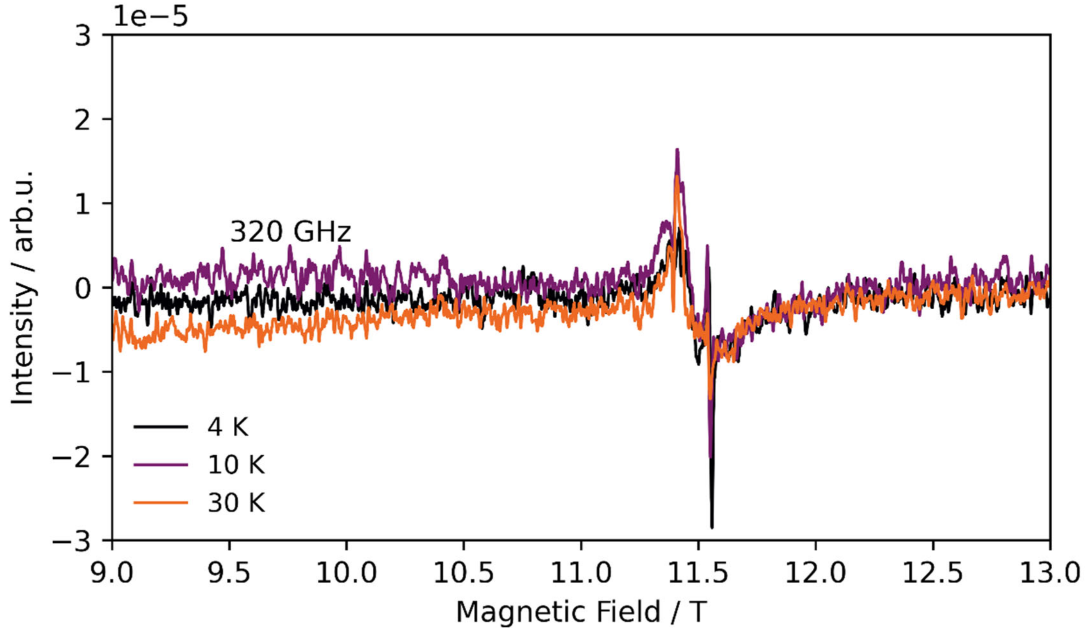

Figure S43. HFEPR measurements of **Cr-NHC-O** activated with MAO in frozen solution (toluene) measured with 320 GHz at temperatures between 4 K and 30 K.

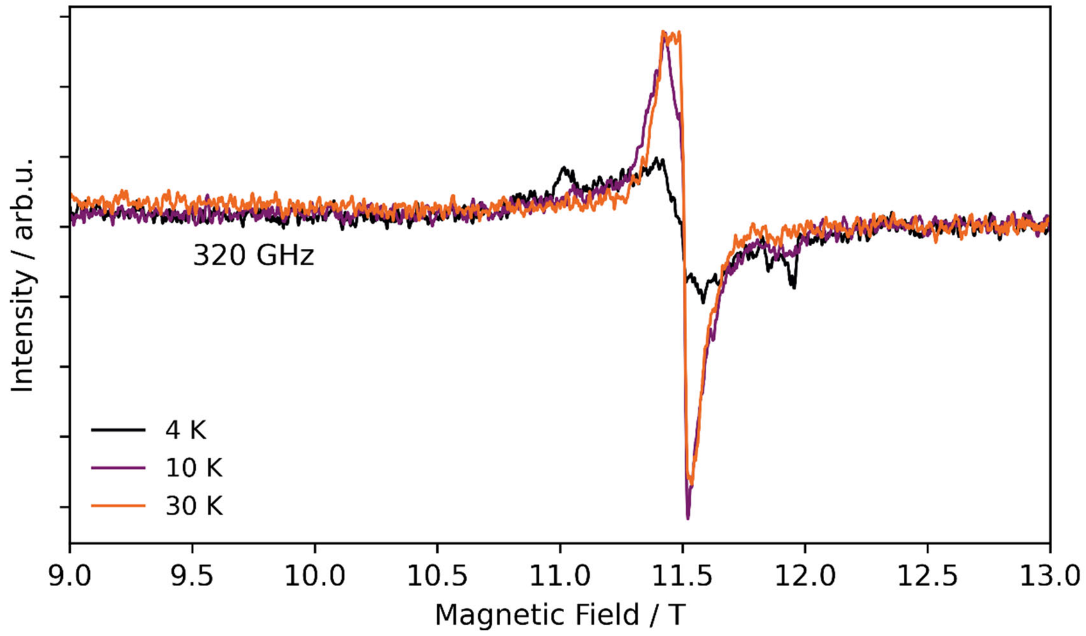

Figure S44. HFEPR measurements of **Cr-NHC-O** activated with MAO in the presence of ethylene in frozen solution (Toluene) measured with 320 GHz at temperatures between 4 K and 30 K.

4 Frozen solution HFEPR Sample holder

In preparation of this project a new sample holder for the high field EPR instrument was constructed to measure frozen solutions. The design was adapted from published work by Sojka et al.[5]

The sample is placed in a variable temperature insert (VTI) within an Oxford Instruments Teslatron 14 T superconducting magnet. The sample holder needs to enclose the sample from the vacuum environment in the instrument within a small geometry.

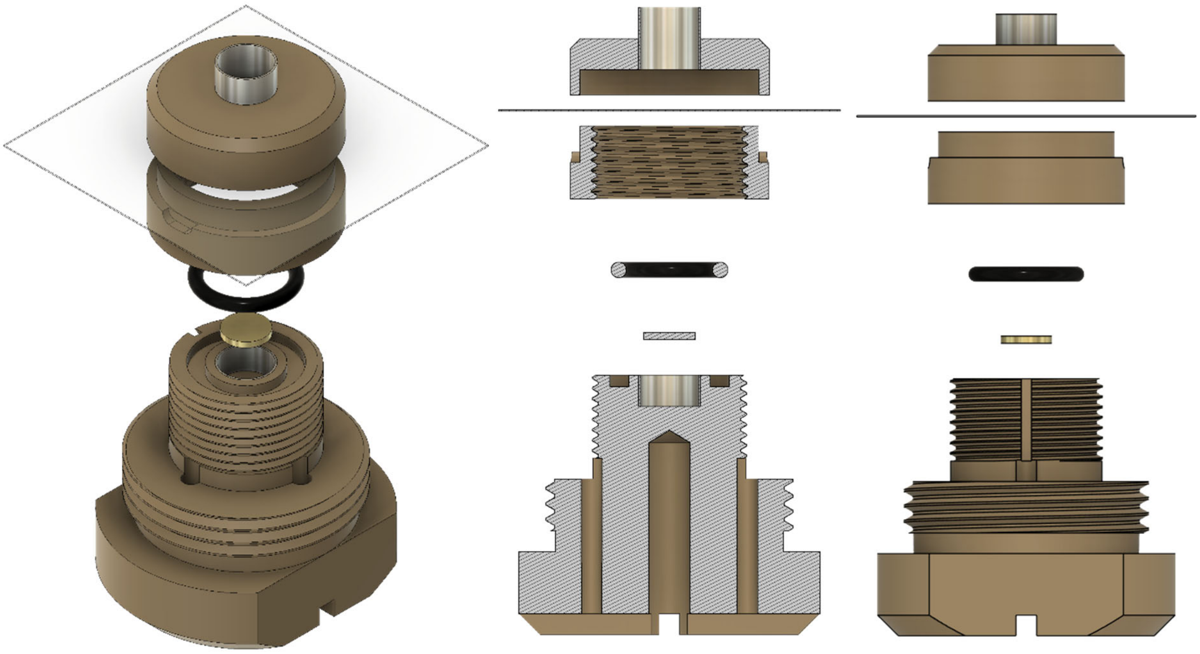

Figure S45. CAD drawings of the frozen solution HFEPR sample holder.

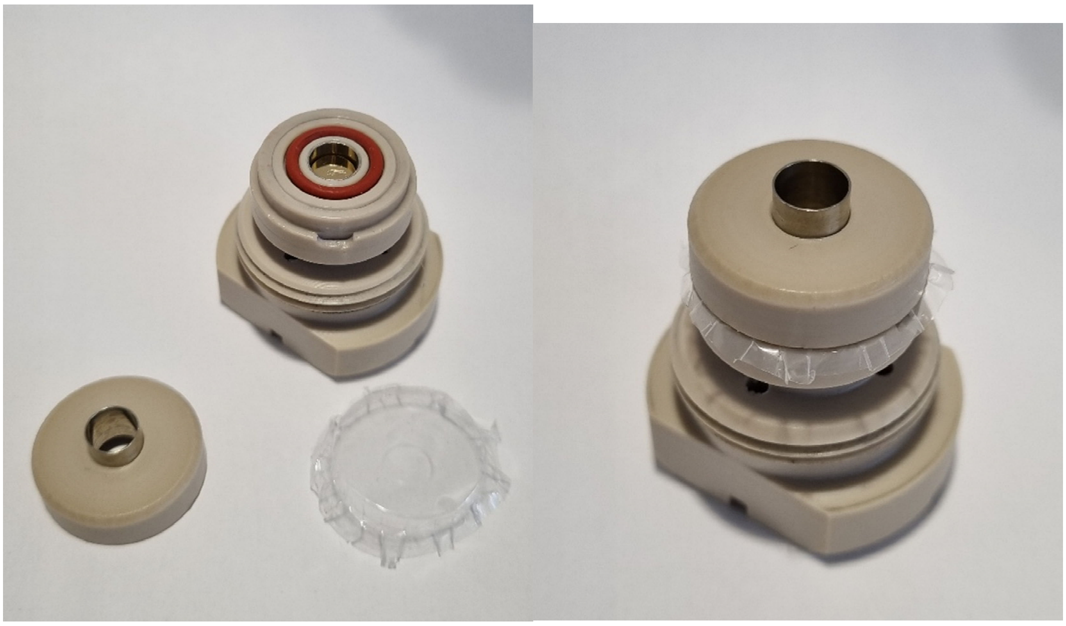

Figure S46. Pictures of the frozen solution HFEPR sample holder.

The sample is placed in a space placed in a the sample holder on top of the gold mirror. The cavity side wall is a german silvertube that is acting as a waveguide. The cavity is enclosed by a thin Mylar foil (0.026 mm) that is pressed by the two part cap and sealed by an rubber O-ring. The sample holder enables measurements in a variaty of solvents in frozen condition as well as liquid state.

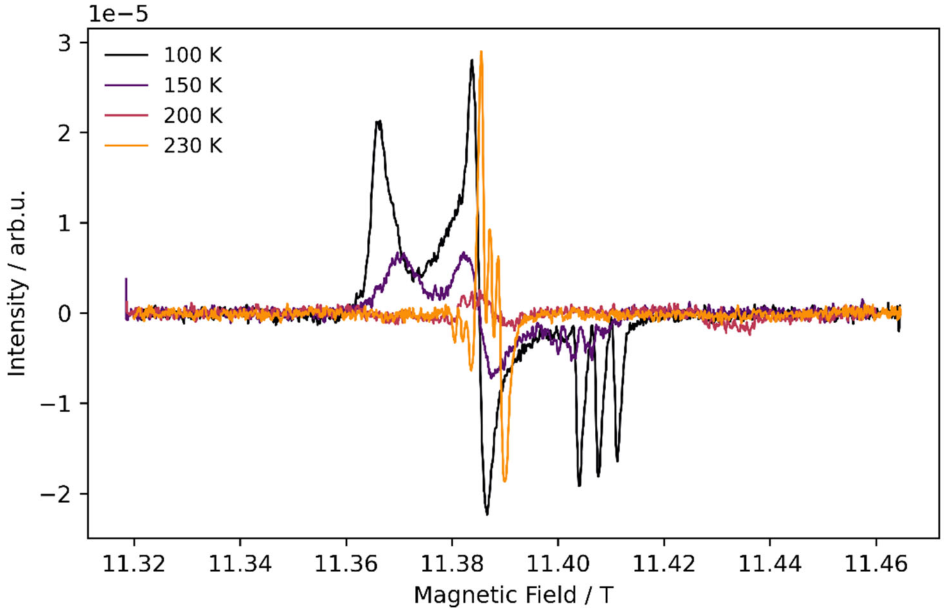

Figure S47. HFEPR measurements of TEMPO in solution (Toluene:DCM) measured with 320 GHz at various temperatures from 100 K (frozen solution) to 230 K (fluid solution).

5 References

(1) Bollmann, A.; Blann, K.; Dixon, J. T.; Hess, F. M.; Killian, E.; Maumela, H.; McGuinness, D. S.; Morgan, D. H.; Neveling, A.; Otto, S.; Overett, M.; Slawin, A. M. Z.; Wasserscheid, P.; Kuhlmann, S. Ethylene tetramerization: a new route to produce 1-octene in exceptionally high selectivities. *J. Am. Chem. Soc.* **2004**, *126*, 14712–14713. DOI: 10.1021/ja045602n.

(2) Samuel, P. P.; Neufeld, R.; Chandra Mondal, K.; Roesky, H. W.; Herbst-Irmer, R.; Stalke, D.; Demeshko, S.; Meyer, F.; Rojisha, V. C.; De, S.; Parameswaran, P.; Stückl, A. C.; Kaim, W.; Christian, J. H.; Bindra, J. K.; Dalal, N. S. Cr(i)Cl as well as Cr+ are stabilised between two cyclic alkyl amino carbenes. *Chem. Sci.* **2015**, *6*, 3148–3153. DOI: 10.1039/C5SC00646E. Published Online: Mar. 20, 2015.

(3) Bhattacharya, S.; Atwi, B.; Kundu, K.; Frey, W.; Buchmeiser, M. R. Chromium(II)/(III) Complexes Bearing Phosphino- N -Heterocyclic Carbene and N -Chelating N -Heterocyclic Carbene Ligands and Their Use in Ethylene Oligomerization. *Organometallics* **2025**, *44*, 315–324. DOI: 10.1021/acs.organomet.4c00458.

(4) Bhattacharya, S.; Allgaier, A.; Frey, W.; van Slageren, J.; Buchmeiser, M. Phenolate- and Alcoholate-Based Chromium NHeterocyclic Carbene Complexes: Structural Peculiarities,

Magnetometric and EPR Study and Catalytic Ethylene Oligomerization. *ChemRxiv* **2025.** DOI: 10.26434/chemrxiv-2025-685nw. Published Online: Nov. 27, 2025.

(5) Neugebauer, P.; Bloos, D.; Marx, R.; Lutz, P.; Kern, M.; Aguilà, D.; Vaverka, J.; Laguta, O.; Dietrich, C.; Clérac, R.; van Slageren, J. Ultra-broadband EPR spectroscopy in field and frequency domains. *Phys. Chem. Chem. Phys.* **2018**, *20*, 15528–15534. DOI: 10.1039/c7cp07443c.

(6) Rabeah, J.; Bauer, M.; Baumann, W.; McConnell, A. E. C.; Gabrielli, W. F.; Webb, P. B.; Selent, D.; Brückner, A. Formation, Operation and Deactivation of Cr Catalysts in Ethylene Tetramerization Directly Assessed by Operando EPR and XAS. *ACS Catal.* **2013**, *3*, 95–102. DOI: 10.1021/cs300686m.

(7) Brückner, A.; Jabor, J. K.; McConnell, A. E. C.; Webb, P. B. Monitoring Structure and Valence State of Chromium Sites during Catalyst Formation and Ethylene Oligomerization by in Situ EPR Spectroscopy. *Organometallics* **2008**, *27*, 3849–3856. DOI: 10.1021/om800316m.

(8) Neese, F. The ORCA program system. *WIREs Comput. Mol. Sci.* **2012**, *2*, 73–78. DOI: 10.1002/wcms.81.

(9) Weigend, F.; Ahlrichs, R. Balanced basis sets of split valence, triple zeta valence and quadruple zeta valence quality for H to Rn: Design and assessment of accuracy. *Phys. Chem. Chem. Phys.* **2005**, *7*, 3297–3305. DOI: 10.1039/B508541A. Published Online: Aug. 4, 2005.

(10) Tanabe, Y.; Sugano, S. On the Absorption Spectra of Complex Ions II. *J. Phys. Soc. Jpn.* **1954**, *9*, 766–779. DOI: 10.1143/JPSJ.9.766.

(11) Tanabe, Y.; Sugano, S. On the Absorption Spectra of Complex Ions. I. *J. Phys. Soc. Jpn.* **1954**, *9*, 753–766. DOI: 10.1143/JPSJ.9.753.

(12) Tanabe, Y.; Sugano, S. On the Absorption Spectra of Complex Ions, III The Calculation of the Crystalline Field Strength. *J. Phys. Soc. Jpn.* **1956**, *11*, 864–877. DOI: 10.1143/JPSJ.11.864.

(13) Stoll, S.; Schweiger, A. EasySpin, a comprehensive software package for spectral simulation and analysis in EPR. *J. Magn. Reson.* **2006**, *178*, 42–55. DOI: 10.1016/j.jmr.2005.08.013. Published Online: Sep. 26, 2005.

(14) Sojka, A.; Sedivy, M.; Lagin, A.; Gabris, A.; Laznicka, T.; Santana, V. T.; Laguta, O.; Neugebauer, P. Sample Holders for Sub-THz Electron Spin Resonance Spectroscopy. *IEEE Trans. Instrum. Meas.* **2022**, *71*, 1–12. DOI: 10.1109/TIM.2022.3164135.
